# Xylochemical Synthesis and Biological Evaluation of Shancigusin C and Bletistrin G ‡

**DOI:** 10.3390/molecules26113224

**Published:** 2021-05-27

**Authors:** Leander Geske, Ulrich Kauhl, Mohamed E. M. Saeed, Anja Schüffler, Eckhard Thines, Thomas Efferth, Till Opatz

**Affiliations:** 1 Department of Chemistry, Organic Chemistry Section, Johannes Gutenberg University, Duesbergweg 10-14, 55128 Mainz, Germany; legeske@uni-mainz.de (L.G.); ulrich.kauhl@gmx.de (U.K.); 2 Institute of Pharmaceutical and Biomedical Sciences, Johannes Gutenberg University, Staudingerweg 5, 55128 Mainz, Germany; saeedm@uni-mainz.de; 3 Institut für Biotechnologie und Wirkstoff-Forschung gGmbH, Hanns-Dieter-Hüsch-Weg 17, 55128 Mainz, Germany; schueffler@ibwf.de (A.S.); thines@ibwf.de (E.T.); 4 Institute for Microbiology, Johannes Gutenberg University, Hanns-Dieter-Hüsch-Weg 17, 55128 Mainz, Germany

**Keywords:** shancigusin C, bletistrin G, total synthesis, biological activity, natural products

## Abstract

The biological activities of shancigusin C (**1**) and bletistrin G (**2**), natural products isolated from orchids, are reported along with their first total syntheses. The total synthesis of shancigusin C (**1**) was conducted by employing the Perkin reaction to forge the central stilbene core, whereas the synthesis of bletistrin G (**2**) was achieved by the Wittig olefination followed by several regioselective aromatic substitution reactions. Both syntheses were completed by applying only renewable starting materials according to the principles of xylochemistry. The cytotoxic properties of shancigusin C (**1**) and bletistrin G (**2**) against tumor cells suggest suitability as a starting point for further structural variation.

## 1. Introduction

Traditional medicine is an almost inexhaustible source for the identification of novel bioactive substances. Phytochemistry as an independent discipline has been engaged in bioactivity-guided isolation of new chemical structures for decades. If pharmacological activities of isolated molecules are demonstrated in vitro, this often serves as an argument to scientifically substantiate their traditional use for certain diseases. Plants produce chemical substances mainly to defend themselves against microbes and predators. Conversely, this means that even plants without ethnopharmacological background and no use in traditional medicine would contain chemical substances that might be pharmacologically active. As of yet, this aspect has remained understudied in phytochemical research.

Therefore, we were interested in the large and heterogeneous group of orchids, which, on the one hand, definitely has ethnopharmacological significance in Asia, Africa and South America. On the other hand, they are mostly rare in Europe and therefore rarely used in folk medicine.

**Figure 1:**
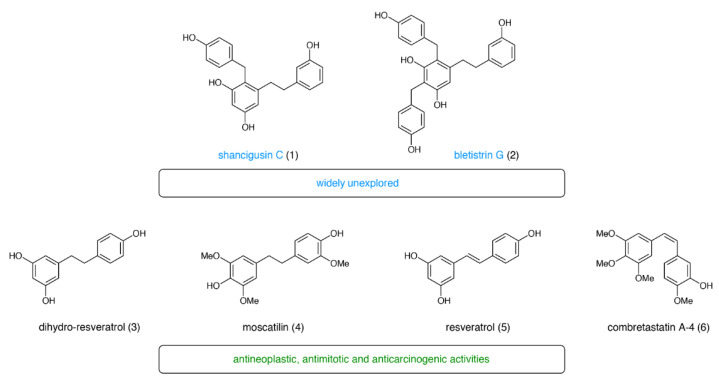
Polyhydroxylated and methoxylated stilbenes and dihydrostilbenes isolated from orchidaceous plants.

Plants from the orchid family have been widely used in the traditional Chinese medicine (TCM) and oriental folk medicines to treat tumors, burns, frostbite, coughs, tuberculosis, bronchitis and gastrointestinal bleeding, as well as to remove “heat and toxin” [1]. According to previous reports, extracts from a variety of different terrestrial orchids contain remarkable amounts of stilbenes and dihydrostilbenes, such as dihydro-resveratrol (**3**), moscatilin (**4**), resveratrol (**5**) and combretastatin A-4 (**6**) [2,3,4,5,6,7,8], which are known to exhibit antineoplastic, antimitotic and anticarcinogenic properties as well as inhibit oxidative stress (Figure 1) [9,10,11,12,13,14,15,16,17,18,19,20,21]. The structurally unprecedented dihydrostilbenes shancigusin C (**1**) and bletistrin G (**2**), both featuring 4-hydroxybenzyl substituents, were isolated from the orchidaceous species *Pleione yunnanensis* and *Bletilla striata* in 2009 and 2019, respectively [6,7]. This subgroup within the class of dihydrostilbenes appears to be widely underexplored concerning their biological activities. Moreover, no total synthetic access has been described so far.

Shancigusin C (**1**) and bletistrin G (**2**) were isolated and identified by us in 2017 from *Himantoglossum hircinum* (L.) Spreng [22]. This plant is a rare orchid that grows in southern regions of Germany on calcareous and stony clay soils. It occurs on dry grasslands, rough pastures and orchards on sloping hillsides. A large number of these plants appeared in a private meadow near a village in the Donnersberg County. This unusual assemblage was probably due to the sheltered location and favorable growing conditions. This gave rise to the idea of harvesting some specimens of the plant for scientific purposes and searching for phytochemical constituents with written permission from the authorities (see the Supporting Information). While the structure elucidation of 1 was first published by Guo in 2009 [6], the structure elucidation of **2** and the total synthesis of both natural products **1** and **2** is reported for the first time in this investigation.

## 2. Results

### 2.1. Structure Elucidation of Bletistrin G (**2**)

**Figure 2:**
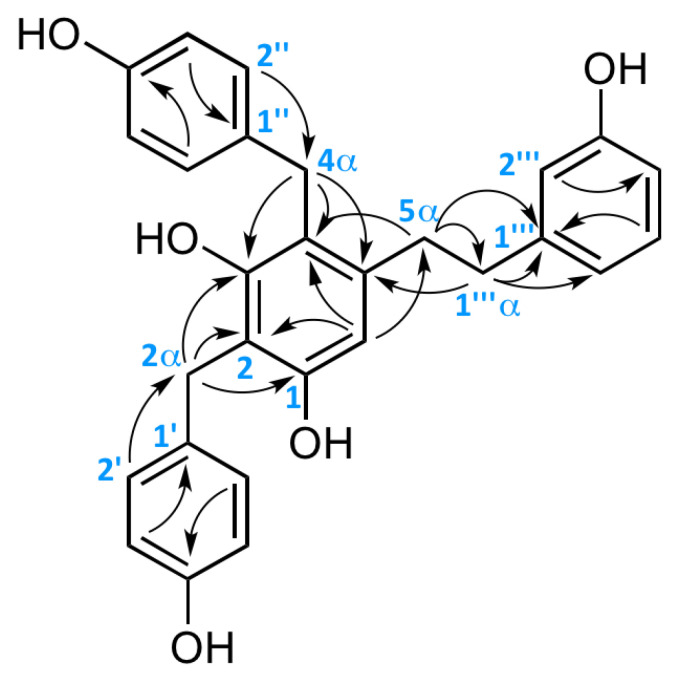
Essential HMBC correlations of bletistrin G (**2**).

The molecular formula of bletistrin G (**2**) was established as C_28_H_26_O_5_ by HR-ESI-MS. The ^1^H-NMR and COSY spectra of **2** exhibited the resonances of two 1,4-substituted aromatic systems bearing oxygen substituents at C-4′ and C-4″ (156.0 and 156.1 ppm). HMBC contacts of both ortho protons (H-2′/H-6′ and H-2″/H-6″) to two different methylene groups (2α-CH_2_ and 4α-CH_2_) determined the presence of two 4-hydroxybenzyl groups. HMBC contacts of 2α-CH_2_/4α-CH_2_ and a single proton at 6.31 ppm (H-6) to the same quaternary carbon atoms C_q_-2 and C_q_-4 (114.9 and 119.0) finally showed the presence of a 2,4,5-trisubstituted resorcinol core bearing two 4-hydroxybenzyl substituents at positions 2 and 4. The resorcinol proton at 6.31 ppm further exhibited an HMBC contact to another methylene group 5α-CH_2_, which was in the vicinity (confirmed by COSY) of another methylene group 1‴α-CH_2_. Since both methylene groups 5α-CH_2_ and 1‴α-CH_2_ showed HMBC contacts to quaternary carbon atoms C_q_-5 and C_q_-1‴ (140.9 and 145.1 ppm), the resorcinol core was unambiguously connected to another aromatic core by an ethano-bridge via its 5-position (Figure 2).

The remaining four aromatic protons which appeared at 7.03 (t, 1H, J = 7.9 Hz, H-5‴), 6.57 (d, 1H, J = 7.9 Hz, H-4‴) and 6.55–6.52 (m, 2H, H-2‴, H-6‴) ppm suggested the presence of a 1,3-disubstituted aromatic ring. HMBC contacts of proton H-5‴ to C_q_-1‴ (145.1 ppm) and C_q_-3‴ (158.3 ppm) finally revealed that the last aromatic core featured a hydroxyl group at C_q_-3‴. The complete signal assignment was confirmed by the COSY, HSQC, HMBC and NOESY spectra. This work of 2017 was reported in the doctoral thesis of U. Kauhl in 2018 [22]. Parallel to our synthetic work, other researchers reported the isolation of bletistrin G (**2**) from rhizomes of *Bletilla striata* and disclosed its structure [7].

### 2.2. Total Synthesis of Shancigusin C (**1**)

**Scheme 1:**
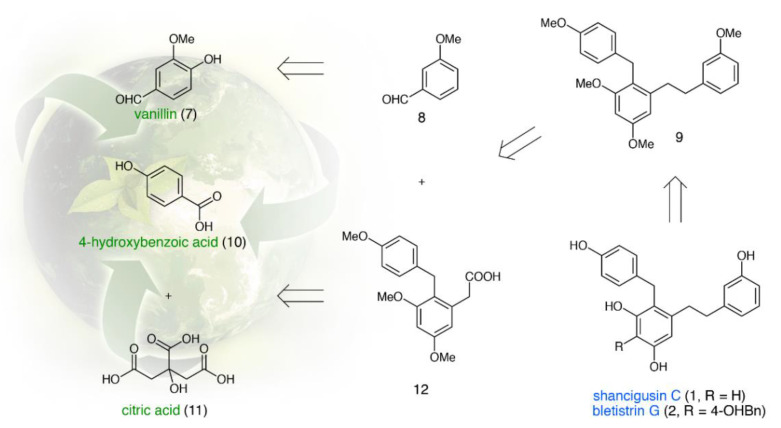
Retrosynthetic studies of the synthesis of shancigusin C (**1**) and bletistrin G (**2**).

Our initial synthesis route for **1** and **2** was developed under the premise that the entire carbon skeleton of both natural products should be synthesized from renewable starting materials according to the principles of xylochemistry [23,24,25,26]. To this end, shancigusin C (**1**) and bletistrin G (**2**) were traced back retrosynthetically to the polymethoxylated dihydrostilbene (**9**), which should be prepared via Perkin condensation of phenylacetic acid (**12**) with 3-methoxybenzaldehyde (**8**, Scheme 1). These two materials are ultimately derivable from citric acid (**11**), one of the most prevalent natural fruit acids [27,28], 4-hydroxybenzoic acid (**10**) and vanillin (**7**), both being readily available products of several lignin depolymerization processes [29,30,31].

**Scheme 2:**
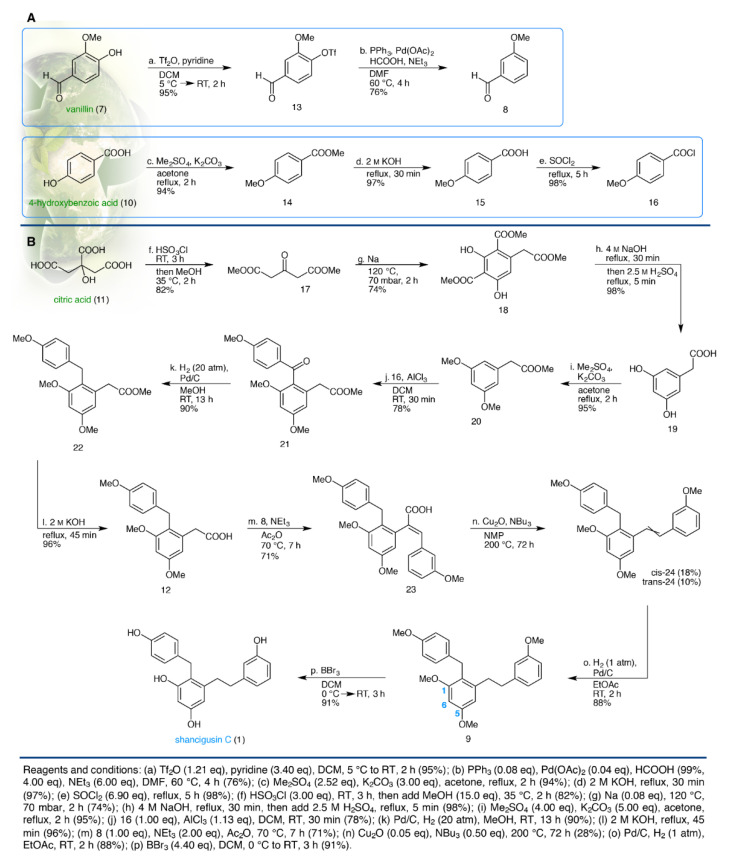
Total synthesis of shancigusin C (**1**). (**A**) Assembly of requisite building blocks **8** and **16**. (**B**) Completion of the 11-step synthesis.

The synthesis started with the preparation of the requisite coupling partner *m*-anisaldehyde (**8**) and *p*-anisoyl chloride (**16**) (Scheme 2A). The aldehyde (**8**) was accessible via two steps starting from lignin-derivable vanillin (**7**), which was converted into a triflate (**13**) with an excellent yield, followed by reductive C-O bond cleavage generating *m*-anisaldehyde (**8**) with a 76% yield. To prepare a chloride (**16**), lignin-derivable 4-hydroxybenzoic acid (**10**) was doubly methylated with dimethyl sulfate (available, e.g., from wood-based methanol) [32] to form an ester (**14**). Subsequent saponification with KOH to *p*-anisic acid (**15**) and treatment with SOCl_2_ then furnished *p*-anisoyl chloride (**16**) with an 89% yield over three steps. In the search for strategies for the construction of a coupling partner (**12**) containing a 3,5-dihydroxyphenyl core, a report by Theilacker [33] suggested the use of dimethyl-1,3-acetonedicarboxylate (**17**) available through decarbonylation from the naturally occurring citric acid (**11**) with an 82% yield (Scheme 2B). Cyclization involving sodium as a catalyst then led to the formation of methyl phenylacetate (**18**) with a 74% yield [33,34]. With **18** in hand, simultaneous saponification of all ester groups followed by acid-induced double decarboxylation generated the desired 3,5-dihydroxyphenylacetic acid (**19**) with an excellent yield. Methylation of **19** using dimethyl sulfate then provided dimethoxylated methyl phenylacetate (**20**) with a 95% yield, which was regioselectively acylated under Friedel–Crafts conditions to furnish a diaryl ketone (**21**) with a 75% yield. The keto group was then reduced to give the corresponding diphenylmethane (**22**), which was saponified producing the desired coupling partner (**12**) with an 86% yield over two steps. Condensation with 8 under Perkin conditions exclusively generated trans-cinnamic acid (**23**) with a 71% yield. In the next step, Cu_2_O-mediated decarboxylation of **23** afforded a cis/trans mixture of stilbenes (**24**) with a combined yield of 28%. The low yield in this step was caused by the cylization of **23** into different inseparable side products. Using hydrogen under ambient pressure, the stilbene **24** could be converted into the corresponding dihydrostilbene **9** with an 88% yield. To complete the total synthesis of shancigusin C (**1**), the methyl ethers were cleaved by BBr_3_, smoothly furnishing the natural product 1 with a 91% yield. In this way, shancigusin C (**1**) could be synthesized with a 6% yield over 11 consecutive steps, starting from citric acid (**11**).

### 2.3. Total Synthesis of Bletistrin G (**2**)

It was anticipated that the chelating effect of the C-1 and C-5 methoxy group of the dihydrostilbene (**9**) (we chose atom numbering based on the IUPAC nomenclature for unknown compounds to prevent confusion with the conventional stilbene numbering system) might favor lithiation at C-6 which would upon trapping with **16** result in an easy access to the main core of bletistrin G (**2**). Unfortunately, all attempts to achieve this lithiation regioselectively failed and in every case, a large number of regioisomeric singly and doubly substituted side products could be detected.

**Scheme 3:**
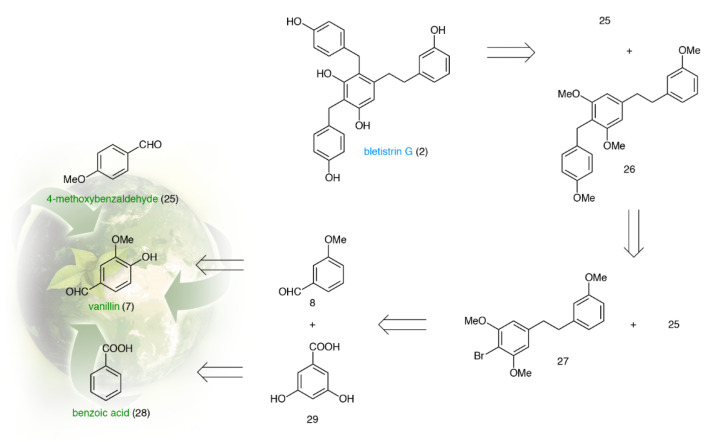
Retrosynthetic aspects for the total synthesis of bletistrin G (**2**).

Hence, the initial synthetic route to **2** was abandoned. It was instead decided to first attach a 4-methoxybenzyl substituent through a reaction with *p*-anisaldehyde (**25**)—available on an industrial scale from naturally occurring anethole—at C-2 of brominated dihydrostilbenes **27**, which should be synthesized starting from vanillin (**7**) and 3,5-dihydroxybenzoic acid (**29**), the latter being accessible with a high yield from the well-known xylochemical benzoic acid (**28**) through double sulfonation and hydrolysis (Scheme 3) [35]. In the last steps, the second 4-methoxybenzyl substituent should be attached to one of the free ortho-positions of **26**.

**Scheme 4:**
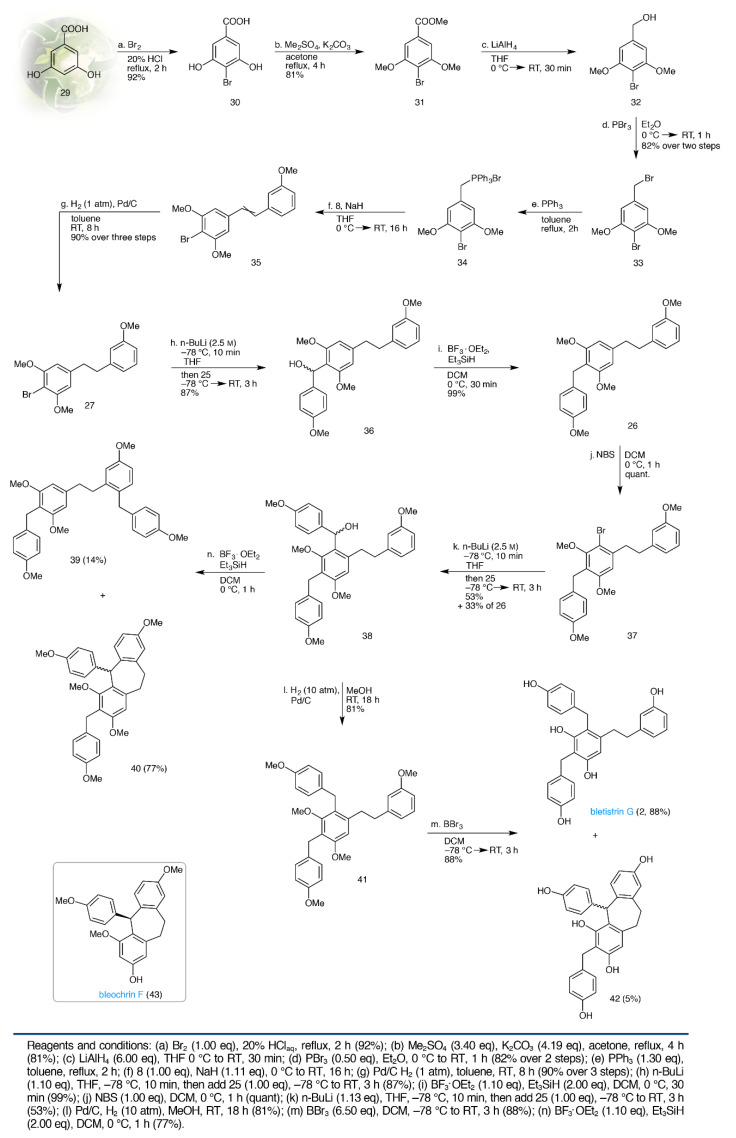
Total synthesis of bletistrin G (**2**) in 13 steps.

The synthesis of **2** began with regioselective bromination of 3,5-dihydroxybenzoic acid (**29**) to get access to the C-4 position. The brominated product **30** was obtained with a 92% yield and further converted into a dihydrostilbene **27** over six consecutive steps (Scheme 4). First, methylation of **30** afforded methyl benzoate (**31**) with an 81% yield, which was reduced to alcohol **32** using LiAlH_4_. Upon treatment with PBr_3_, the corresponding benzyl bromide **33** was obtained with an 82% yield over two steps. Conversion of **33** into a phosphonium salt **34** and the Wittig reaction with *m*-anisaldehyde (**8**) afforded a cis/trans-mixture of stilbenes **35**, which were hydrogenated to give the desired dihydrostilbene **27** with a 90% yield over three steps, retaining the bromine substituent. With ample amounts of **27** at hand, benzhydrol **36** was prepared by the use of *n*-BuLi/*p*-anisaldehyde (**25**) and subsequently reduced using Et_3_SiH to furnish **26** with an 86% yield over two steps. In the next step, the ortho position of **26** was brominated, generating **37** with a quantitative yield. Here, the use of dichloromethane as a solvent at low temperatures was of great importance since at higher temperatures or in other solvents, unselective bromination dominated. After halogen/lithium exchange and quenching the intermediate lithium salt with *p*-anisaldehyde (**25**), alcohol **38** was produced with a 53% yield. The major side product in this reaction was a dihydrostilbene **26**, which could be recycled by flash chromatography with a 33% yield. Acid-promoted reduction of the alcohol **38** this time did not furnish a dihydrostilbene **41**, but led to the formation of **39** and dibenzocycloheptane **40**. The latter represents the core structure of bleochrin F (**43**), another natural product isolated from orchids, which has not been synthesized so far. To achieve reduction without cyclization, compound **38** was to produce the desired dihydrostilbene **41** with an 81% yield. At this stage, all that remained to be done was the cleavage of the methyl ethers, which was again completed by the use of BBr_3_ affording bletistrin G (**2**) and another cyclized product, **42**, with an 88% and 5% yield, respectively. In this way, bletistrin G (**2**) could be synthesized with an 18% yield over 13 consecutive steps, starting from benzoic acid (**29**). Both side products **39** and **42** are most likely generated via hydride transfer reactions predestined for diarylmethanes and diarylmethanols [36]. In the latter case, either the solvent (DCM) or the bromomethane liberated from *O*-demethylation could have acted as the hydride acceptors under the strongly Lewis acidic reaction conditions.

### 2.4. Biological Results

**Figure 3:**
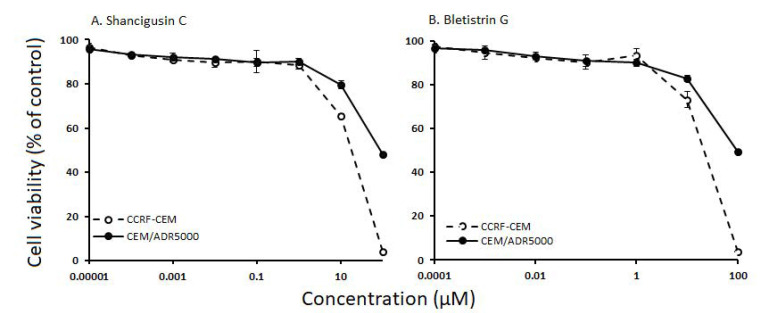
Cytotoxicity of shancigusin C (**1**) and bletistrin G (**2**) towards human sensitive CCRF-CEM and multidrug-resistant CEM/ADR5000 leukemia cells.

The growth inhibitory activity of both synthetic compounds was investigated in wildtype drug-sensitive CCRF-CEM tumor cells and in multidrug-resistant CEM/ADR5000 cells by means of the resazurin assay. As depicted in Figure 3, both compounds inhibited both cell lines, albeit at different concentrations.

The growth of CCRF-CEM cells was inhibited by 50% at the shancigusin C (**1**) concentration of 17.9 ± 0.6 µM, while 87.2 ± 9.6 µM were necessary to inhibit the growth of CEM/ADR5000 cells by half. The corresponding degree of resistance was 4.87.

Bletistrin G (**2**) inhibited the CCRF-CEM cell growth with an IC_50_ value of 21.6 ± 3.0 µM and the CEM/ADR5000 cell growth with IC_50_ of 96.2 ± 2.1 µM. Hence, the CEM/ADR5000 cells were 4.45-fold more cross-resistant to bletistrin G (**2**).

## 3. Discussion

In this study, we isolated shancigusin C (**1**) and bletistrin G (**2**) from the protected species *Himantoglossum hircinum* and described their total chemical synthesis.

The cytotoxic properties of both compounds against tumor cells are indicative of their pharmacological activity and potential suitability as lead compounds for derivatization of new candidate compounds with improved pharmacological features for tumor therapy. The so-called multidrug-resistant phenotype, which is mediated by ATP-binding cassette (ABC) transporters such as P-glycoprotein, is a particularly feared phenomenon of resistance to established cytostatic drugs [37]. Our analyses indicate that multidrug-resistant CEM/ADR5000 cells were also cross-resistant to shancigusin C (**1**) and bletistrin G (**2**). However, the degrees of cross-resistance were low (resistance levels below 5), whereas resistance to the standard drug doxorubicin was above 1000. High-grade cross-resistance was also measured in CEM/ADR5000 cells towards other established cytostatic drugs (other anthracyclines, Vinca alkaloids, taxanes, etc.) [38]. This suggests that shancigusin C (**1**) and bletistrin G (**2**) have some potential to inhibit not only sensitive but also multidrug-resistant tumor cells.

In summary, by applying only sustainable starting materials to construct the entire carbon skeleton of the products, the first total syntheses of the unusual natural products shancigusin C (**1**) and bletistrin G (**2**) were executed in 11 and 13 steps, respectively. As most of the reactions can be run on a gram scale with moderate to very high yields, the described synthetic pathways should be amenable to the synthesis of related natural products allowing for the evaluation of their yet unknown biological activities.

This investigation also supports the concept that chemical synthesis of bioactive compounds (e.g., of shancigusin C (**1**) and bletistrin G (**2**)) derived from rare plants represents an effective means to protect plants from extinction because chemical synthesis prevents harvesting of plant materials from the wild in order to obtain sufficient materials for biological investigations or even preclinical drug development.

## 4. Materials and Methods

Unless otherwise stated, all reagents and solvents were obtained from commercial suppliers (Sigma-Aldrich (St. Louis, MI, USA), Alfa Aesar (Haverhill, MA, USA), TCI chemicals (Tokyo, Japan), ABCR (Karlsruhe, Germany), Acros Organics (Fair Lawn, NJ, USA) and Fisher Scientific (Waltham, MA, USA)) and used as provided without prior purification. Anhydrous diethyl ether_,_ tetrahydrofuran and toluene were dried with an MBraun solvent purification system 5. Dichloromethane was obtained by distillation from calcium hydride under argon atmosphere. Flash column chromatography was performed using silica gel (35–70 μm, Acros Organics). Analytical thin-layer chromatography (TLC) was done on Merck silica gel plates (60 F_254_) with defined solvent mixtures and visualized under UV light irradiation and/or TLC staining reagents. Melting points were determined in open capillary tubes and are uncorrected. IR spectra were measured with a Tensor 27 with a diamond ATR unit from Bruker (Billerica, MA, USA) and are reported in terms of frequency of absorption (*ν*, cm^–1^). NMR spectra were recorded on a Bruker Avance-III HD (^1^H-NMR: 300 MHz, ^13^C-NMR: 75.5 MHz), a Bruker Avance-II (^1^H-NMR: 400 MHz, ^13^C-NMR: 100.6 MHz) or a Bruker Avance-III (^1^H-NMR: 600 MHz, ^13^C-NMR: 151.1 MHz) spectrometer. Chemical shifts are referenced to residual solvent signals (CDCl_3_: 7.26 ppm and 77.16 ppm, DMSO-d_6_: 2.50 ppm and 39.52 ppm, (CD_3_)_2_CO: 2.05 ppm and 27.98 ppm, D_2_O: 4.79 ppm, MeOD: 3.31 ppm and 49.00 ppm for ^1^H-NMR, COSY and ^13^C-NMR, HSQC, HMBC respectively) and reported in parts per million (ppm) relative to tetramethylsilane (TMS). Electron spray ionization (ESI) mass spectra were recorded on a 1200-series HPLC-system or a 1260- series Infinity II HPLC-system (Agilent-Technologies, Santa Clara, CA, USA) with binary pump and integrated diode array detector coupled to a LC/MSD-Trap-XTC-mass spectrometer (Agilent-Technologies) or a LC/MSD Infinitylab LC/MSD (G6125B LC/MSD). High resolution mass spectra were recorded on a Micromass-Q-TOF-Ultima-3-mass spectrometer (Waters, Milford, MA, USA) with LockSpray-interface and a suitable external calibrant.

### 4.1. Biological Part

*Himantoglossum hircinum* grew wild on a private meadow in the Donnersberg county (Rhineland-Palatinate). Botanical verification was performed by one of the authors (T.E.). The plant was harvested for scientific reasons with written permission of the Structural and Approval Directorate South, Rhineland Palatine (Struktur- und Genehmigungsdirektion Süd, Rheinland-Pfalz) according to §45, paragraph 7, no. 3 BNatSchG (AZ 42/553-251, letter dated from 19 February 2015). A voucher specimen has been deposited at the Department of Pharmaceutical Biology (Institute of Pharmaceutical Biology, Johannes Gutenberg University, Mainz, Germany).

The dried biomass of the aerial parts of *Himantoglossum hircinum* (130.4 g) was extracted repeatedly with acetone (5×) at 22 °C to yield a concentrated crude extract of 10.5 g. This crude extract was adsorbed to silica gel 60 (Merck, 0.015–0.040 mm), dried and used for flash chromatography on silica gel 60. A solvent gradient was used for separation starting at 100% cyclohexane to 100% ethyl acetate to 100% methanol. In the intermediate (128.3 mg) eluted at 1:1 cyclohexane/ethyl acetate the desired compounds were detected. Preparative HPLC with this intermediate (two-step MeCN/H2O gradient, 23% MeCN to 31.8% MeCN in 19 min, 31.8% MeCN to 34% in 14 min, 21 mL/min, Agilent Eclipse XDB-Phenyl, 5 µm, 21.2 × 150 mm, 22 °C) yielded shancigusin C (9.9 mg, RT 17.7 min) and bletistrin G (3.7 mg, RT 28.8 min).

### 4.2. Biological Evaluation

Wild type CCRF-CEM and multidrug-resistant CEM/ADR5000 cells have been obtained from Dr. Axel Sauerbrey, Department of Pediatrics, University Hospital Jena, Jena, Germany). The development of CEM/ADR5000 by doxorubicin selection, the overexpression of multidrug-resistance-conferring P-glycoprotein and their maintenance in cell culture have been described [39,40,41].

The growth inhibitory potential of these two cell lines has examined by using the resazurin viability assay [42]. The test principle is based on the reduction of the colorless resazurin to the highly fluorescent resorufin in viable cells. Upon exposure to cytotoxic compounds, the fluorescence intensity decreases in a dose-dependent manner. The fluorescence was measured using an Infinite M2000 Pro plate reader (Tecan, Crailsheim, Germany). The performance of this assay in our hands has been reported [43,44].

## Data Availability

Not applicable.

## Supplementary Materials

The following are available online: Synthetic procedures and analytical data, crystallographic data and structure refinement for 1, NMR spectra, references for experimental section.

## Sample Availability

Not available.
