# Supplementary material for: Xylochemical Synthesis and Biological Evaluation of Shancigusin C and Bletistrin G
Source: Molecules. 2021 May 27;26(11):3224. doi: 10.3390/molecules26113224 (PMC8198954; doi:10.3390/molecules26113224)
Supplement: Supplementary file 1 [file molecules-26-03224-s001.zip › molecules-1229113-supplementary.pdf]

molecules

MDPI

Supplementary Material

Xylochemical Synthesis and Biological Evaluation of Shancigusin C and Bletistrin G

Leander Geske [1], Ulrich Kauhl [1], Mohamed E. M. Saeed [2], Anja Schüffler [3,4], Eckhard Thines[3,4] , Thomas Efferth[2,*] and Till Opatz [1,*]

[1] Department of Chemistry, Organic Chemistry Section, Johannes Gutenberg University, Duesbergweg 10–14, 55128 Mainz, Germany; legeske@uni-mainz.de (L.G.); ulrich.kauhl@gmx.de (U.K.)
[2] Institute of Pharmaceutical and Biomedical Sciences, Johannes Gutenberg University, Staudingerweg 5, 55128 Mainz, Germany; saeedm@uni-mainz.de (M.S.)
[3] Institut für Biotechnologie und Wirkstoff-Forschung gGmbH, Hanns-Dieter-Hüsch-Weg 17, 55128 Mainz, Germany; schueffler@ibwf.de (A.S.); thines@ibwf.de (E.T.)
[4] Institute for Microbiology, Johannes Gutenberg University, Hanns-Dieter-Hüsch-Weg 17, 55128 Mainz, Germany; schueffler@ibwf.de (A.S.); thines@ibwf.de (E.T.)
* Correspondence: opatz@uni-mainz.de (T.O.); efferth@uni-mainz.de (T.E.); Tel.: +49-(0)6131-39-22272 (T.O.); +49-(0)6131-39-25751 (T.E.)

1. Syntheses and analytical data of the compounds 1
2. Crystallographic data and structure refinement for shancigusin C (1) 17
3. NMR Spectra of the compounds 19
4. References **52**

1. Syntheses and analytical data of the compounds

*1.1 4-Formyl-2-methoxyphenyl trifluoromethanesulfonate (**13**)*

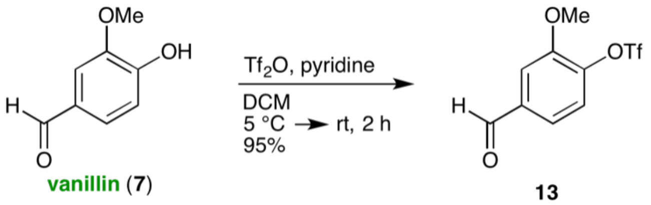

This compound was synthesized using a modified procedure by Tuck [1]. Under argon atmosphere, Tf_2O (0.67 mL, 3.98 mmol, 1.21 eq) was added dropwise to a solution of vanillin (**7**, 0.50 g, 3.29 mmol, 1.00 eq) and pyridine (0.90 mL, 11.2 mmol, 3.40 eq) in DCM (7 mL) by maintaining the temperature below 5 °C. The mixture was stirred at room temperature for 2 h and filtered over a short plug of silica. After removal of the solvent under reduced pressure, the title compound was obtained as a light-yellow oil (0.89 g, 3.11 mmol, 95%).

R_f: 0.27 (SiO_2, ^{c}Hex/EtOAc 9:1).

^{1}H-NMR, COSY (300.1 MHz, $CDCl_3$): δ/ppm = 9.98 (s, 1H, C*H*O), 7.56 (d, 1H, *J* = 1.8 Hz, *H*-3), 7.51 (dd, 1H, *J* = 8.1 Hz, *J* = 1.8 Hz, *H*-5), 7.41 (d, 1H, *J* = 8.1 Hz, *H*-6), 3.99 (s, 3H, OC*H*3).

^{13}C-NMR, HSQC, HMBC (75.5 MHz, $CDCl_3$): δ/ppm = 190.5 (CHO), 152.4 (C_q-2), 142.8 (C_q-1), 136.9 (C_q-4), 124.2 (*C*-5), 123.3 (*C*-6), 118.8 (q, *J* = 320.1 Hz, *C*F3), 111.9 (*C*-3), 56.6 (OCH_3).

^{19}F-NMR (282.4 MHz, $CDCl_3$): δ/ppm = –74.9 (s, 3F).

IR: $\bar{\nu}$ [cm^{-1}] = 2948, 2858, 1707, 1605, 1500, 1424, 1210, 1140, 1107, 1029, 876, 616.

The analytical data are consistent with those reported in the literature [1].

*1.2 3-Methoxybenzaldehyde (**8**)*

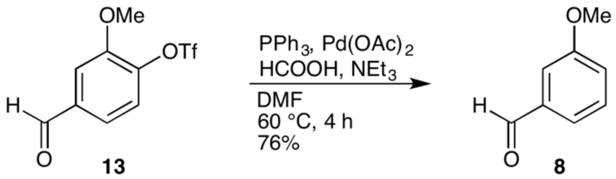

The title compound was synthesized using a modified procedure by Node [2]. Under nitrogen atmosphere, PPh_3 (7.92 mg, 0.03 mmol, 0.04 eq) and $Pd(OAc)_2$ (3.51 mg, 0.02 mmol, 0.02 eq) were added to a solution of 4-formyl-2-methoxyphenyl trifluoromethanesulfonate (**13**, 219 mg, 0.77 mmol, 1.00 eq) in DMF (6 mL). After adding HCOOH (99%, 0.06 mL, 1.54 mmol, 2.00 eq) and NEt_3 (0.32 mL, 2.31 mmol, 3.00 eq), the mixture was stirred at 60 °C for 2 h. The same amount of PPh_3, $Pd(OAc)_2$, HCOOH and NEt_3 was added to the mixture and stirring was prolonged for further 2 h. EtOAc (20 mL) was added and the organic phase was washed with a saturated solution of NaCl (20 mL), dried over Na_2SO_4 and the solvent was removed under reduced pressure. After flash-chromatographic purification on silica (^{c}Hex/EtOAc 15:1), the title compound was obtained as a light-yellow liquid (79.6 mg, 0.58 mmol, 76%).

R_f: 0.33 (SiO_2, ^{c}Hex/EtOAc 10:1).

^{1}H-NMR, COSY (300.1 MHz, $CDCl_3$): δ/ppm = 9.98 (s, 1H, C*H*O), 7.50–7.42 (m, 2H, *H*-5, *H*-6), 7.41–7.38 (m, 1H, *H*-2), 7.22–7.14 (m, 1H, *H*-4) 3.87 (s, 3H, OC*H*3).

^{13}C-NMR, HSQC, HMBC (75.5 MHz, $CDCl_3$): δ/ppm = 192.3 (CHO), 160.3 (C_q -3), 137.9 (C_q-1), 130.2 (*C*-5), 123.7 (*C*-6), 121.7 (*C*-4), 112.1 (*C*-2), 56.6 (OCH_3).

IR: $\bar{\nu}$ [cm^{-1}] = 2944, 2840, 2730, 1702, 1597, 1587, 1486, 1285, 1264, 1040, 789, 773.

The analytical data are consistent with those reported in the literature [2].

*1.3 Methyl 4-methoxybenzoate (**14**)*

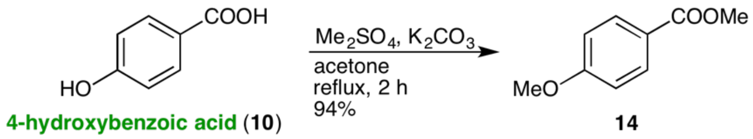

This compound was synthesized using a modified procedure by Usuki [3]. To a solution of 4-hydroxybenzoic (**10**, 3.00 g, 21.7 mmol, 1.00 eq) in acetone (30 mL) was added K_2CO_3 (9.00 g, 65.1 mmol, 3.00 eq) and dimethyl sulfate (5.19 mL, 54.7 mmol, 2.52 eq) and the suspension was refluxed for 2 h. After cooling to room temperature, the mixture was filtered and the solvent was removed under reduced pressure. The residue was taken up with water (50 mL) and extracted with EtOAc (3 x 30 mL). The combined organic layers were washed with a saturated solution of NaCl (30 mL), dried over Na_2SO_4 and after removal of the solvent under reduced pressure the title compound was obtained as a colorless solid (3.38 g, 20.3 mmol, 94%).

Mp.: 49.2–49.6 °C ($CHCl_3$), Lit.: 48–50 °C (aqueous NaHCO3) [4].

R$_f$: 0.62 (SiO_2, ^{c}Hex/EtOAc 2:1).

^{1}H-NMR, COSY (300.1 MHz, $CDCl_3$): δ/ppm = 8.03–7.96 (m, 2H, *H*-2, *H*-6), 6.95–6.88 (m, 2H, *H*-3, *H*-5), 3.88 (s, 3H, COOC*H*$_3$), 3.85 (s, OC*H*$_3$).

^{13}C-NMR, HSQC, HMBC (75.5 MHz, $CDCl_3$): δ/ppm = 167.0 (*C*OOCH$_3$), 163.4 (C$_q$-4), 131.7 (2C, *C*-2, *C*-6), 122.7 (C$_q$-1), 113.7 (2C, *C*-3, *C*-5), 55.6 (O*C*H3), 52.0 (COO*C*H3).

IR: $\bar{\nu}$ [cm^{-1}] = 3003, 2953, 28411, 1711, 1605, 1511, 1434, 1252, 1167, 1102, 847, 769.

The analytical data are consistent with those reported in the literature [4].

*1.4 4-Methoxybenzoic acid (**15**)*

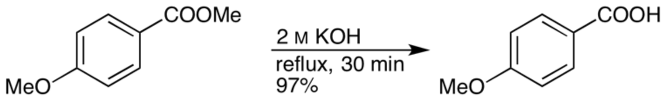

14 **15**

This compound was synthesized using a modified procedure by Pan and She [5]. A solution of methyl 4-methoxybenzoate (**14**, 3.36 g, 20.2 mmol, 1.00 eq) in 2 M KOH_{aq} (30 mL) was refluxed for 30 min. After cooling to room temperature, the solution was acidified to pH 1 by the dropwise addition of HCl_{aq} (c = 2 mol/L) and extracted with EtOAc (3 x 30 mL). The combined organic layers were washed with a saturated solution of NaCl (30 mL), dried over Na_2SO_4 and after removal of the solvent under reduced pressure the title compound was obtained as a colorless solid (2.97 g, 19.6 mmol, 97%).

Mp.: 184.3–185.1 °C ($CHCl_3$), Lit.: 181–182 °C (DCM) [6].

R$_f$: 0.24 (SiO_2, ^{c}Hex/EtOAc 1:1).

^{1}H-NMR, COSY (300.1 MHz, DMSO-d$_6$): δ/ppm = 12.62 (COO*H*), 7.95–7.83 (m, 2H, *H*-2, *H*-6), 7.07–6.96 (m, 2H, *H*-3, *H*-5), 3.82 (s, 3H, OC*H*$_3$).

^{13}C-NMR, HSQC, HMBC (75.5 MHz, DMSO-d$_6$): δ/ppm = 167.0 (*C*OOH), 162.8 (*C*$_q$-4), 131.3 (2C, *C*-2, *C*-6), 123.0 (*C*$_q$-1), 113.8 (2C, *C*-3, *C*-5), 55.5 (O*C*H$_3$).

IR: $\bar{\nu}$ [cm^{-1}] = 2956, 2923, 2870, 2667, 2556, 1685, 1604, 1299, 1263, 1168, 1026, 772.

The analytical data are consistent with those reported in the literature [6].

*1.5 4-Methoxybenzoyl chloride (**16**)*

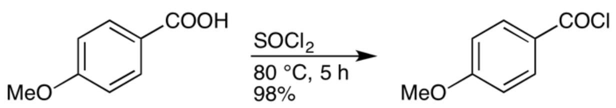

15 **16**

This compound was synthesized using a modified procedure by Keenan [7]. A solution of 4-methoxybenzoic acid (**15**, 1.52 g, 10.0 mmol, 1.00 eq) in $SOCl_2$ (5.00 mL, 69.0 mmol, 6.90 eq) was refluxed for 5 h. After cooling to room temperature, residual $SOCl_2$ was removed under reduced pressure and DCM (10 mL) was added. The solution was stirred for 1 min and the solvent was removed under reduced pressure. After repeating this procedure three times, the title compound was obtained as a light-yellow liquid (1.67 g, 9.78 mmol, 98%).

R$_f$: 0.66 (SiO_2, ^{c}Hex/EtOAc 2:1 + 1% AcOH).

^{1}H-NMR, COSY (300.1 MHz, $CDCl_3$): δ/ppm = 8.12–8.05 (m, 2H, *H*-2, *H*-6), 7.00–6.93 (m, 2H, *H*-3, *H*-5), 3.90 (s, 3H, OC*H*$_3$)

^{13}C-NMR, HSQC, HMBC (75.5 MHz, $CDCl_3$): δ/ppm = 167.3 (*C*OCl), 165.5 (*C*$_q$-4), 134.2 (2C, *C*-2, *C*-6), 125.6 (*C*$_q$-1), 114.4 (2C, *C*-3, *C*-5), 55.9 (O*C*H$_3$).

IR: $\bar{\nu}$ [cm^{-1}] = 2937, 2843, 1765, 1734, 1597, 1574, 1506, 1263, 1211, 1159, 1023, 870.

The analytical data are consistent with those reported in the literature [8].

*1.6 Dimethyl 3-oxopentanedioate (**17**)*

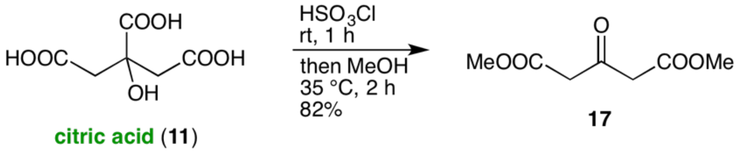

This compound was prepared according to a procedure by Adams [9].

The title compound was obtained as a colorless liquid (4.32 g, 24.8 mmol, 82%).

BP: 127–129 °C (7–8 mbar), Lit.: 138–140 °C (24 mbar) [10].

R_f: 0.21 (SiO_2, ^{c}Hex/EtOAc 10:1).

^{1}H-NMR, COSY (300.1 MHz, $CDCl_3$): δ/ppm = 12.0 (s, 0.1H, Enol-O*H*), 5.12 (s, 0.1H, Enol-C*H*), 3.72 (s, 6H, 2 x OC*H*$_3$), 3.71 (s, 0.6H, 2 x Enol-OC*H*$_3$), 3.60 (s, 4H, 2 x C*H*$_2$), 3.21 (s, 0.2H, Enol-C*H*$_2$).

^{13}C-NMR, HSQC, HMBC (75.5 MHz, $CDCl_3$): δ/ppm = 195.3 (*C*O), 172.7 (Enol-CH*C*O), 169.8 (Enol-*C*$_q$OH), 168.5 (Enol-CH_2*C*O), 167.2 (2C, 2 x *C*OOCH_3), 91.9 (Enol-*C*HCO), 52.6 (2C, 2 x COO*C*H$_3$), 51.5 (2C, 2 x Enol-O*C*H$_3$), 48.8 (2C, 2 x *C*H$_2$), 40.8 (Enol- *C*H$_2$CO).

IR: $\bar{\nu}$ [cm^{-1}] = 3005, 2958, 2850, 1736, 1716, 1437, 1328, 1259, 1197, 1148, 1017, 630.

The analytical data are consistent with those reported in the literature [10,11].

*1.7 Dimethyl 2,4-dihydroxy-6-(2-methoxy-2-oxoethyl)isophthalate (**18**)*

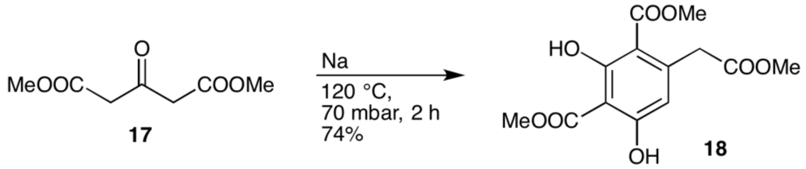

This compound was prepared according to a procedure by Theilacker [12].

The title compound was obtained as colorless needles (28.9 g, 97.1 mmol, 74%). Note: The exact amount of sodium and performing the reaction under reduced pressure to distil off generated MeOH and H_2O is highly important for achieving good yields.

Mp.: 142.9–143.8 °C (MeOH), Lit.: 144–145 °C (MeOH) [13].

R_f: 0.21 (SiO_2, ^{c}Hex/EtOAc 4:1).

^{1}H-NMR, COSY (300.1 MHz, $CDCl_3$): δ/ppm = 12.97 (s, 1H, 2-O*H*), 12.02 (s, 1H, 4-O*H*), 6.38 (s, 1H, *H*-5), 4.02 (s, 3H, 3-COOC*H*$_3$), 3.87 (s, 3H, 1-COOC*H*$_3$), 3.81 (s, 2H, C*H*$_2$), 3.69 (s, 3H, CH_2COOC*H*$_3$).

^{13}C-NMR, HSQC, HMBC (75.5 MHz, $CDCl_3$): δ/ppm = 171.3 (3-*C*OOCH$_3$), 171.1 (2C, 2-*C*OOCH$_3$, CH_2*C*OOCH$_3$), 166.9 (*C*$_q$-4), 166.1 (*C*$_q$-2), 113.8 (*C*-5), 105.2 (*C*$_q$-1), 101.3 (*C*$_q$-3), 53.0 (3-COO*C*H$_3$), 52.3 (1-COO*C*H$_3$), 52.2 (CH_2COO*C*H$_3$), 43.1 (*C*H$_2$).

IR: $\bar{\nu}$ [cm^{-1}] = 3414, 3007, 2956, 2849, 1736, 1657, 1435, 1333, 1252, 1166, 1061, 820.

The analytical data are consistent with those reported in the literature [13].

*1.8 2-(3,5-Dihydroxyphenyl)acetic acid (**19**)*

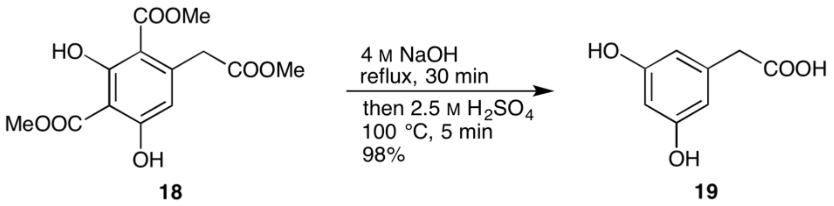

This compound was prepared according to a procedure by Kunz [13].

The title compound was obtained as a light-brown solid (884 mg, 5.25 mmol, 98%).

Mp.: 125.7–126.4 °C ($CHCl_3$), Lit.: 127–128 °C (CHCl3) [14].

R_f: 0.59 (SiO_2, MeOH/EtOAc 1:10).

^{1}H-NMR, COSY (300.1 MHz, D_2O): δ/ppm = 6.29 (d, 2H, *J* = 2.2 Hz, *H*-2, *H*-6), 6.26 (t, 1H, *J* = 2.2 Hz, *H*-4), 3.49 (s, 2H, C*H*$_2$).

^{13}C-NMR, HSQC, HMBC (75.5 MHz, D_2O): δ/ppm = 176.3 (*C*OOH), 156.8 (2C, *C*$_q$-3, *C*$_q$-5), 136.8 (*C*$_q$-1), 108.5 (2C, *C*-2, *C*-6), 101.3 (*C*-4), 40.2 (CH_2).

IR: $\bar{v}$[cm^{-1}] = 3272, 1702, 1599, 1455, 1336, 1303, 1203, 1146, 1049, 1008, 838, 633.

The analytical data are consistent with those reported in the literature [13].

Multigram quantities of **19** (8.73 g, 51.9 mmol) could be obtained in 87% yield starting from **18** (17.8 g, 59.7 mmol).

*1.9 Methyl 2-(3,5-Dimethoxyphenyl)acetate (**20**)*

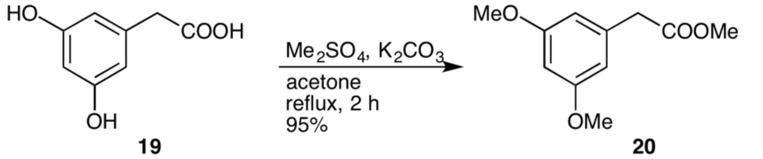

This compound was synthesized using a modified procedure by Pan and She [5]. To a solution of 2-(3,5-dihydroxyphenyl)acetic acid (**19**, 7.70 g, 45.8 mmol, 1.00 eq) in acetone (160 mL) was added K_2CO_3 (31.6 g, 229 mmol, 5.00 eq) and Me_2SO_4 (17.4 mL, 183 mmol, 4.00 eq) and the suspension was refluxed for 2 h. After cooling to room temperature, the mixture was filtered and the solvent was removed under reduced pressure. After flash-chromatographic purification on silica (^{c}Hex/EtOAc 8:1), the title compound was obtained as a colorless oil (9.15 g, 43.5 mmol, 95%).

R$_f$: 0.43 (SiO_2, ^{c}Hex/EtOAc 4:1).

^{1}H-NMR, COSY (300.1 MHz, $CDCl_3$): δ/ppm = 6.44 (d, 2H, *J* = 2.2 Hz, *H*-2, *H*-6), 6.37 (t, 1H, *J* = 2.2 Hz, *H*-4), 3.78 (s, 6H, 2 x OC*H*$_3$), 3.69 (s, 3H, COOC*H*$_3$), 3.56 (s, 2H, C*H*$_2$).

^{13}C-NMR, HSQC, HMBC (75.5 MHz, $CDCl_3$): δ/ppm = 171.9 (*C*OOCH$_3$), 161.0 (2C, *C*$_q$-3, *C*$_q$-5), 136.1 (*C*$_q$-1), 107.4 (2C, *C*-2, *C*-6), 99.3 (*C*-4), 55.4 (2C, 2 x O*C*H$_3$), 52.2 (COO*C*H$_3$), 41.6 (*C*H$_2$).

IR: $\bar{v}$[cm^{-1}] = 3001, 2952, 2840, 1735, 1595, 1431, 1293, 1204, 1147, 1065, 1014, 833.

The analytical data are consistent with those reported in the literature [5].

*1.10 Methyl 2-(3,5-dimethoxy-2-(4-methoxybenzoyl)phenyl)acetate (**21**)*

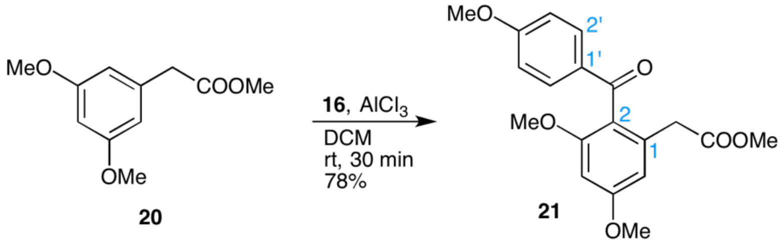

Under an argon atmosphere, a solution of 4-methoxybenzoyl chloride (**16**, 7.85 g, 46 mmol, 1.00 eq) and $AlCl_3$ (6.94 g, 52.0 mmol, 1.13 eq) in DCM (40 mL) was slowly added to a solution of methyl 2-(3,5-dimethoxyphenyl)acetate (**20**, 9.68 g, 46.04 mmol, 1.00 eq) in DCM (40 mL). The mixture was stirred at room temperature for 30 min and carefully quenched by the addition of ice water (200 mL). The organic layer was separated and the aqueous phase was extracted with DCM (2 x 50 mL). The combined organic layers were washed with water (100 mL) and a saturated solution of $NaHCO_3$ (100 mL), dried over Na_2SO_4 and the solvent was removed under reduced pressure. After flash-chromatographic purification on silica (^{c}Hex/EtOAc 3:1), the title compound was obtained as a colorless solid (12.3 g, 35.8 mmol, 78%) alongside a fraction containing the para-substituted phenylacetic acid (**21a**, 0.33 mmol, 3%).

Mp.: 82.3–82.7 °C ($CHCl_3$).

R$_f$: 0.56 (SiO_2, ^{c}Hex/EtOAc 1:1).

^{1}H-NMR, COSY (300.1 MHz, $CDCl_3$): δ/ppm = 7.81–7.74 (m, 2H, *H*-2', *H*-6'), 6.92–6.85 (m, 2H, *H*-3', *H*-5'), 6.49 (d, 1H, *J* = 2.2 Hz, *H*-6), 6.44 (d, 1H, *J* = 2.2 Hz, *H*-4), 3.85 (s, 6H, 5-OC*H*$_3$, 4'-OC*H*$_3$), 3.64 (s, 3H, 3-OC*H*$_3$), 3.53 (s, 2H, C*H*$_2$), 3.46 (s, 3H, COOC*H*$_3$).

^{13}C-NMR, HSQC, HMBC (75.5 MHz, $CDCl_3$): δ/ppm = 195.7 (Ar-CO-Ar), 171.4 (*C*OOCH$_3$), 163.7 (*C*$_q$-4'), 161.4 (*C*$_q$-5), 158.5 (*C*$_q$-3), 134.3 (*C*$_q$-1), 132.0 (2C, *C*-2', *C*-6'), 131.6 (*C*$_q$-1'), 122.6 (*C*$_q$-2), 113.6 (2C, *C*-3', *C*-5'), 107.3 (*C*-6), 97.7 (*C*-4), 55.8 (3-OCH$_3$), 55.6 (2C, 4'-OCH$_3$, 5-OCH$_3$), 52.0 (COOCH$_3$), 38.6 (CH$_2$).

IR: $\bar{v}$[cm^{-1}] = 3004, 2950, 2841, 1736, 1652, 1596, 1315, 1254, 1202, 1157, 1084, 845.

ESI-MS: m/z = 345.1 (100%, $[M+H]^+$, calc. 345.1).

ESI-HRMS: calc. for $[C_{19}H_{20}O_6 + Na]^+$: m/z = 367.1152, found: m/z = 367.1153.

*Isolated Side Product: Methyl 2-(3,5-dimethoxy-4-(4-methoxybenzoyl)phenyl)acetate (**21a**)*

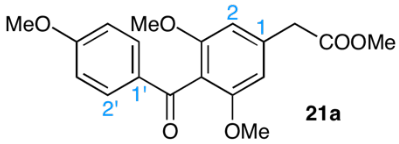

21a

Mp.: 133.1–134.0 °C ($CHCl_3$).

R_f: 0.46 (SiO_2, ^{c}Hex/EtOAc 1:1).

^{1}H-NMR, COSY (300.1 MHz, $CDCl_3$): δ/ppm = 7.86–7.79 (m, 2H, *H*-2', *H*-6'), 6.94–6.86 (m, 2H, *H*-3', *H*-5'), 6.54 (s, 2H, *H*-2, *H*-6), 3.85 (s, 3H, 4'-OCH_3), 3.74 (s, 3H, COOCH_3) 3.70 (s, 6H, 3-OCH_3, 5- OCH_3), 3.64 (s, 2H, CH_2).

^{13}C-NMR, HSQC, HMBC (75.5 MHz, $CDCl_3$): δ/ppm = 193.8 (Ar-CO-Ar), 171.8 (*C*OOCH$_3$), 163.8 (C_q-4'), 157.6 (2C, C_q-3, C_q-5), 136.9 (C_q-1), 132.0 (2C, *C*-2', *C*-6'), 131.0 (C_q-1'), 117.2 (C_q-4), 113.8 (2C, *C*-3', *C*-5'), 105.3 (2C, *C*-2, *C*-6), 56.1 (2C, 3-OCH$_3$, 5-OCH$_3$) 55.6 (4'-OCH$_3$), 52.4 (COOCH$_3$), 41.9 (CH$_2$).

IR: $\bar{\nu}$[cm^{-1}] = 3005, 2951, 2841, 1735, 1662, 1598, 1579, 1418, 1253, 1122, 1025, 925.

ESI-MS: m/z = 345.1 (100%, $[M+H]^+$, calc. 345.1).

ESI-HRMS: calc. for $[C_{19}H_{20}O_6 + H]^+$: m/z = 345.1333, found: m/z = 345.1331.

*1.11 Methyl 2-(3,5-dimethoxy-2-(4-methoxybenzyl)phenyl)acetate (**22**)*

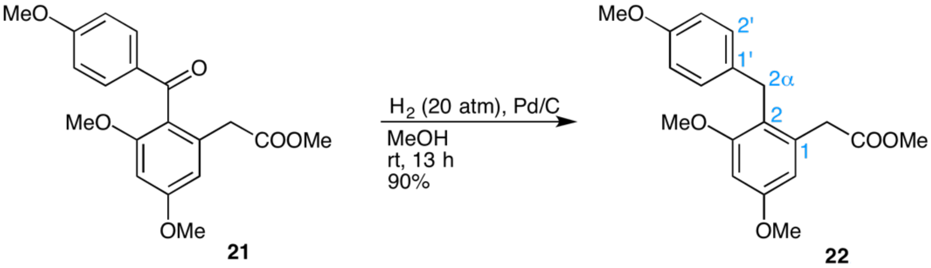

21 **22**

A solution of methyl 2-(3,5-dimethoxy-2-(4-methoxybenzoyl)phenyl)acetate (**21**, 2.14 g, 6.21 mmol, 1.00 eq) and Pd/C (10 wt-% Pd, 214 mg) in MeOH (30 mL) was stirred at room temperature for 20 h under a hydrogen atmosphere (20 atm, autoclave). After filtration over a short plug of silica and concentration under reduced pressure, the title compound was obtained as a colorless solid (1.85 g, 5.61 mmol, 90%).

Mp.: 72.2–72.9 °C ($CHCl_3$).

R_f: 0.58 (SiO_2, ^{c}Hex/EtOAc 2:1).

^{1}H-NMR, COSY (300.1 MHz, $CDCl_3$): δ/ppm = 7.02–6.95 (m, 2H, *H*-2', *H*-6'), 6.80–6.73 (m, 2H, *H*-3', *H*-5'), 6.46 (d, 1H, *J* = 2.5 Hz, *H*-4), 6.41 (d, 1H, *J* = 2.5 Hz, *H*-6), 3.96 (s, 2H, 2⊚-CH_2), 3.81 (s, 3H, 5-OCH_3), 3.78 (s, 3H, 3-OCH_3), 3.75 (s, 3H, 4'-OCH_3), 3.58 (s, 3H, COOCH_3), 3.55 (s, 2H, CH_2COOCH$_3$).

^{13}C-NMR, HSQC, HMBC (75.5 MHz, $CDCl_3$): δ/ppm = 172.0 (*C*OOCH$_3$), 159.0 (C_q-5), 158.9 (C_q-3), 157.7 (C_q-4'), 135.0 (C_q-1), 133.0 (C_q-1'), 129.0 (2C, *C*-2', *C*-6'), 120.9 (C_q-2), 113.7 (2C, *C*-3', *C*-5'), 107.0 (*C*-6), 97.8 (*C*-4), 55.8 (3-OCH$_3$), 55.4 (5-OCH$_3$), 55.3 (4'- OCH$_3$), 52.1 (COOCH$_3$), 39.1 (CH$_2$COOCH$_3$), 30.1 (2⊚-CH$_2$).

IR: $\bar{\nu}$[cm^{-1}] = 2999, 2950, 2836, 1734, 1605, 1509, 1459, 1243, 1202, 1143, 1080, 833.

ESI-MS: m/z = 223.1 (100%, $[M - MeOPh]^+$, calc. 223.1) and m/z = 353.1 (51%, $[M+Na]^+$, calc. 353.1).

ESI-HRMS: calc. for $[C_{19}H_{22}O_5 + Na]^+$: m/z = 353.1359, found: m/z = 353.1359.

Under identical conditions, multigram quantities of **22** (9.71 g, 29.4 mmol) could be obtained in 85% yield starting from **21** (11.9 g, 34.6 mmol) by using a mixture of MeOH (40 mL) and EtOAc (10 mL) as the solvent.

*1.12 2-(3,5-Dimethoxy-2-(4-methoxybenzyl)phenyl)acetic acid (**12**)*

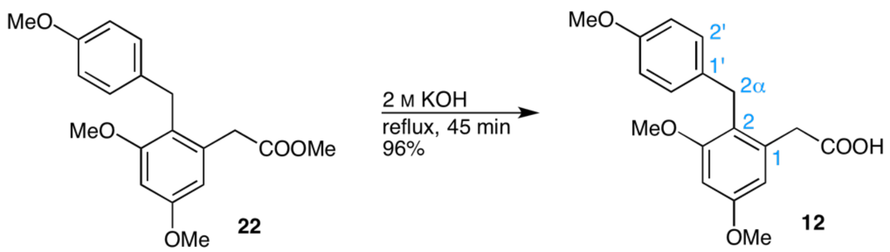

A solution of methyl 2-(3,5-dimethoxy-2-(4-methoxybenzyl)phenyl)acetate (**22**, 3.71 g, 11.2 mmol, 1.00 eq) in 2 M KOH_{aq} (16 mL) was refluxed for 45 min. After cooling to room temperature, the solution was acidified to pH 1 by the dropwise addition of HCl_{aq} (*c* = 2 mol/L) and extracted with EtOAc (3 x 20 mL). The combined organic layers were washed with a saturated solution of NaCl (30 mL), dried over Na_2SO_4 and the solvent was removed under reduced pressure. After washing the residue with hexane (5 x 30 mL), the title compound was obtained as a colorless solid (3.40 g, 4.65 mmol, 96%).

Mp.: 113.6–114.9 °C ($CHCl_3$).

R$_f$: 0.27 (SiO_2, ^{c}Hex/EtOAc 2:1 + 1% AcOH).

^{1}H-NMR, COSY (300.1 MHz, $CDCl_3$): δ/ppm = 7.03–6.95 (m, 2H, *H*-2', *H*-6'), 6.80–6.72 (m, 2H, *H*-3', *H*-5'), 6.47 (d, 1H, *J* = 2.5 Hz, *H*-4), 6.41 (d, 1H, *J* = 2.5 Hz, *H*-6), 3.96 (s, 2H, 2α-C*H*$_2$), 3.81 (s, 3H, 5-OC*H*$_3$), 3.79 (s, 3H, 3-OC*H*$_3$), 3.74 (s, 3H, 4'-OC*H*$_3$), 3.56 (s, 3H, COOC*H*$_3$), 3.55 (s, 2H, C*H*$_2$COOH).

^{13}C-NMR, HSQC, HMBC (75.5 MHz, $CDCl_3$): δ/ppm = 177.6 (COOH), 159.1 (C$_q$-5), 159.0 (C$_q$-3), 157.8 (C$_q$-4'), 134.3 (C$_q$-1), 132.9 (C$_q$-1'), 129.0 (2C, *C*-2', *C*-6'), 121.1 (C$_q$-2), 113.8 (2C, *C*-3', *C*-5'), 107.1 (*C*-6), 98.0 (*C*-4), 55.8 (3-OCH$_3$), 55.4 (5-OCH$_3$), 55.3 (4'- OCH$_3$), 38.8 (CH$_2$COOH), 30.1 (2α-CH$_2$).

IR: $\bar{\nu}$ [cm^{-1}] = 2999, 2937, 2836, 1706, 1605, 1509, 1460, 1243, 1204, 1174, 1143, 732.

ESI-MS: m/z = 339.1 (100%, [M+Na]$^+$, calc. 339.1).

ESI-HRMS: calc. for [$C_{18}H_{20}O_5$ + Na]$^+$: m/z = 339.1203, found: m/z = 339.1202.

*1.13 (E)-2-(3,5-dimethoxy-2-(4-methoxybenzyl)phenyl)-3-(3-methoxyphenyl)prop-2-enoic acid (**23**)*

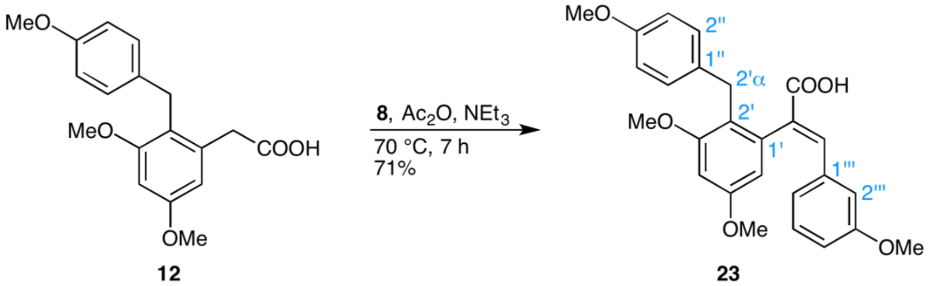

This compound was synthesized using a modified procedure by Wu [15]. A solution of 2-(3,5-dimethoxy-2-(4-methoxybenzyl)phenyl)acetic acid (**12**, 1.34 g, 4.24 mmol, 1.00 eq) and 3-methoxybenzaldehyde (**8**, 510 µL, 4.24 mmol, 1.00 eq) in Ac_2O (600 µL, 6.35 mmol, 1.50 eq) was stirred at 60 °C for 15 min. Then, NEt_3 (1.17 mL, 8.47 mmol, 2.00 eq) was added and the mixture was stirred at 70 °C for 7 h. After cooling to room temperature, the reaction was quenched by the addition of water (30 mL), acidified to pH 7 by the dropwise addition of HCl_{aq} (*c* = 2 mol/L) and extracted with EtOAc (3 x 30 mL). The combined organic layers were washed with water (40 mL) and a saturated solution of NaCl (60 mL), dried over Na_2SO_4 and the solvent was removed under reduced pressure. After flash-chromatographic purification on silica (^{c}Hex/EtOAc 3:1), the title compound was obtained as a colorless solid (1.30 g, 2.99 mmol, 71%).

Mp.: 105.4–109.1 °C ($CHCl_3$).

R$_f$: 0.24 (SiO_2, ^{c}Hex/EtOAc 2:1 + 1% AcOH).

^{1}H-NMR, COSY (600.1 MHz, $CDCl_3$): δ/ppm = 7.84 (s, 1H, *H*-3), 7.08 (t, 1H, *J* = 8.0 Hz, *H*-5'''), 6.96–6.92 (m, 2H, *H*-2'', *H*-6''), 6.77 (dd, 1H, *J* = 8.0 Hz, *J* = 2.2 Hz, *H*-4'''), 6.72 (d, 1H, *J* = 8.0 Hz, *H*-6'''), 6.58–6.55 (m, 2H, *H*-3'', *H*-5''), 6.54 (d, 1H, *J* = 2.4 Hz, *H*-4'), 6.49–6.47 (m, 1H, *H*-2'''), 6.34 (d, 1H, *J* = 2.4 Hz, *H*-6'), 3.83 (s, 3H, 3'-OC*H*$_3$), 3.76 (s, 3H, 5'-OC*H*$_3$), 3.72 (s, 2H, 2'*α*-C*H*$_2$), 3.63 (s, 3H, 4''-OC*H*$_3$), 3.44 (s, 3H, 3'''-OC*H*$_3$).

^{13}C-NMR, HSQC, HMBC (150.9 MHz, $CDCl_3$): δ/ppm = 172.3 (COOH), 159.6 (C$_q$-5'), 159.2 (C$_q$-3'), 159.1 (C$_q$-3'''), 157.4 (C$_q$-4''), 142.6 (*C*-3), 137.2 (C$_q$-1'), 135.4 (C$_q$-1'''), 133.0 (C$_q$-1''), 130.6 (C$_q$-2), 129.7 (2C, *C*-2'', *C*-6''), 129.3 (*C*-5'''), 124.3

(*C*-6'''), 121.4 (C_q-2'), 117.1 (*C*-4'''), 114.1 (*C*-2'''), 113.3 (2C, *C*-3'', *C*-5''), 105.6 (*C*-6'), 99.1 (*C*-4'), 55.8 (3'-OCH_3), 55.5 (5'-OCH_3), 55.2 (4''-OCH_3), 54.9 (3'''-OCH_3), 31.5 (2*α*-CH_2).

IR: $\bar{\nu}$[cm^{-1}] = 3031, 2999, 2931, 2836, 1680, 1600, 1510, 1245, 1201, 1145, 1038, 732.

ESI-MS: m/z = 327.2 (100%, [M – MeOPh]$^+$, calc. 327.1) and m/z = 457.2 (52%, [M+Na]$^+$, calc. 457.2).

ESI-HRMS: calc. for [$C_{26}H_{25}O_6$]$^-$: m/z = 433.1657, found: m/z = 433.1650.

*1.14 1,5-Dimethoxy-2-(4-methoxybenzyl)-3-((E/Z)-2-(3-methoxyphenyl)ethenyl)benzene (**24**)*

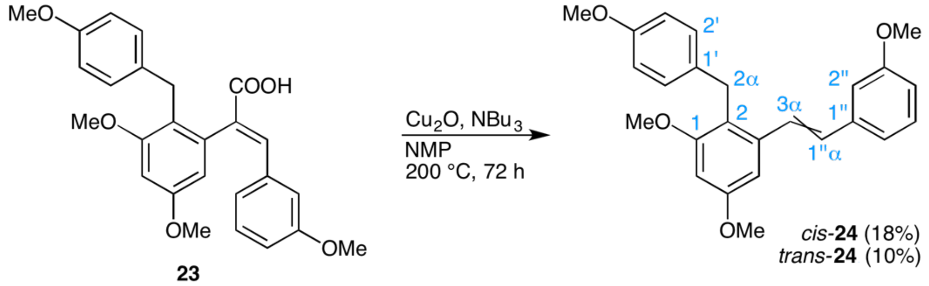

23

This compound was synthesized using a modified procedure by Wu [15]. To a suspension of (*E*)-2-(3,5-dimethoxy-2-(4-methoxybenzyl)phenyl)-3-(3-methoxyphenyl)prop-2-enoic acid (**23**, 1.05 g, 2.42 mmol, 1.00 eq) and Cu_2O (17 mg, 0.12 mmol, 0.05 eq) in NMP (4.0 mL) was added NBu_3 (290 ◎L, 1.21 mmol, 0.50 eq). The mixture was stirred at 200 °C for 72 h and, after cooling to room temperature, quenched by the addition of HCl_{aq} (*c* = 2 mol/L, 25 mL). A saturated solution of NaCl (25 mL) and EtOAc (25 mL) was added and the suspension was filtered. The organic layer was separated, washed with a saturated solution of NaCl (3 x 50 mL), dried over Na_2SO_4 and the solvent was removed under reduced pressure. After flash-chromatographic purification on silica (^{c}Hex/EtOAc 20:1), the title compound was obtained as two separated *cis*/*trans* isomers (*cis*-**24**: light-yellow oil, 168 mg, 0.43 mmol, 18% and *trans*-**24**: light-yellow oil, 94.2 mg, 0.24 mmol, 10%).

cis-**24**:

R$_f$: 0.17 (SiO_2, ^{c}Hex/EtOAc 20:1).

^{1}H-NMR, COSY (300.1 MHz, $CDCl_3$): δ/ppm = 7.15–7.05 (m, 3H, *H*-2', *H*-6', *H*-5''), 6.79–6.73 (m, 3H, *H*-3', *H*-5', *H*-6''), 6.72–6.68 (m, 1H, *H*-4''), 6.67–6.66 (m, 1H, *H*- 2''), 6.65 (d, 1H, *J* = 12.3 Hz, 3*α*-C*H*), 6.56 (d, 1H, *J* = 12.3 Hz, 1''*α*-C*H*), 6.42 (d, 1H, *J* = 2.4 Hz, *H*-6), 6.33 (d, 1H, *J* = 2.4 Hz, *H*-4), 3.95 (s, 2H, 2*α*-C*H*$_2$), 3.80 (s, 3H, 1-OC*H*$_3$), 3.75 (s, 3H, 4'-OC*H*$_3$), 3.60 (s, 3H, 5-OC*H*$_3$), 3.57 (s, 3H, 3''-OC*H*$_3$).

^{13}C-NMR, HSQC, HMBC (75.5 MHz, $CDCl_3$): δ/ppm = 159.2 (C_q-3''), 158.9 (C_q-1), 158.8 (C_q-5), 157.6 (C_q-4'), 139.0 (C_q-3), 138.2 (C_q-1''), 133.5 (C_q-1'), 130.8 (1''*α*-CH), 130.0 (3*α*-CH), 129.3 (2C, *C*-2', *C*-6'), 129.1 (*C*-5''), 121.9 (*C*-6''), 120.7 (C_q-2), 113.8 (*C*-2''), 113.7 (*C*-4''), 113.6 (2C, *C*-3', *C*-5'), 105.1 (*C*-4), 98.2 (*C*-6), 55.7 (1-OCH_3), 55.4 (5-OCH_3), 55.3 (4'-OCH_3), 55.0 (3''-OCH_3), 31.0 (2*α*-CH_2).

IR: $\bar{\nu}$[cm^{-1}] = 3000, 2934, 2834, 1580, 1509, 1460, 1243, 1201, 1143, 1037, 793, 690.

ESI-MS: m/z = 391.3 (100%, [M+H]$^+$, calc. 391.2).

ESI-HRMS: calc. for [$C_{25}H_{26}O_4$ + H]$^+$: m/z = 391.1904, found: m/z = 391.1903.

trans-**24**:

R$_f$: 0.15 (SiO_2, ^{c}Hex/EtOAc 20:1).

^{1}H-NMR, COSY (300.1 MHz, $CDCl_3$): δ/ppm = 7.36 (d, 1H, *J* = 16.1 Hz, 3*α*-C*H*), 7.26 (t, 1H, *J* = 7.8 Hz, *H*-5''), 7.14–7.07 (m, 2H, *H*-2', *H*-6'), 7.04 (d, 1H, *J* = 7.8 Hz, *H*-6''), 6.97–6.95 (m, 1H, *H*-2''), 6.95 (d, 1H, *J* = 16.1 Hz, 1''*α*-C*H*), 6.85–6.80 (m, 3H, *H*-3', *H*-5', *H*-4''), 6.78 (d, 1H, *J* = 2.4 Hz, *H*-4), 6.48 (d, 1H, *J* = 2.4 Hz, H-6), 4.07 (s, 2H, 2*α*-C*H*$_2$), 3.88 (s, 3H, 5-OC*H*$_3$), 3.83 (s, 3H, 3''-OC*H*$_3$), 3.81 (s, 3H, 1-OC*H*$_3$), 3.76 (s, 3H, 4'-OC*H*$_3$).

^{13}C-NMR, HSQC, HMBC (75.5 MHz, $CDCl_3$): δ/ppm = 159.9 (C_q-3''), 159.1 (C_q-5), 158.9 (C_q-1), 157.7 (C_q-4'), 139.0 (C_q-1''), 138.2 (C_q-3), 133.7 (C_q-1'), 130.6 (1''*α*-CH), 129.7 (*C*-5''), 129.2 (2C, *C*-2', *C*-6'), 127.4 (3*α*-CH), 120.6 (C_q-2), 119.4 (*C*-6''), 113.8 (2C, *C*-3', *C*-5'), 113.4 (*C*-4''), 111.9 (*C*-2''), 101.8 (*C*-4), 98.4 (*C*-6), 55.9 (1-OCH_3), 55.5 (5-OCH_3), 55.3 (3''-OCH_3, 4'-OCH_3), 30.1 (2*α*-CH_2).

IR: $\bar{\nu}$[cm^{-1}] = 2999, 2935, 2834, 1579, 1509, 1456, 1243, 1201, 1143, 1036, 829, 776.

ESI-MS: m/z = 391.3 (100%, [M+H]$^+$, calc. 391.2).

ESI-HRMS: calc. for [$C_{25}H_{26}O_4$ + H]$^+$: m/z = 391.1904, found: m/z = 391.1903.

*1.15 1,5-Dimethoxy-2-(4-methoxybenzyl)-3-(2-(3-methoxyphenyl)ethyl)benzene (**9**)*

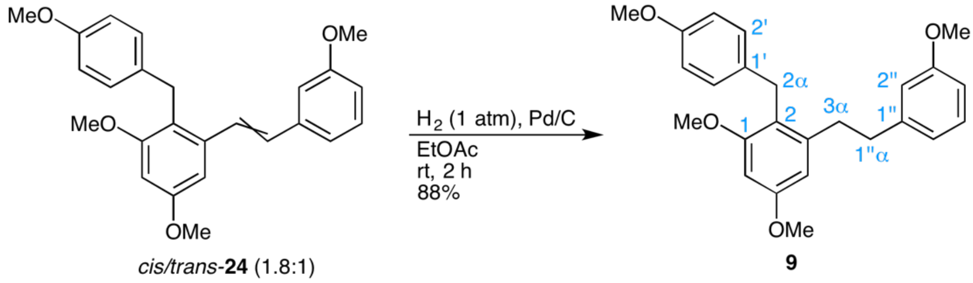

A solution of 1,5-dimethoxy-2-(4-methoxybenzyl)-3-((*E*/*Z*)-2-(3-methoxyphenyl)ethenyl)benzene (cis/trans-**24**, 230 mg, 0.59 mmol, 1.00 eq) and Pd/C (10 wt-% Pd, 12 mg) in EtOAc (4 mL) was stirred at room temperature for 2 h under a hydrogen atmosphere (1 atm). After filtration over a short plug of silica and concentration under reduced pressure, the title compound was obtained as a light-yellow oil (204 mg, 0.53 mmol, 88%).

R$_f$: 0.39 (SiO_2, ^{c}Hex/EtOAc 9:1).

^{1}H-NMR, COSY (300.1 MHz, $CDCl_3$): δ/ppm = 7.18 (t, 1H, *J* = 8.0 Hz, *H*-5''), 7.06–6.99 (m, 2H, *H*-2', *H*-6'), 6.81–6.76 (m, 2H, *H*-3', *H*-5'), 6.75–6.69 (m, 2H, *H*-4'', *H*-6''), 6.65–6.62 (m, 1H, *H*-2''), 6.41 (d, 1H, *J* = 2.5 Hz, *H*-6), 6.35 (d, 1H, *J* = 2.5 Hz, *H*-4), 3.96 (s, 2H, 2*α*-C*H*$_2$), 3.80 (s, 3H, 5-OC*H*$_3$), 3.79 (s, 3H, 1-OC*H*$_3$), 3.78 (s, 3H, 3''-OC*H*$_3$), 3.76 (s, 3H, 4'-OC*H*$_3$), 2.88–2.80 (m, 2H, 3*α*-C*H*$_2$), 2.73–2.64 (m, 2H, 1''*α*-C*H*$_2$).

^{13}C-NMR, HSQC, HMBC (75.5 MHz, $CDCl_3$): δ/ppm = 159.7 (*C*$_q$-3''), 159.0 (2C, *C*$_q$-1, *C*$_q$-5), 157.6 (*C*$_q$-4'), 143.7 (*C*$_q$-1''), 142.5 (*C*$_q$-3), 133.9 (*C*$_q$-1''), 129.4 (*C*-5''), 129.0 (2C, *C*-2', *C*-6'), 120.9 (*C*-6''), 120.0 (*C*$_q$-2), 114.2 (*C*-2''), 113.7 (2C, *C*-3', *C*-5'), 111.4 (*C*-4''), 105.9 (*C*-4), 96.6 (*C*-6), 55.8 (1-OCH$_3$), 55.4 (2C, 5-OCH$_3$, 3''-OCH$_3$), 55.3 (4'-OCH$_3$), 37.7 (1''*α*-CH$_2$), 35.6 (3*α*-CH$_2$), 30.1 (2*α*-CH$_2$).

IR: $\bar{\nu}$ [cm^{-1}] = 2997, 2936, 2834, 1583, 1509, 1455, 1243, 1142, 1080, 1037, 830, 696.

ESI-MS: m/z = 285.2 (100%, [M – MeOPh]$^+$, calc. 285.1) and m/z = 393.2 (80%, [M+H]$^+$, calc. 393.2).

ESI-HRMS: calc. for [$C_{25}H_{28}O_4$ + H]$^+$: m/z = 393.2060, found: m/z = 392.2062.

*1.16 4-(4-Hydroxybenzyl)-5-(2-(3-hydroxyphenyl)ethyl)benzene-1,3-diol (**1**, shancigusin C)*

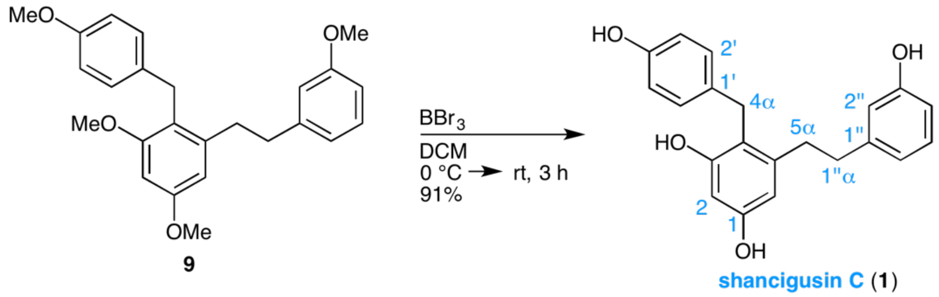

A solution of 1,5-dimethoxy-2-(4-methoxybenzyl)-3-(2-(3-methoxyphenyl)ethyl)benzene (**9**, 175 mg, 0.45 mmol, 1.00 eq) in anhydrous DCM (3 mL) was cooled to 0 °C, followed by the dropwise addition of BBr_3 (1 M in DCM, 1.96 mL, 1.96 mmol, 4.40 eq). The mixture was allowed to reach room temperature, stirred for 3 h and quenched by the addition of ice water (30 mL). DCM was removed under reduced pressure and the residual aqueous phase was extracted with EtOAc (2 x 15 mL). The combined organic layers were washed with water (10 mL) and brine (10 mL), dried over Na_2SO_4 and the solvent was removed under reduced pressure. After recrystallization from MeOH/H_2O (1:1), the title compound was obtained as colorless crystals (136 mg, 0.40 mmol, 91%).

Mp.: 175.8–176.1 °C (MeOH/H_2O 1:1).

R$_f$: 0.32 (SiO_2, ^{c}Hex/EtOAc 1:1 + 1% AcOH).

^{1}H-NMR, COSY (300.1 MHz, methanol-d$_4$): δ/ppm = 7.03 (t, 1H, *J* = 7.8 Hz, *H*-5''), 6.97–6.89 (m, 2H, *H*-2', *H*-6'), 6.67–6.61 (m, 2H, *H*-3', *H*-5'), 6.60–6.51 (m, 3H, *H*-2'', *H*-4'', *H*-6''), 6.23 (d, 1H, *J* = 2.2 Hz, *H*-2), 6.19 (d, 1H, *J* = 2.2 Hz, *H*-6), 3.84 (s, 2H, 4*α*-C*H*$_2$), 2.73–2.61 (m, 2H, 5*α*-C*H*$_2$), 2.60–2.49 (m, 2H, 1''*α*-C*H*$_2$).

^{13}C-NMR, HSQC, HMBC (75.5 MHz, methanol-d$_4$): δ/ppm = 158.3 (*C*$_q$-3''), 157.5 (*C*$_q$-3), 157.0 (*C*$_q$-1), 155.9 (*C*$_q$-4'), 145.0 (*C*$_q$-1''), 144.0 (*C*$_q$-5), 134.4 (*C*$_q$-1'), 130.2 (*C*-5''), 130.1 (2C, *C*-2', *C*-6') 120.7 (*C*-6''), 118.6 (*C*$_q$-4), 116.2 (*C*-2''), 115.8 (2C, *C*-3', *C*-5'), 113.7 (*C*-4''), 108.7 (*C*-6), 101.3 (*C*-2), 38.6 (1''*α*-CH$_2$), 36.6 (5*α*-CH$_2$), 30.7 (4*α*-CH$_2$).

IR: $\bar{\nu}$[cm^{-1}] = 3329, 2937, 2864, 1610, 1595, 1510, 1456, 1237, 1136, 837, 785, 697.
ESI-MS: m/z = 337.3 (100%, [M+H]$^+$, calc. 337.1).
APPI-HRMS: calc. for $[C_{21}H_{20}O_4]^{\cdot +}$: m/z = 336.1356, found: m/z = 336.1346.

*1.17 4-Bromo-3,5-dihydroxybenzoic acid (**30**)*

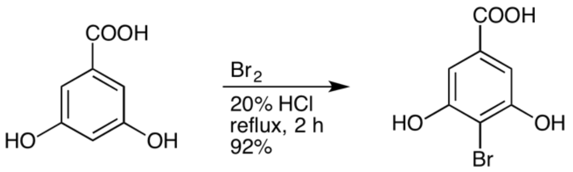

29 **30**

This compound was prepared according to a procedure by Sauvage [16].
The title compound was obtained as a slightly brownish solid (7.14 g, 30.66 mmol, 92%).
Mp.: 258.4–262.1 °C (Et_2O), Lit.: 245–250 °C (Et_2O) [17].
R$_f$: 0.22 (SiO_2, EtOAc).
^{1}H-NMR, COSY (300.1 MHz, $(CD_3)_2CO$): δ/ppm = 9.12 (s, 2H, 2 x O*H*), 7.20 (s, 2H, *H*-2, *H*-6).
^{13}C-NMR, HSQC, HMBC (75.5 MHz, $(CD_3)_2CO$): δ/ppm = 165.2 (*C*OOH), 154.4 (2C, *C*$_q$-3, *C*$_q$-5), 129.7 (*C*$_q$-1), 107.1 (2C, *C*-2, *C*-6), 102.5 (*C*$_q$-4).
IR: $\bar{\nu}$[cm^{-1}] = 3573, 3480, 3070, 1692, 1592, 1513, 1437, 1351, 1270, 1188, 1052, 768.
ESI-MS: m/z = 232.9 (100%, [M – H]$^-$, calc. 232.9).
The analytical data are consistent with those reported in the literature [22, 23].

*1.18 Methyl 4-bromo-3,5-dimethoxybenzoate (**31**)*

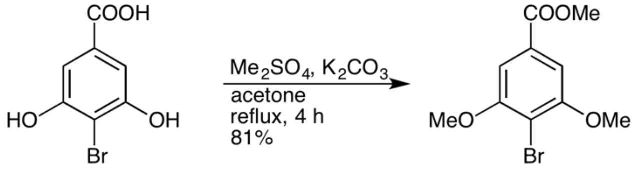

30 **31**

This compound was prepared according to a procedure by Wilcox [18].
The title compound was obtained as colorless crystals (6.67 g, 24.25 mmol, 81%).
Mp.: 120.1–120.3 °C (MeOH), Lit.: 121–122 °C (MeOH) [18].
R$_f$: 0.38 (SiO_2, ^{c}Hex/EtOAc 4:1).
^{1}H-NMR, COSY (300.1 MHz, $CDCl_3$): δ/ppm = 7.24 (s, 2H, *H*-2, *H*-6), 3.95 (s, 6H, 2 x OC*H*$_3$), 3.93 (s, 3H, COOC*H*$_3$).
^{13}C-NMR, HSQC, HMBC (75.5 MHz, $CDCl_3$): δ/ppm = 166.5 (*C*OOCH$_3$), 157.0 (2C, *C*$_q$-3, *C*$_q$-5), 130.2 (*C*$_q$-1), 106.6 (*C*$_q$-4), 105.5 (2C, *C*-2, *C*-6), 56.6 (2C, 2 x OCH$_3$) 52.5 (COOCH$_3$).
IR: $\bar{\nu}$[cm^{-1}] = 3002, 2956, 2839, 1717, 1587, 1459, 1407, 1332, 1242, 1119, 1003, 760.
ESI-MS: m/z = 276.9 (100%, [M+H]$^+$, calc. 277.0).
The analytical data are consistent with those reported in the literature [18].

*1.19 2-Bromo-5-(bromomethyl)-1,3-dimethoxybenzene (**33**)*

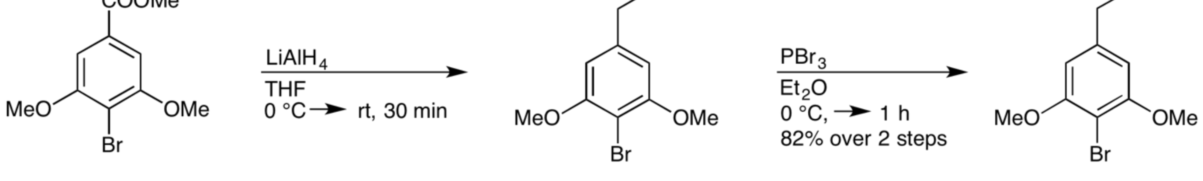

31 **32** **33**

Part A. A solution of methyl 4-bromo-3,5-dimethoxybenzoate (**31**, 1.33 g, 4.83 mmol, 1.00 eq) in anhydrous THF (20 mL) was added dropwise to a suspension of $LiAlH_4$ (1.10 g, 28.99 mmol, 6.00 eq) in anhydrous THF (30 mL) at 0 °C. The mixture was stirred for 30 min at room temperature and quenched by the addition of a saturated solution of NH_4Cl (50

mL). The solution was acidified to pH 6 by the dropwise addition of HCl_{aq} (c = 1 mol/L) and extracted with EtOAc (3 x 30 mL). The combined organic layers were dried over Na_2SO_4 and the solvent was removed under reduced pressure.

Part B. The crude benzyl alcohol (**32**, assuming 4.83 mmol) was dissolved in dry Et_2O (20 mL) and the solution was cooled to 0 °C. PBr_3 (0.23 mL, 2.42 mmol, 0.50 eq) was added dropwise at 0 °C and the mixture was stirred for 1 h at room temperature. The reaction was quenched by the addition of a precooled saturated solution of $NaHCO_3$ (40 mL) and extracted with EtOAc (3 x 20 mL). The combined organic layers were washed with brine (30 mL) and a saturated solution of $NaHCO_3$ (30 mL), dried over Na_2SO_4 and the solvent was removed under reduced pressure to obtain the title compound as a colorless oil (1.23 g, 3.95 mmol, 82%).

R$_f$: 0.24 (SiO_2, ^{c}Hex/EtOAc 30:1).

^{1}H-NMR, COSY (300.1 MHz, $CDCl_3$): δ/ppm = 6.60 (s, 2H, *H*-4, *H*-6), 4.45 (s, 2H, C*H*$_2$), 3.91 (s, 6H, 2 x OC*H*$_3$).

^{13}C-NMR, HSQC, HMBC (75.5 MHz, $CDCl_3$): δ/ppm = 157.3 (2C, *C*$_q$-1, *C*$_q$-3), 138.4 (*C*$_q$-5), 105.5 (2C, *C*-4, *C*-6), 101.2 (*C*$_q$-2), 56.7 (2C, 2 x O*C*H$_3$), 33.5 (*C*H$_2$).

IR: $\bar{\nu}$ [cm^{-1}] = 3006, 2940, 2839, 1588, 1459, 1414, 1330, 1242, 1130, 1047, 829, 605.

ESI-MS: m/z = 310.9 (100%, [M+H]$^+$, calc. 310.9).

The analytical data are consistent with those reported in the literature [19].

1.20 2-Bromo-1,3-dimethoxy-5-(2-(3-methoxyphenyl)ethyl)benzene (33)

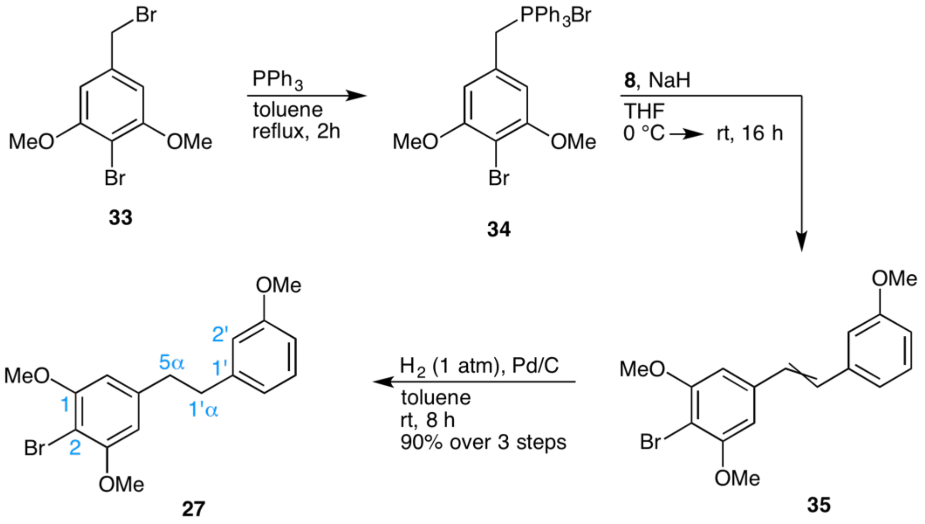

Part A. To a solution of 2-bromo-5-(bromomethyl)-1,3-dimethoxybenzene (**33**, 4.06 g, 13.10 mmol, 1.00 eq) in anhydrous toluene (60 mL) was added PPh_3 (4.46 g, 17.00 mmol, 1.30 eq) in one portion. The mixture was refluxed for 2 h and stirred another 30 min at 0 °C. After filtration, the residue was washed with toluene (5 x 20 mL) and Et_2O (5 x 20 mL).

Part B. The crude phosphonium bromide (**34**, assuming 13.10 mmol) was suspended in anhydrous THF (80 mL) and the solution was cooled to 0 °C. NaH (60% dispersion, 0.58 g, 14.51 mmol, 1.11 eq) was added at 0 °C, followed by the dropwise addition of 3-methoxybenzaldehyde (8, 1.79 g, 13.10 mmol, 1.00 eq). The reaction was stirred for 16 h at room temperature and quenched by the addition of water (100 mL). The mixture was extracted with EtOAc (3 x 50 mL) and the combined organic layers were washed with water (50 mL) and brine (50 mL). The organic layer was dried over Na_2SO_4 and the solvent was removed under reduced pressure.

Part C. The crude *cis/trans* stilbene (**35**, assuming 13.10 mmol) and Pd/C (10 wt-% Pd, 350 mg) were dissolved in dry toluene (80 mL) and stirred at room temperature for 8 h under a hydrogen atmosphere (1 atm). The mixture was filtered over a short plug of silica and the filtrate was concentrated under reduced pressure. After flash-chromatographic purification on silica (^{c}Hex/EtOAc 20:1), the title compound was obtained as a colorless solid (4.13 g, 11.76 mmol, 90%). Note: The use of toluene as solvent is crucial as MeOH, EtOH or EtOAc led to cleavage of the bromine substituent.

Mp.: 63.6–64.3 °C ($CHCl_3$).

R$_f$: 0.23 (SiO_2, ^{c}Hex/EtOAc 20:1).

^{1}H-NMR, COSY (400.1 MHz, $CDCl_3$): δ/ppm = 7.21 (t, 1H, *J* = 7.9 Hz, *H*-5'), 6.79–6.74 (m, 2H, *H*-4', *H*-6'), 6.73–6.71 (m, 1H, *H*-2'), 6.35 (s, 2H, *H*-4, *H*-6), 3.84 (s, 6H, 1-OC*H*$_3$, 3-OC*H*$_3$), 3.78 (s, 3H, 3'-OC*H*$_3$), 2.90 (s, 4H, 5α-C*H*$_2$, 1'α-C*H*$_2$).

^{13}C-NMR, HSQC, HMBC (100.6 MHz, CDCl$_3$): δ/ppm = 159.7 (C_q-3'), 156.9 (2C, C_q-1, C_q-3), 143.0 (C_q-1'), 142.5 (C_q-5), 129.4 (*C*-5'), 121.0 (*C*-6'), 114.4 (*C*-2'), 111.4 (*C*-4'), 105.2 (2C, *C*-4, *C*-6), 98.2 (C_q-2), 56.5 (2C, 1-OCH$_3$, 3-OCH$_3$), 55.3 (3'-OCH$_3$), 38.3 (5*α*-CH$_2$), 37.8 (1'*α*-CH$_2$).

IR: $\tilde{\nu}$ [cm^{-1}] = 3001, 2938, 2837, 1585, 1476, 1456, 1414, 1240, 1121, 1047, 825, 697.

ESI-MS: m/z = 351.0 (100%, [M+H]$^+$, calc. 351.1).

ESI-HRMS: calc. for [$C_{17}H_{19}BrO_3$ + H]$^+$: m/z = 351.0590, found: m/z = 351.0590.

*1.21 (2,6-Dimethoxy-4-(2-(3-methoxyphenyl)ethyl)phenyl)(4-methoxyphenyl)methanol (**36**)*

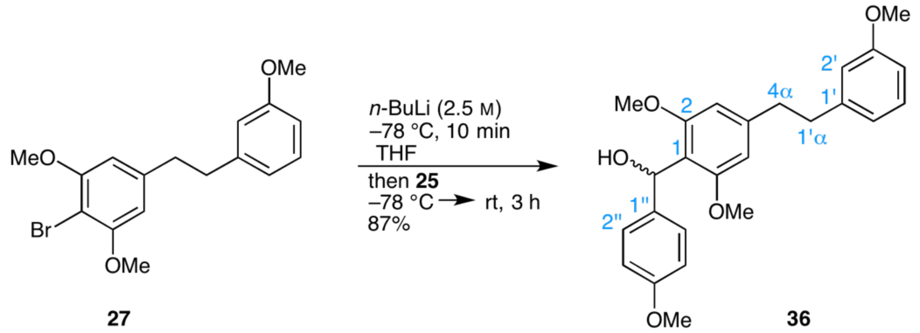

Under an argon atmosphere, a solution of 2-bromo-1,3-dimethoxy-5-(2-(3-methoxy-phenyl)ethyl)benzene (**27**, 3.59 g, 10.22 mmol, 1.00 eq) in anhydrous THF (50 mL) was cooled to –78 °C and *n*-BuLi (2.5 M in hexanes, 4.50 mL, 11.25 mmol, 1.10 eq) was added dropwise. The solution was stirred for 10 min at –78 °C, followed by the dropwise addition of 4-methoxybenzaldehyde (**25**, 1.24 mL, 10.22 mmol, 1.00 eq). The reaction mixture was stirred for 3 h at room temperature, quenched by the addition of a saturated solution of NH_4Cl (50 mL) and extracted with Et_2O (3 x 50 mL). The combined organic layers were dried over Na_2SO_4 and the solvent was removed under reduced pressure. After flash-chromatographic purification on silica (^{c}Hex/EtOAc 5:1), the title compound was obtained as a colorless oil (3.65 g, 8.94 mmol, 87%).

R$_f$: 0.20 (SiO$_2$, ^{c}Hex/EtOAc 5:1).

^{1}H-NMR, COSY (600.1 MHz, CDCl$_3$): δ/ppm = 7.29–7.25 (m, 2H, *H*-2'', *H*-6''), 7.22 (t, 1H, *J* = 7.8 Hz, *H*-5'), 6.84–6.81 (m, 2H, *H*-3'', *H*-5''), 6.80 (dt, 1H, *J* = 7.8 Hz, *J* = 1.3 Hz, *H*-6'), 6.76 (dd, 1H, *J* = 7.8 Hz, *J* = 2.4 Hz, *H*-4'), 6.73–6.71 (*J* = 2.4 Hz, *J* = 1.3 Hz, *H*-2'), 6.39 (s, 2H, *H*-3, *H*-5), 6.23 (d, 1H, *J* = 11.6 Hz, C*H*OH), 4.37 (d, 1H, *J* = 11.6 Hz, CHO*H*), 3.78 (s, 6H, 3'-OC*H*$_3$, 4''-OC*H*$_3$), 3.75 (s, 6H, 2-OC*H*$_3$, 6-OC*H*$_3$), 2.90 (s, 4H, 4*α*-C*H*$_2$, 1'*α*-C*H*$_2$).

^{13}C-NMR, HSQC, HMBC (150.9 MHz, CDCl$_3$): δ/ppm = 159.7 (C_q-3'), 158.3 (C_q-4''), 157.6 (2C, C_q-2, C_q-6), 143.2 (C_q-1'), 142.9 (C_q-4), 137.2 (C_q-1''), 129.4 (*C*-5'), 127.1 (2C, *C*-2'', *C*-6''), 121.0 (*C*-6'), 117.1 (C_q-1), 114.4 (*C*-2'), 113.4 (2C, *C*-3'', *C*-5''), 111.4 (*C*-4'), 104.9 (2C, *C*-3, *C*-5), 68.4 (CHOH), 55.9 (2C, 2-OCH$_3$, 6-OCH$_3$), 55.3 (2C, 3'-OCH$_3$, 4''-OCH$_3$), 38.5 (4*α*-CH$_2$), 38.0 (1'*α*-CH$_2$).

IR: $\tilde{\nu}$ [cm^{-1}] = 3542, 2938, 2836, 1608, 1584, 1457, 1421, 1245, 1222, 1115, 1036, 780.

ESI-MS: m/z = 391.2 (100%, [M – OH]$^+$, calc. 391.2).

ESI-HRMS: calc. for [$C_{25}H_{28}O_5$ + Na]$^+$ m/z = 431.1829, found: m/z = 431.1833.

*1.22 1,3-Dimethoxy-2-(4-methoxybenzyl)-5-(2-(3-methoxyphenyl)ethyl)benzene (**26**)*

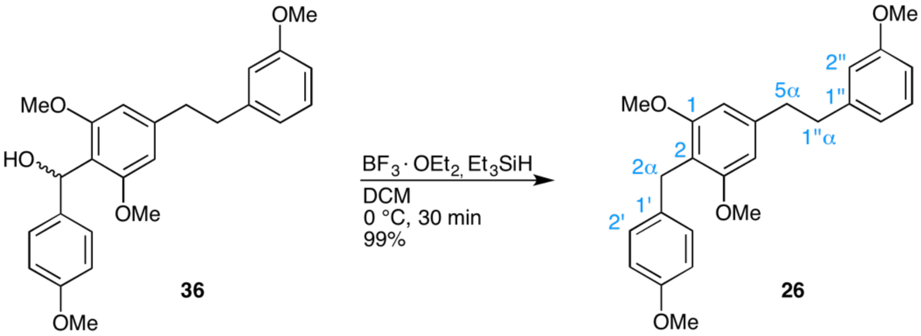

Under an argon atmosphere, a solution of (2,6-dimethoxy-4-(2-(3-methoxyphenyl)ethyl)-phenyl)(4-methoxyphenyl)methanol (**36**, 3.54 g, 8.67 mmol, 1.00 eq) in anhydrous DCM (36 mL) was cooled to 0 °C and Et_3SiH (2.77 mL,

17.32 mmol, 2.00 eq) was added, followed by the dropwise addition of $BF_3{\cdot}OEt_2$ (1.20 mL, 9.52 mmol, 1.10 eq). The solution was stirred for 30 min at 0 °C, quenched by the addition of water (35 mL) and extracted with DCM (3 x 30 mL). The combined organic layers were dried over Na_2SO_4 and the solvent was removed under reduced pressure. After filtration over a short plug of silica (^{c}Hex/EtOAc 10:1), the title compound was obtained as a colorless oil which solidified upon standing (3.37 g, 8.59 mmol, 99%).

Mp.: 62.1–62.4 °C ($CHCl_3$).

R$_f$: 0.48 (SiO_2, ^{c}Hex/EtOAc 5:1).

^{1}H-NMR, COSY (600.1 MHz, $CDCl_3$): δ/ppm = 7.23 (t, 1H, *J* = 7.8 Hz, *H*-5''), 7.22–7.18 (m, 2H, *H*-2', *H*-6'), 6.83 (dt, 1H, *J* = 7.8 Hz, *J* = 1.2 Hz, *H*-6''), 6.80–6.76 (m, 3H, *H*-3', *H*-5', *H*-4''), 6.75–6.73 (m, 1H, *H*-2''), 6.39 (s, 2H, *H*-4, *H*-6), 3.93 (s, 2H, 2α-CH_2), 3.79 (s, 6H, 1-OCH_3, 3-OCH_3), 3.78 (s, 3H, 3''-OCH_3), 3.77 (s, 3H, 4'-OCH_3), 2.94–2.87 (m, 4H, 5α-CH_2, 1''α-CH_2).

^{13}C-NMR, HSQC, HMBC (150.9 MHz, $CDCl_3$): δ/ppm = 159.7 (C_q-3''), 158.1 (2C, C_q-1, C_q-3), 157.5 (C_q-4'), 143.6 (C_q-1''), 141.2 (C_q-5), 134.3 (C_q-1'), 129.5 (2C, *C*-2', *C*-6'), 129.4 (*C*-5''), 121.0 (*C*-6''), 115.8 (C_q-2), 114.3 (*C*-2''), 113.5 (2C, *C*-3', *C*-5'), 111.4 (*C*-4''), 104.2 (2C, *C*-4, *C*-6), 55.8 (2C, 1-OCH_3, 3-OCH_3), 55.3 (2C, 4'-OCH_3, 3''-OCH_3), 38.6 (5α-CH_2), 38.2 (1''α-CH_2), 27.7 (2α-CH_2).

IR: $\bar{\nu}$ [cm^{-1}] = 2997, 2935, 2834, 1603, 1585, 1509, 1454, 1243, 1174, 1116, 1037, 696.

ESI-MS: m/z = 393.2 (100%, [M+H]$^+$, calc. 393.2).

ESI-HRMS: calc. for [$C_{25}H_{28}O_4$ + Na]$^+$: m/z = 415.1880, found: m/z = 415.1881.

*1.23 2-Bromo-3,5-dimethoxy-4-(4-methoxybenzyl)-1-(2-(3-methoxyphenyl)ethyl)benzene (**37**)*

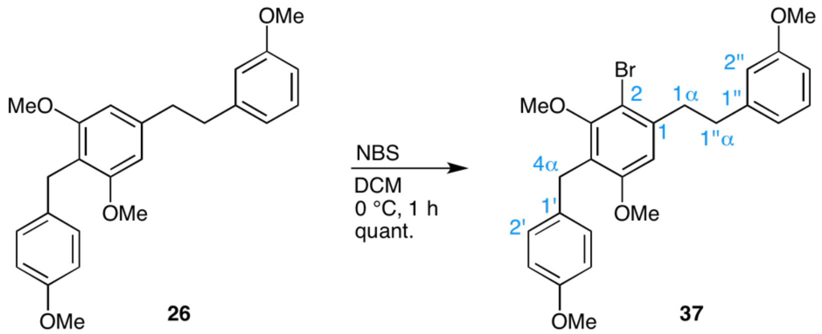

Under an argon atmosphere, a solution of 1,3-dimethoxy-2-(4-methoxybenzyl)-5-(2-(3-methoxyphenyl)-ethyl)benzene (**26**, 2.09 g, 5.32 mmol, 1.00 eq) in anhydrous DCM (40 mL) was cooled to 0 °C and NBS (0.95 g, 5.32 mmol, 1.00 eq) was added in one portion. The mixture was stirred for 1 h at 0 °C, quenched by the addition of a saturated solution of $NaHCO_3$ (50 mL) and extracted with DCM (3 x 30 mL). The combined organic layers were dried over Na_2SO_4 and the solvent was removed under reduced pressure. To remove succinimide, the residue was taken up with ^{c}Hex/EtOAc 10:1 and filtrated over a short plug of silica (^{c}Hex/EtOAc 10:1). After removal of the solvent under reduced pressure, the title compound was obtained as a colorless oil (2.50 g, 5.30 mmol, quant.).

R$_f$: 0.44 (SiO_2, ^{c}Hex/EtOAc 10:1).

^{1}H-NMR, COSY (600.1 MHz, $CDCl_3$): δ/ppm = 7.22 (td, 1H, *J* = 7.9 Hz, *J* = 1.0 Hz, *H*-5''), 7.16–7.12 (m, 2H, *H*-2', *H*-6'), 6.84 (dt, 1H, *J* = 7.9 Hz, *J* = 1.0 Hz, *H*-6''), 6.79–6.77 (m, 2H, *H*-3', *H*-5'), 6.77–6.75 (m, 2H, *H*-2'', *H*-4''), 6.48 (s, 1H, *H*-6), 3.97 (s, 2H, 4α-CH_2), 3.77 (s, 3H, 3''-OCH_3), 3.76 (s, 3H, 4'-OCH_3), 3.72 (s, 6H, 3-OCH_3, 5-OCH_3), 3.05–3.00 (m, 2H, 1α-CH_2), 2.91–2.86 (1''α-CH_2).

^{13}C-NMR, HSQC, HMBC (150.9 MHz, $CDCl_3$): δ/ppm = 159.7 (C_q-3''), 157.7 (C_q-4'), 157.4 (C_q-5), 156.0 (C_q-3), 143.3 (C_q-1''), 140.5 (C_q-1), 133.3 (C_q-1'), 129.5 (*C*-5''), 129.4 (2C, *C*-2', *C*-6'), 123.0 (C_q-4), 121.1 (*C*-6''), 114.4 (*C*-2''), 113.6 (2C, *C*-3', *C*-5'), 111.5 (*C*-4''), 110.8 (C_q-2), 108.9 (*C*-6), 61.3 (3-OCH_3), 55.9 (5-OCH_3), 55.3 (2C, 4'-OCH_3, 3''-OCH_3), 39.1 (1α-CH_2), 36.4 (1''α-CH_2), 29.2 (4α-CH_2).

IR: $\bar{\nu}$ [cm^{-1}] = 2998, 2936, 2834, 1594, 1585, 1510, 1459, 1392, 1246, 1155, 1098, 1041.

ESI-MS: m/z = 471.2 (100%, [M+H]$^+$, calc. 471.1).

ESI-HRMS: calc. for [$C_{25}H_{27}BrO_4$ + H]$^+$: m/z = 471.1165, found: m/z = 471.1169.

*1.24 (2,4-Dimethoxy-3-(4-methoxybenzyl)-6-(2-(3-methoxyphenyl)ethyl)phenyl)(4-methoxyphenyl)methanol (**38**)*

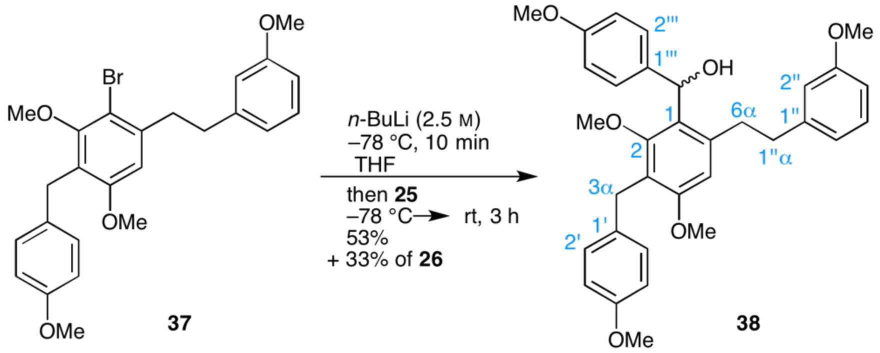

Under an argon atmosphere, 2-bromo-3,5-dimethoxy-4-(4-methoxybenzyl)-1-(2-(3-methoxyphenyl)ethyl)benzene (**37**, 2.41 g, 5.11 mmol, 1.00 eq) was dissolved in anhydrous THF (50 mL) and *n*-BuLi (2.5 M in hexanes, 2.25 mL, 5.62 mmol, 1.10 eq) was slowly added at –78 °C. The reaction mixture was stirred for 10 min at –78 °C, followed by the dropwise addition of 4-methoxybenzaldehyde (**25**, 704 µL, 5.79 mmol, 1.03 eq). The mixture was stirred for 3 h at room temperature, quenched by the addition of a saturated solution of NH_4Cl (30 mL) and extracted with Et_2O (3 x 30 mL). The combined organic layers were dried over Na_2SO_4 and the solvent was removed under reduced pressure. After flash-chromatographic purification on silica (cHex/EtOAc 10:1), the title compound was obtained as a colorless oil (1.62 g, 3.06 mmol, 54%) alongside a fraction containing compound **26** as a colorless oil (718 mg, 1.83 mmol, 33%).

R_f: 0.13 (SiO_2, cHex/EtOAc 10:1).

^{1}H-NMR, COSY (600.1 MHz, $CDCl_3$): δ/ppm = 7.28–7.25 (m, 2H, *H*-2''', *H*-6'''), 7.18 (t, 1H, *J* = 7.8 Hz, *H*-5''), 7.10–7.06 (m, 2H, *H*-2', H-6'), 6.86–6.82 (m, 2H, *H*-3''', *H*-5'''), 6.79–6.76 (m, 2H, *H*-3', *H*-5'), 6.75–6.71 (m, 2H, *H*-4'', *H*-6''), 6.66–6.64 (m, 1H, *H*-2''), 6.52 (s, 1H, *H*-5), 6.09 (d, *J* = 9.4 Hz, C*H*OH), 3.92 (s, 2H, 3*α*-C*H*$_2$), 3.89 (d, 1H, *J* = 9.4 Hz, CHO*H*), 3.78 (s, 3H, 4'''-OC*H*$_3$), 3.76 (s, 3H, 4'-OC*H*$_3$), 3.75 (s, 3H, 3''-OC*H*$_3$), 3.74 (s, 3H, 4-OC*H*$_3$), 3.13 (s, 3H, 2-OC*H*$_3$), 3.02–2.80 (m, 4H, 6*α*-C*H*$_2$, 1''*α*-C*H*$_2$).

^{13}C-NMR, HSQC, HMBC (150.9 MHz, $CDCl_3$): δ/ppm = 159.7 (C_q-3''), 158.5 (C_q-4'''), 158.2 (C_q-2), 158.1 (C_q-4), 157.6 (C_q-4'), 143.3 (C_q-1''), 139.7 (C_q-6), 137.8 (C_q-1'''), 133.2 (C_q-1'), 129.5 (*C*-5''), 129.2 (2C, *C*-2', *C*-6'), 127.7 (C_q-1), 127.0 (2C, *C*-2''', *C*-6'''), 121.4 (C_q-3), 121.0 (*C*-6''), 114.2 (*C*-2''), 113.6 (2C, *C*-3', *C*-5'), 113.6 (2C, *C*-3''', *C*-5'''), 111.6 (*C*-4''), 108.6 (*C*-5), 70.0 (CHOH), 62.3 (2-OCH$_3$), 55.8 (4-OCH$_3$), 55.3 (3C, 4'-OCH$_3$, 3''-OCH$_3$, 4'''-OCH$_3$), 38.0 (1''*α*-CH$_2$), 35.8 (6*α*-CH$_2$), 28.7 (3*α*-CH$_2$).

IR: $\bar{\nu}$ [cm^{-1}] = 3492, 2997, 2938, 2834, 1600, 1583, 1509, 1463, 1246, 1172, 1111, 1037.

ESI-MS: m/z = 511.3 (100%, [M – OH]$^+$, calc. 511.2).

ESI-HRMS: calc. for $[C_{33}H_{36}O_6 + Na]^+$: m/z = 551.2404, found: m/z = 551.2409.

*1.25 1,3-Dimethoxy-2,4-bis(4-methoxybenzyl)-5-(2-(3-methoxyphenyl)ethyl)benzene (**41**)*

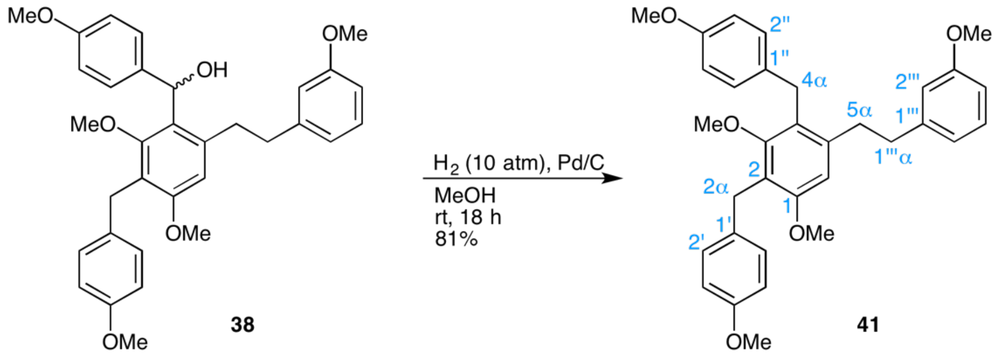

A solution of (2,4-dimethoxy-3-(4-methoxybenzyl)-6-(2-(3-methoxyphenyl)ethyl)phenyl)(4-methoxyphenyl)-methanol (**38**, 50 mg, 95.6 µmol, 1.00 eq) and Pd/C (10 wt-% Pd, 10 mg) in MeOH (1 mL) was stirred at room temperature for 18 h under a hydrogen atmosphere (10 atm, autoclave). After filtration over a short plug of silica and concentration under reduced pressure, the title compound was obtained as a colorless oil (39.8 mg, 77.6 µmol, 81%).

R_f: 0.39 (SiO_2, cHex/EtOAc 10:1).

^{1}H-NMR, COSY (300.1 MHz, $CDCl_3$): δ/ppm = 7.17 (t, 1H, *J* = 7.9 Hz, *H*-5'''), 7.16–7.14 (m, 2H, *H*-2', *H*-6'), 7.02–6.98 (m, 2H, *H*-2'', *H*-6''), 6.81–6.76 (m, 4H, *H*-3', *H*-5', *H*-3'', *H*-5''), 6.72 (ddd, 1H, *J* = 7.9 Hz, *J* = 2.6 Hz, *J* = 1.1 Hz, *H*-4'''), 6.67

(dt, 1H, *J* = 7.9 Hz, *J* = 1.1 Hz, *H*-6'''), 6.55 (dd, 1H, *J* = 2.6 Hz, *J* = 1.1 Hz, *H*-2'''), 6.54 (s, 1H, *H*-6), 3.99 (s, 2H, 2α-C*H*$_2$), 3.97 (s, 2H, 4α-C*H*$_2$), 3.77 (s, 3H, 4''-OC*H*$_3$), 3.76 (s, 3H, 4'-OC*H*$_3$), 3.75 (s, 3H, 1-OC*H*$_3$), 3.74 (s, 3H, 3'''-OC*H*$_3$), 3.49 (s, 3H, 3-OC*H*$_3$), 2.81–2.77 (m, 2H, 5α-C*H*$_2$), 2.70–2.65 (m, 2H, 1'''α-C*H*$_2$).

^{13}C-NMR, HSQC, HMBC (75.5 MHz, CDCl$_3$): δ/ppm = 159.7 (C$_q$-3'''), 158.1 (C$_q$-3), 157.7 (C$_q$-4''), 157.6 (C$_q$-4'), 157.1 (C$_q$-1), 143.6 (C$_q$-1'''), 140.6 (C$_q$-5), 134.0 (C$_q$-1'), 133.8 (C$_q$-1''), 129.4 (*C*-5''), 129.3 (2C, *C*-2', *C*-6'), 129.0 (2C, *C*-2'', *C*-6''), 124.3 (C$_q$-4), 120.9 (C$_q$-2), 120.8 (*C*-6''), 114.2 (*C*-2'''), 113.8 (2C, *C*-3'', *C*-5''), 113.6 (2C, *C*-3', *C*-5'), 111.5 (*C*-4'''), 108.3 (*C*-6), 62.0 (3-OCH$_3$), 55.7 (1-OCH$_3$), 55.4 (4'-OCH$_3$), 55.3 (2C, 4''-OCH$_3$, 3'''-OCH$_3$), 37.7 (1'''α-CH$_2$), 35.5 (5α-CH$_2$), 30.8 (4α-CH$_2$), 28.9 (2α-CH$_2$).

IR: $\tilde{\nu}$ [cm^{-1}] = 2997, 2936, 2834, 1601, 1583, 1509, 1463, 1242, 1112, 1037, 808, 697.

ESI-MS: m/z = 504.2 (100%, [M–MeOPh]$^+$, calc. 504.2).

ESI-HRMS: calc. for [C$_{33}$H$_{36}$O$_5$ + K]$^+$: m/z = 551.2194, found: m/z = 551.2197.

Gram quantities of **41** (1.05 g, 2.05 mmol) could be obtained in 77% yield starting from **38** (1.40 g, 2.65 mmol) by using 140 mg of Pd/C (10 wt-% Pd) under otherwise identical conditions.

*1.26 2,4,8-Trimethoxy-3-(4-methoxybenzyl)-5-(4-methoxyphenyl)-10,11-dihydro-5H-dibenzo-[a,d][7]-annulene (**40**)*

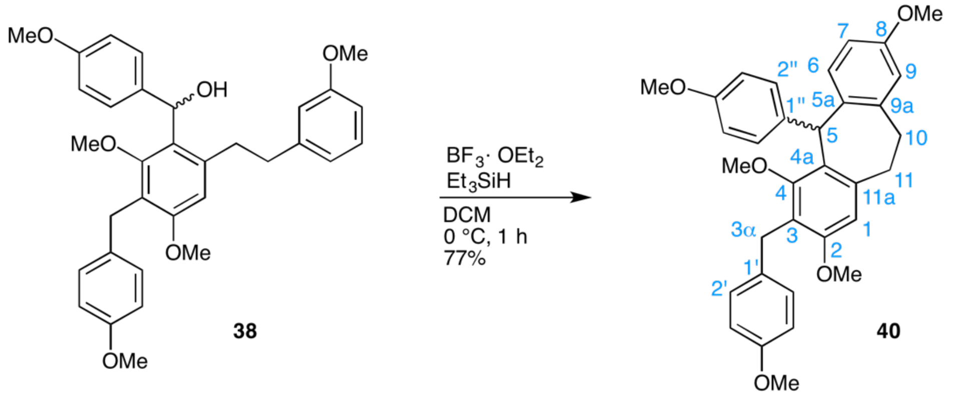

Under an argon atmosphere, a solution of (2,4-dimethoxy-3-(4-methoxybenzyl)-6-(2-(3-methoxy-phenyl)ethyl)phenyl)(4-methoxyphenyl)methanol (**38**, 600 mg, 1.14 mmol, 1.00 eq) in anhydrous DCM (10 mL) was cooled to 0 °C and Et$_3$SiH (363 µL, 2.27 mmol, 2.00 eq) was added, followed by the dropwise addition of BF$_3$·OEt$_2$ (154 µL, 1.25 mmol, 1.10 eq). The solution was stirred for 1 h at 0 °C, quenched by the addition of saturated solution of NaHCO$_3$ (10 mL) and extracted with DCM (3 x 15 mL). The combined organic layers were dried over Na$_2$SO$_4$ and the solvent was removed under reduced pressure. After flash-chromatographic purification on silica (^{c}Hex/EtOAc 15:1), the title compound was obtained as a colorless oil (448 mg, 0.88 mmol, 77%) alongside a fraction containing compound **39** (83 mg, 0.16 mmol, 14%).

R$_f$: 0.39 (SiO$_2$, ^{c}Hex/EtOAc 10:1).

^{1}H-NMR, COSY (600.1 MHz, CDCl$_3$): δ/ppm = 7.24 (d, 1H, *J* = 8.3 Hz, *H*-6), 7.18–7.14 (m, 2H, *H*-2', *H*-6'), 6.81–6.76 (m, 4H, *H*-3', *H*-5', *H*-2'', *H*-6''), 6.74–6.70 (m, 3H, *H*-7, *H*-3'', *H*-5''), 6.69 (d, 1H, *J* = 2.7 Hz, *H*-9), 6.50 (s, 1H, *H*-1), 5.71 (s, 1H, *H*-5), 4.00 (s, 2H, 3α-C*H*$_2$), 3.79 (s, 3H, 8-OC*H*$_3$), 3.77 (s, 3H, 2-OC*H*$_3$), 3.76 (s, 3H, 4'-OC*H*$_3$), 3.74 (s, 3H, 4''-OC*H*$_3$), 3.58 (s, 3H, 4-OC*H*$_3$) 3.11–3.02 (m, 2H, 10-C*H*$_a$, 11-C*H*$_a$), 2.82–2.74 (m, 1H, 10-C*H*$_b$), 2.61–2.55 (m, 1 H, 11-C*H*$_b$).

^{13}C-NMR, HSQC, HMBC (150.9 MHz, CDCl$_3$): δ/ppm = 158.5 (C$_q$-8), 157.6 (C$_q$-4'), 157.4 (C$_q$-4''), 157.2 (C$_q$-4), 157.1 (C$_q$-2), 141.2 (C$_q$-9a), 140.4 (C$_q$-11a), 138.7 (C$_q$-1''), 134.0 (C$_q$-1'), 133.3 (*C*-6), 132.7 (C$_q$-5a), 129.4 (2C, *C*-2', *C*-6'), 128.2 (2C, *C*-2'', *C*-6''), 128.0 (C$_q$-4a), 120.3 (C$_q$-3), 116.4 (*C*-9), 113.6 (2C, *C*-3', *C*-5'), 113.4 (2C, *C*-3'', *C*-5''), 111.3 (*C*-7), 108.9 (*C*-1), 62.5 (4-OCH$_3$), 55.7 (2-OCH$_3$), 55.3 (3C, 8-OCH$_3$, 4'-OCH$_3$, 4''-OCH$_3$), 46.1 (*C*-5), 32.9 (*C*-10), 32.4 (*C*-11), 29.0 (3α-CH$_2$).

IR: $\tilde{\nu}$ [cm^{-1}] = 2997, 2935, 2906, 2834, 1604, 1582, 1508, 1462, 1244, 1176, 1113, 1036.

ESI-MS: m/z = 511.3 (100%, [M+H]$^+$, calc. 511.2).

ESI-HRMS: calc. for [C$_{33}$H$_{34}$O$_5$ + K]$^+$: m/z = 549.2038, found: m/z = 549.2039.

*Isolated Side Product: 1,3-Dimethoxy-5-(2-(5-methoxy-2-(4-methoxybenzyl)phenyl)ethyl)-2-(4-methoxybenzyl)benzene (**39**)*

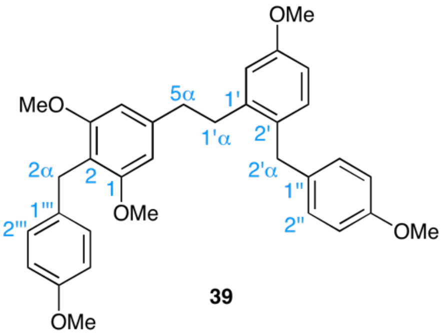

39

R$_f$: 0.33 (SiO_2, ^{c}Hex/EtOAc 10:1).

^{1}H-NMR, COSY (600.1 MHz, $CDCl_3$): δ/ppm = 7.19–7.15 (m, 2H, *H*-2''', *H*-6'''), 7.03 (d, 1H, *J* = 8.2 Hz, *H*-3'), 7.02–6.99 (m, 2H, *H*-2'', *H*-6''), 6.82–6.78 (m, 2H, *H*-3'', *H*-5''), 6.77–6.74 (m, 3H, *H*-6', *H*-3''', *H*-5'''), 6.72 (dd, 1H, *J* = 8.2 Hz, *J* = 2.7 Hz, *H*-4'), 6.24 (s, 2H, *H*-4, *H*-6), 3.89 (s, 2H, 2*α*-C*H*$_2$), 3.86 (s, 2H, 2'*α*-C*H*$_2$), 3.77–3.76 (m, 6H, 4''-OC*H*$_3$, 4'''-OC*H*$_3$,), 3.75 (m, 6H, 1-OC*H*$_3$, 3-OC*H*$_3$), 3.74 (s, 3H, 5'-OC*H*$_3$), 2.83–2.79 (m, 2H, 1'*α*-C*H*$_2$), 2.70–2.59 (m, 2H, 5*α*-C*H*$_2$).

^{13}C-NMR, HSQC, HMBC (150.9 MHz, $CDCl_3$): δ/ppm = 158.3 (*C*$_q$-5), 158.1 (2C, *C*$_q$-1, *C*$_q$-2), 157.9 (*C*$_q$-4''), 157.5 (*C*$_q$-4'''), 141.6 (*C*$_q$-1'), 141.3 (*C*$_q$-5), 134.3 (*C*$_q$-1'''), 133.5 (*C*$_q$-1''), 131.5 (*C*-3'), 131.3 (*C*$_q$-2'), 129.6 (2C, *C*-2'', *C*-6''), 129.5 (2C, *C*-2''', *C*-6'''), 115.8 (*C*$_q$-2), 115.3 (*C*-6'), 113.9 (2C, *C*-3'', *C*-5''), 113.5 (2C, *C*-3''', *C*-5'''), 111.3 (*C*-4'), 104.2 (2C, *C*-4, *C*-6), 55.9 (2C, 1-OCH$_3$, 3-OCH$_3$), 55.3 (3C, 5'-OCH$_3$, 4''-OCH$_3$, 4'''-OCH$_3$), 38.1 (5*α*-CH$_2$), 37.5 (2'*α*-CH$_2$), 35.4 (1'*α*-CH$_2$), 27.7 (2*α*-CH$_2$).

IR: $\bar{\nu}$ [cm^{-1}] = 2998, 2935, 2834, 1607, 1587, 1509, 1458, 1420, 1245, 1175, 1118, 1038, 821.

ESI-MS: m/z = 535.3 (100%, [M+Na]$^+$, calc. 535.2).

ESI-HRMS: calc. for [$C_{33}H_{36}O_5$ + K]$^+$: m/z = 551.2194, found: m/z = 551.2190.

*1.27 2,4-Bis(4-hydroxybenzyl)-5-(2-(3-hydroxyphenyl)ethyl)benzene-1,3-diol (**2**)*

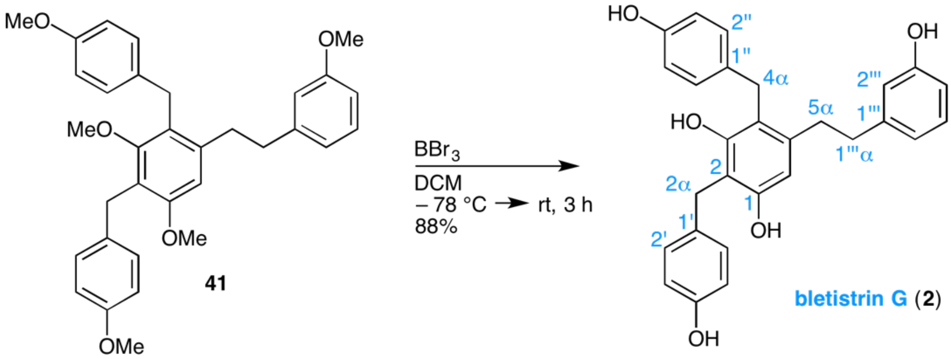

41 **bletistrin G (2)**

A solution of 1,3-dimethoxy-2,4-bis(4-methoxybenzyl)-5-(2-(3-methoxyphenyl)ethyl)benzene (**41**, 920 mg, 1.79 mmol, 1.00 eq) in anhydrous DCM (35 mL) was cooled to –78 °C, followed by the dropwise addition of BBr_3 (1 M in DCM, 11.6 mL, 11.6 mmol, 6.50 eq). The mixture was allowed to reach room temperature, stirred for 3 h, quenched by the addition of ice water (30 mL) and the reaction was extracted with EtOAc (3 x 50 mL). The combined organic layers were dried over Na_2SO_4 and the solvent was removed under reduced pressure. After recrystallization from EtOAc/PE (3:1), the title compound was obtained as a colorless solid (694 mg, 1.57 mmol, 88%) alongside compound **42** which was isolated from the mother liquor (38 mg, 0.09 mmol, 5%).

Mp.: 138.3–140.1 °C (EtOAc/PE 3:1).

R$_f$: 0.35 (SiO_2, ^{c}Hex/EtOAc 1:1 + 1% AcOH).

^{1}H-NMR, COSY (300.1 MHz, $CDCl_3$): δ/ppm = 7.09–7.05 (m, 2H, *H*-2', *H*-6'), 7.03 (t, 1H, J = 7.9 Hz, *H*-5'''), 6.93–6.89 (m, 2H, *H*-2'', *H*-6''), 6.66–6.61 (m, 4H, *H*-3', *H*-5', *H*-3'', *H*-5''), 6.57 (d, 1H, J = 7.9 Hz, *H*-4'''), 6.55–6.52 (m, 2H, *H*-2''', *H*-6'''), 6.31 (s, 1H, *H*-6), 3.92 (s, 2H, 2*α*-C*H*$_2$), 3.90 (s, 2H, 4*α*-C*H*$_2$), 2.71–2.64 (m, 2H, 5*α*-C*H*$_2$), 2.59–2.51 (m, 2H, 1'''*α*-C*H*$_2$).

^{13}C-NMR, HSQC, HMBC (75.5 MHz, $CDCl_3$): δ/ppm = 158.3 (C$_q$-3'''), 156.1 (C$_q$-4''), 156.0 (C$_q$-4'), 155.3 (C$_q$-1), 155.0 (C$_q$-3), 145.1 (C$_q$-1'''), 140.9 (C$_q$-5), 133.9 (2C, C$_q$-1', C$_q$-1''), 130.4 (2C, *C*-2', *C*-6'), 130.2 (*C*-5'''), 130.1 (2C, *C*-2'', *C*-6''), 120.7 (*C*-6''), 119.0 (C$_q$-4), 116.2 (*C*-2'''), 115.9 (2C, *C*-3'', *C*-5''), 115.7 (2C, C-3', C-5'), 114.9 (C$_q$-2), 113.7 (*C*-4'''), 109.5 (*C*-6), 38.8 (1'''*α*-CH$_2$), 36.7 (5*α*-CH$_2$), 31.3 (4*α*-CH$_2$), 29.1 (2*α*-CH$_2$).

IR: $\bar{\nu}$ [cm^{-1}] = 3329, 2937, 2864, 1613, 1595, 1511, 1433, 1359, 1229, 1174, 839, 819.

ESI-MS: m/z = 443.2 (100%, $[M+H]^+$, calc. 443.2).

ESI-HRMS: calc. for $[C_{28}H_{26}O_5 + H]^+$: m/z = 443.1853, found: m/z = 443.1851.

*Isolated Side Product: 3-(4-Hydroxybenzyl)-5-(4-hydroxyphenyl)-10,11-dihydro-5H-dibenzo-[a,d][7]annulene-2,4,8-triol (**42**)*

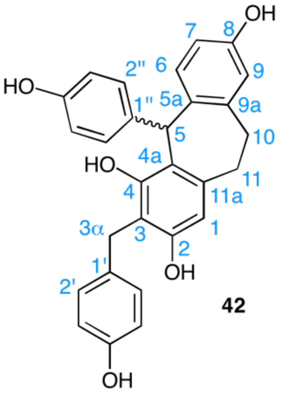

42

Mp.: Decomposition starting from 126 °C (EtOAc/PE 3:1).

R_f: 0.43 (SiO_2, ^{c}Hex/EtOAc 1:1 + 1% AcOH).

^{1}H-NMR, COSY (600.1 MHz, MeOD): δ/ppm = 7.12–7.04 (m, 3H, *H*-6, *H*-2', *H*-6'), 6.66–6.61 (m, 4H, *H*-3', *H*-5', *H*-2'', *H*-6''), 6.59–6.53 (m, 4H, *H*-7, *H*-9, *H*-3'', *H*-5''), 6.23 (s, 1H, *H*-1), 5.73 (s, 1H, *H*-5), 3.96 (d, 1H, J = 15.1 Hz, 3α-CH_a), 3.93 (d, 1H, J = 15.1 Hz, 3α-CH_b), 3.00–2.91 (m, 2H, 10-CH_a, 11- CH_a), 2.63–2.54 (m, 1H, 10-CH_b), 2.48–2.41 (m, 1 H, 11-CH_b).

^{13}C-NMR, HSQC, HMBC (150.9 MHz, MeOD): δ/ppm = 156.9 (C_q-8), 156.0 (C_q-4'), 155.7 (C_q-4''), 155.3 (C_q-2), 154.2 (C_q-4), 142.9 (C_q-9a), 140.9 (C_q-11a), 139.3 (C_q-5a), 134.3 (*C*-6), 133.7 (C_q-1'), 133.5 (C_q-1''), 130.3 (2C, *C*-2', *C*-6'), 129.1 (2C, *C*-2'', *C*-6''), 123.3 (C_q-4a), 117.9 (*C*-9), 115.7 (2C, *C*-3', *C*-5'), 115.4 (2C, *C*-3'', *C*-5''), 114.7 (C_q-3), 113.4 (*C*-7), 110.2 (*C*-1), 46.8 (*C*-5), 33.9 (*C*-10), 33.5 (*C*-11), 29.2 (3α-CH_2).

IR: $\bar{\nu}$ [cm^{-1}] = 3377, 3340, 2934, 1604, 1509, 1434, 1359, 1228, 1173, 1103, 1040, 819.

ESI-MS: m/z = 441.2 (100%, $[M+H]^+$, calc. 441.2).

ESI-HRMS: calc. for $[C_{28}H_{24}O_5 + H]^+$: m/z = 441.1697, found: m/z = 441.1696.

2. Crystallographic data and structure refinement for shancigusin C (1)

Crystal structure analysis was performed on an STOE IPDS 2T instrument. Data analysis took place via software programs SIR-2004 and SHELXL-2018, whereas visualization of molecule structures was realized with the software Mercury.

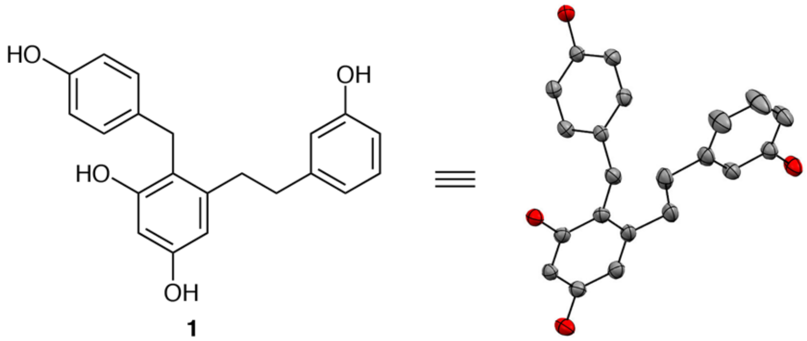

1

molecular formula	$C_{21}H_{20}O_4$
molecular mass	336.38 gmol^{-1}
space group	P 2_1/n (monoclinic)
absorption	μ = 0.091 mm^{-1}
crystal size	0.04 x 0.11 x 0.53 mm³, colorless plate
lattice constant	a = 11.6199(6) Å
(calculated from	b = 5.3749(2) Å $ß$ = 99.898(4)°
4209 reflexes with	c = 27.5470(14) Å
2.62 ° < θ < 28.28 °)	V = 1694.86(14) Å^3 z = 4 F(000) = 712
temperature	–80 °C
density	d = 1.318 gcm^{-3}

Data Collection

diffractometer	STOE IPDS 2T
radiation	Mo-K$_\alpha$ graphite monochromator
scan-type	ω scans
scan-width	1°
range	2° ≤ θ ≤ 28°
	–15 ≤ h ≤ 11 –6 ≤ k ≤ 7 –36 ≤ l ≤ 36
number of reflexes:	
measured	9654
free	4197 (R_{int} = 0.0313)
observed	2520 (∣F∣/σ(F) > 4.0)

Correction of Data, Structural Solution and Refinement

corrections	Lorentz- and polarisation correction
solution	programm: SIR-2004 (direct methodes)
refinement	programm: SHELXL-2018 (full-matrix procedure). 303 refined parameters, weighted refinement:

	w = 1/[$\sigma^2(F_o^2)$ + $(0.055*P)^2$ + 0.59*P] where P = (Max(F_o^2,0) + 2*F_c^2)/3. Hydrogen atoms localized and refined isotropically, non-hydrogen atoms refined anisotropically.
discrepancy factor	wR2 = 0.1353 (R1 = 0.051 for observed reflexes, wR2 = 0.1001 for all reflexes)
goodness of fit	S = 1.018
maximum alteration of parameters	0.001 * e.s.d
maximum peak height in diff. Fourier synthesis	0.29, –0.27 eÅ^{-3}

The atom coordinates and further details are deposited with the Cabridge Crystallographic Data Center and can be retrieved at https://www.ccdc.cam.ac.uk/ using the deposition number CCDC 2081480.

3. NMR-Spectra of the compounds

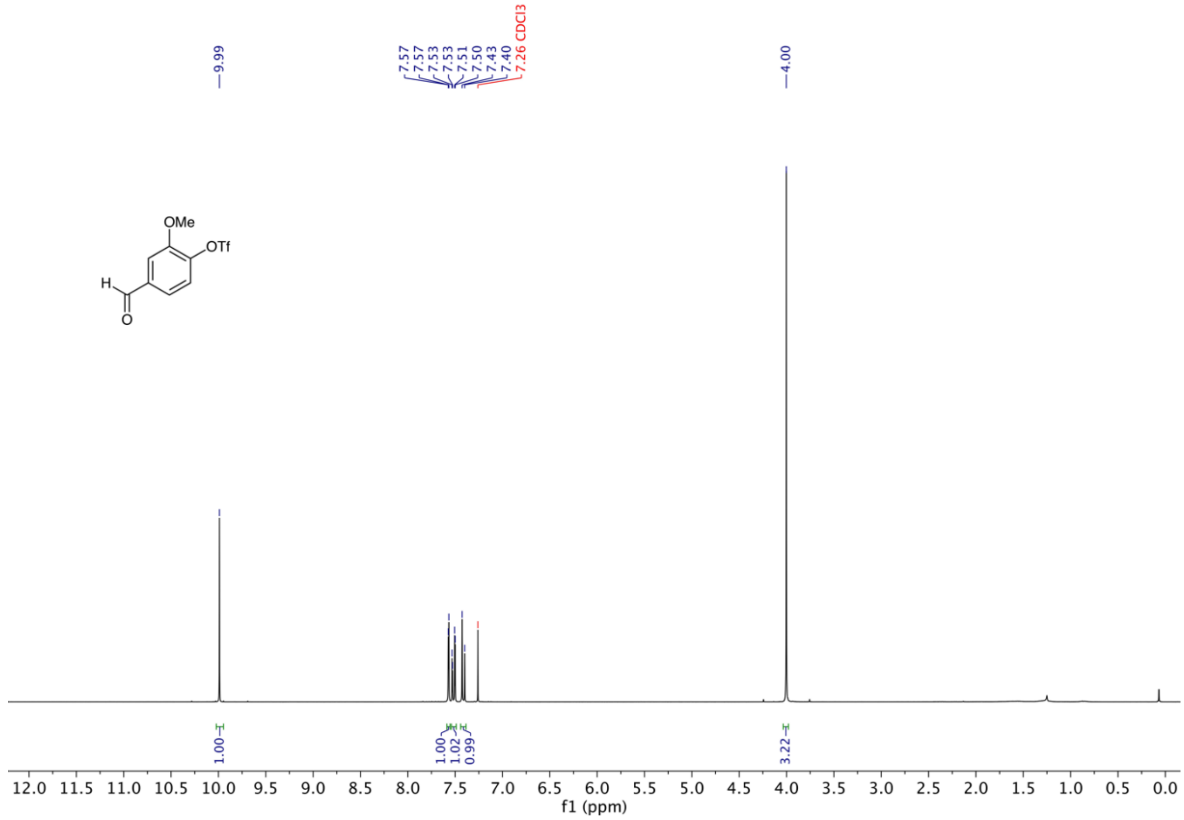

^{1}H-NMR (300.1 MHz, $CDCl_3$): 4-Formyl-2-methoxyphenyl trifluoromethanesulfonate (**13**).

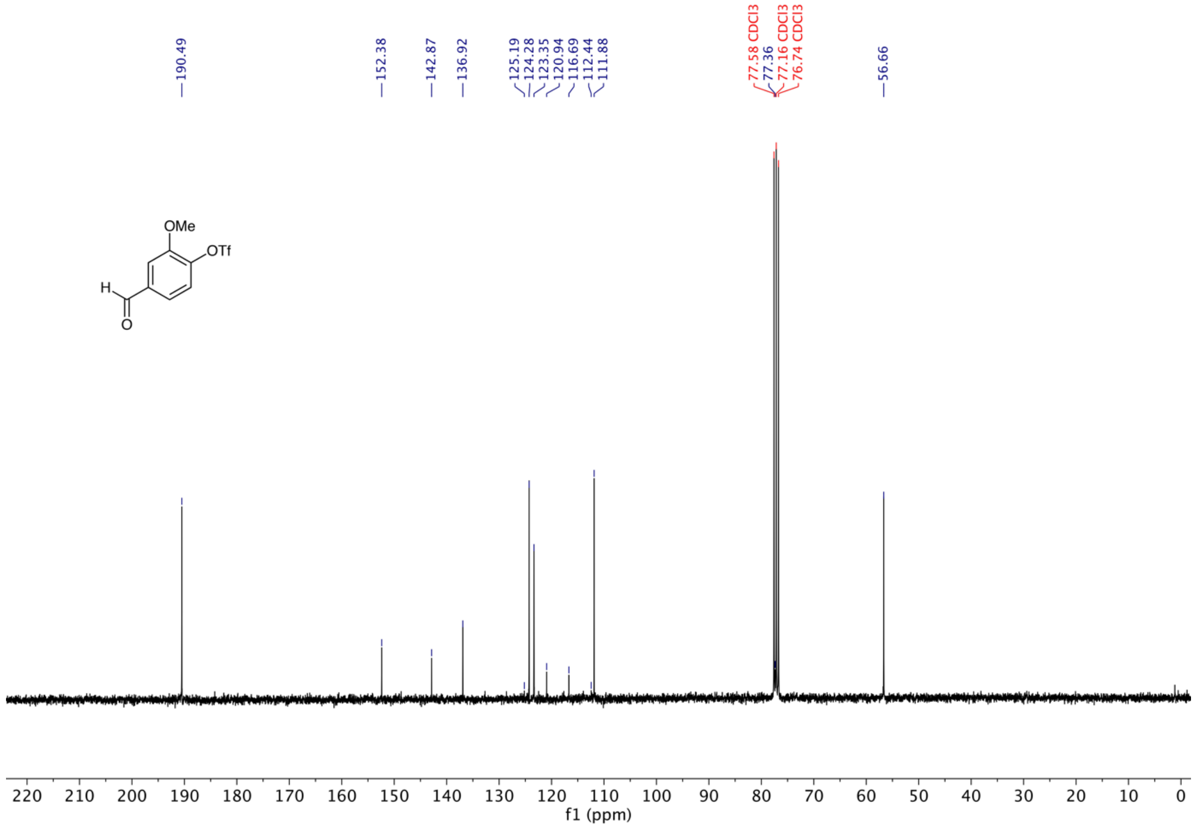

^{13}C-NMR (75.5 MHz, $CDCl_3$): 4-Formyl-2-methoxyphenyl trifluoromethanesulfonate (**13**).

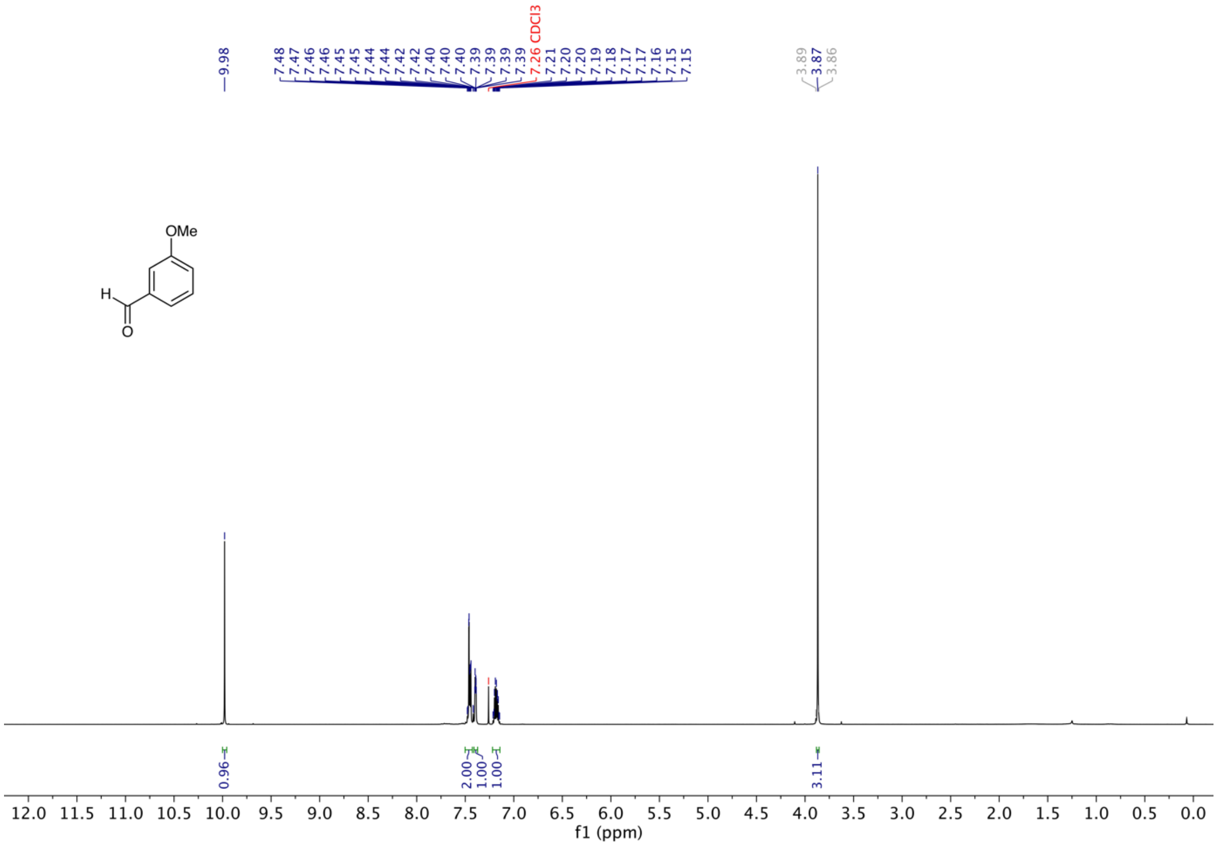

^{1}H-NMR (300.1 MHz, $CDCl_3$): 3-Methoxybenzaldehyde (**8**).

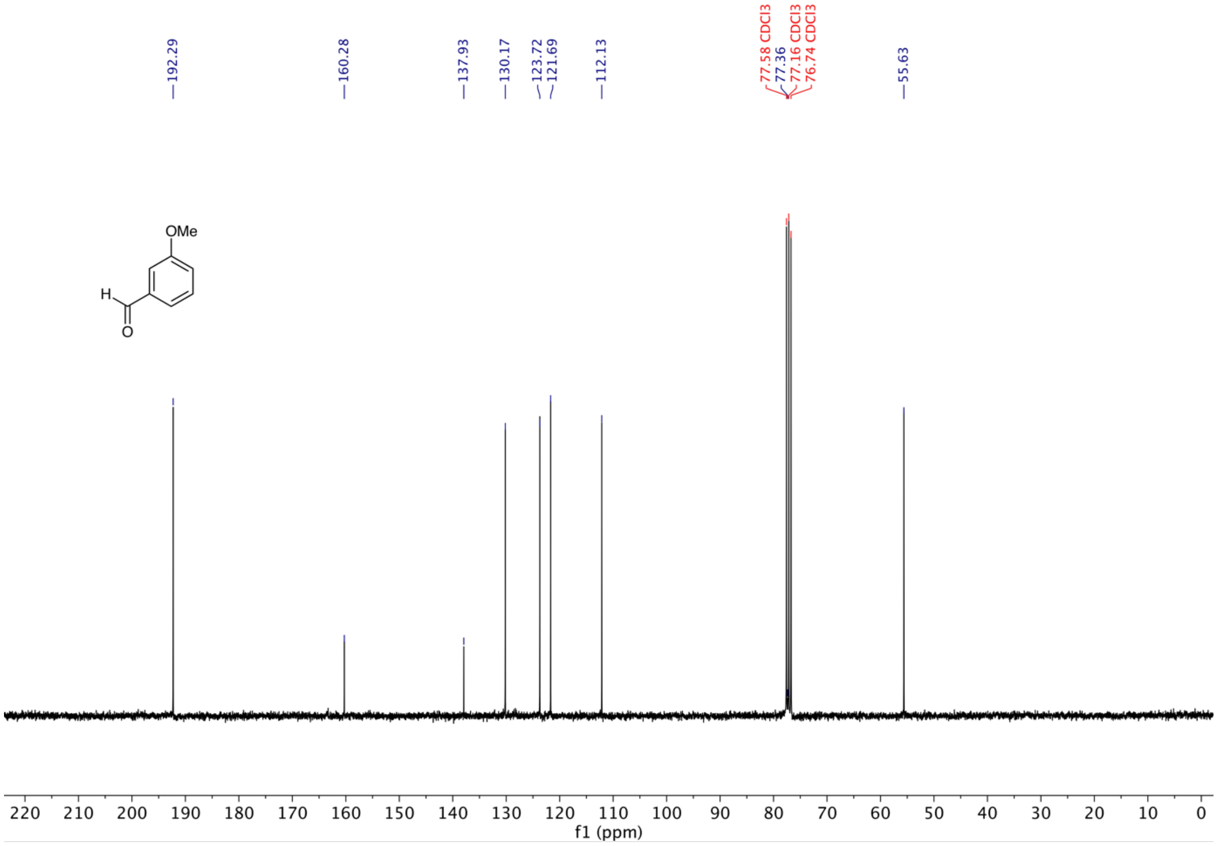

^{13}C-NMR (75.5 MHz, $CDCl_3$): 3-Methoxybenzaldehyde (**8**).

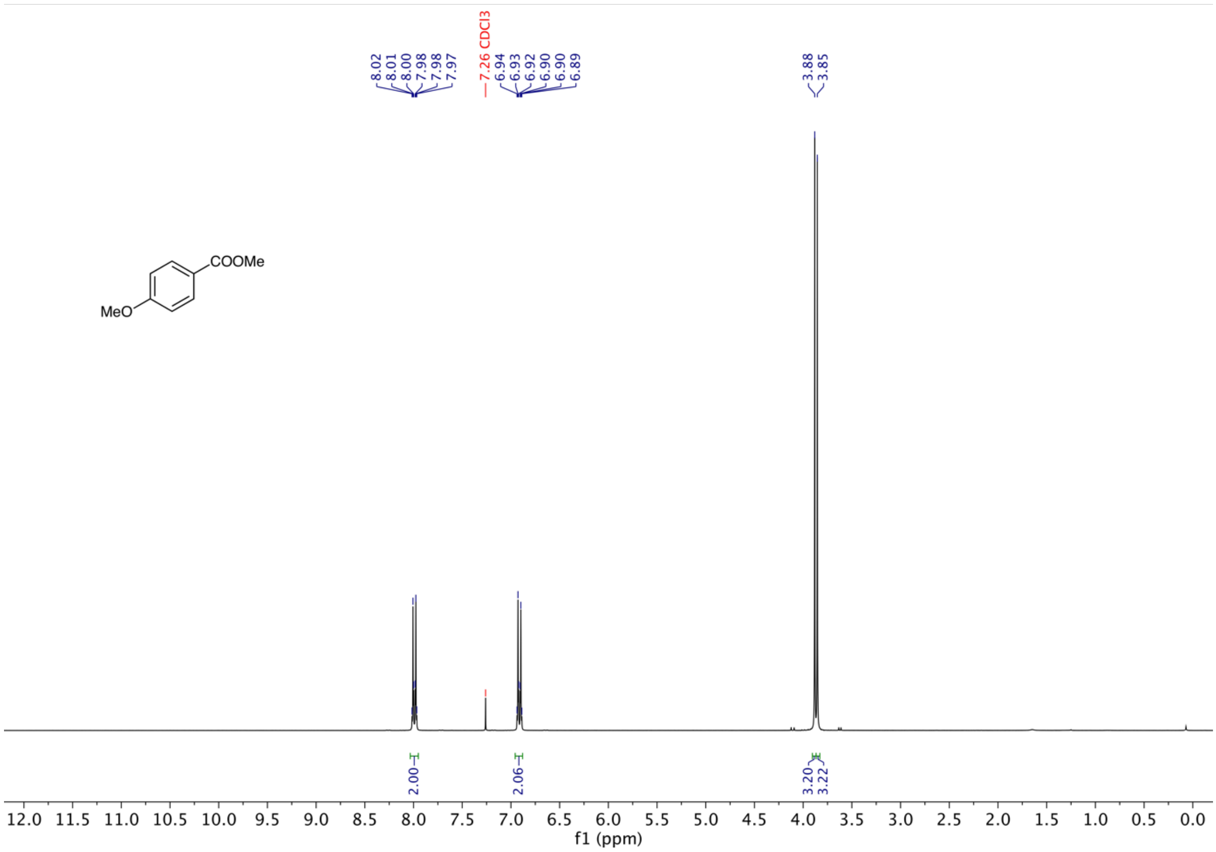

^{1}H-NMR (300.1 MHz, $CDCl_3$): Methyl 4-methoxybenzoate (**14**).

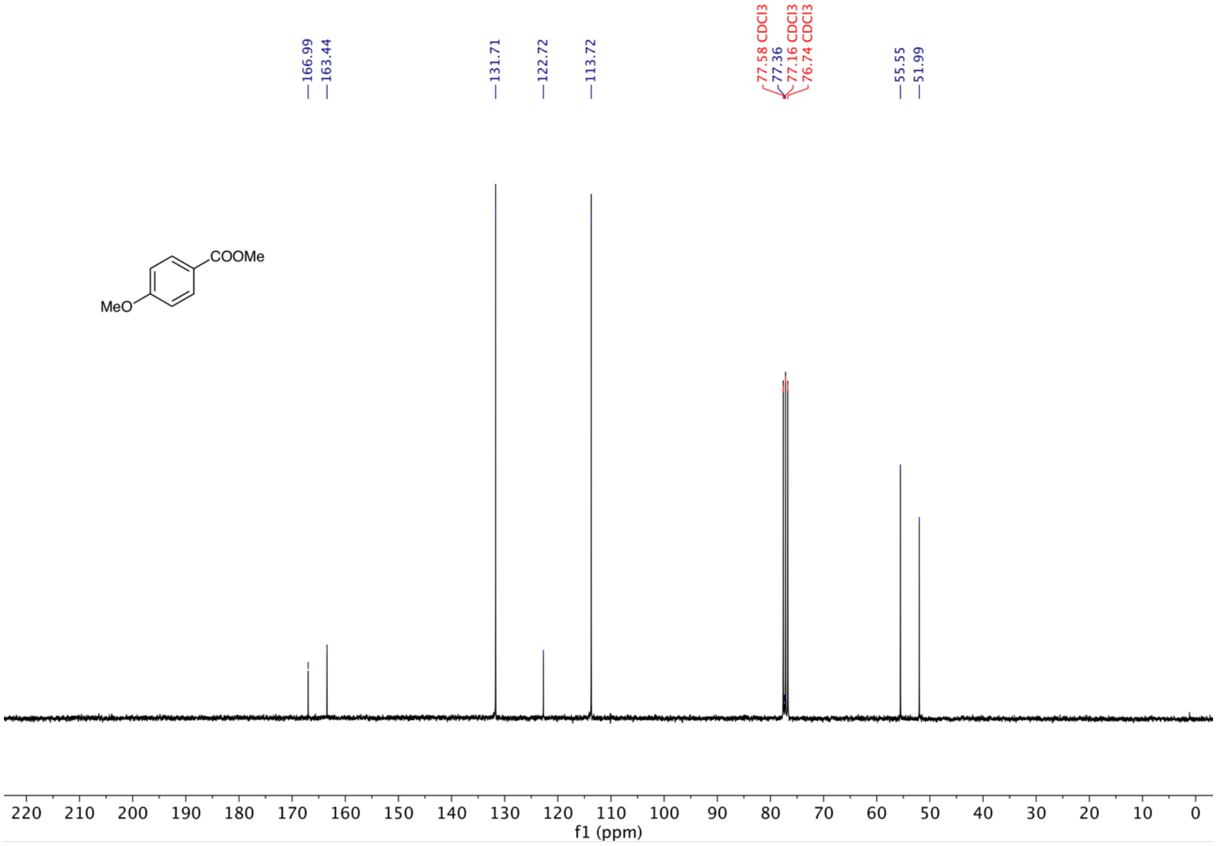

^{13}C-NMR (75.5 MHz, $CDCl_3$): Methyl 4-methoxybenzoate (**14**).

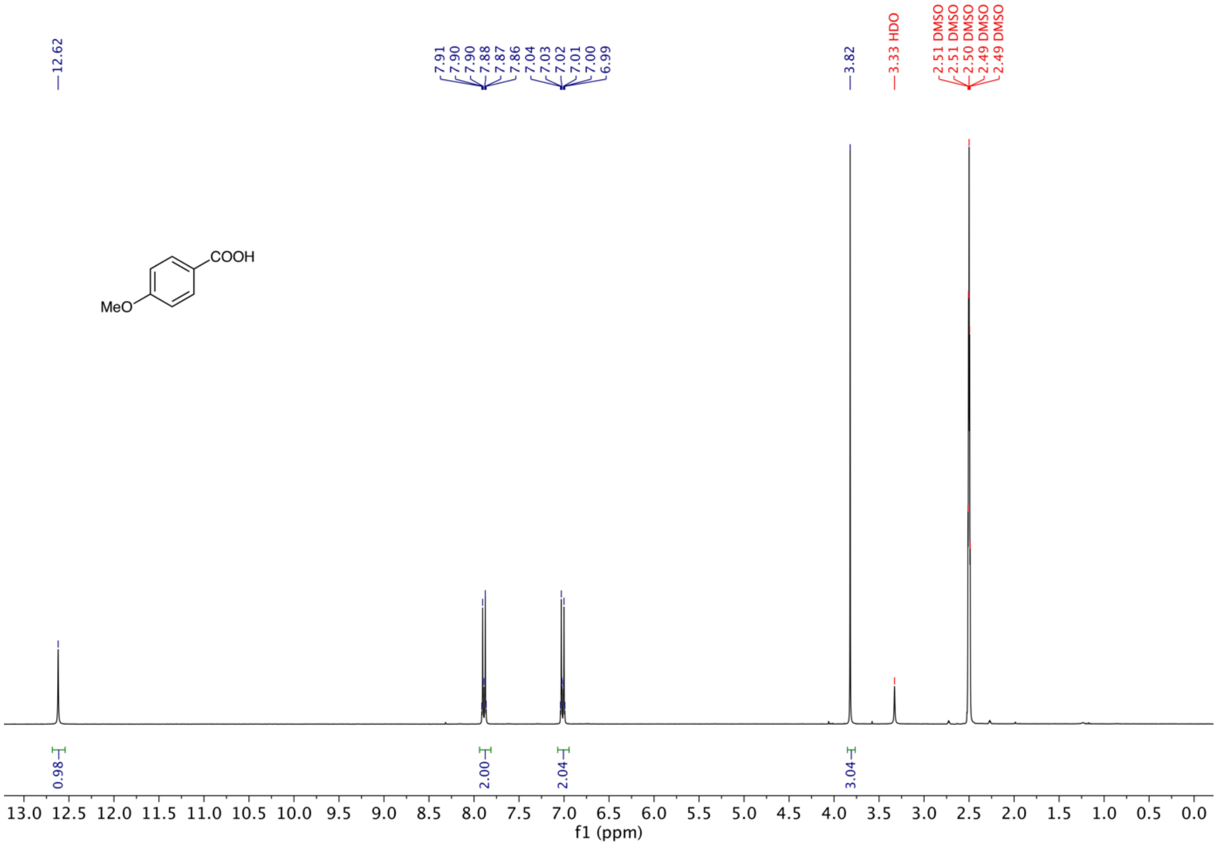

^{1}H-NMR (300.1 MHz, DMSO-d$_6$): 4-Methoxybenzoic acid (**15**).

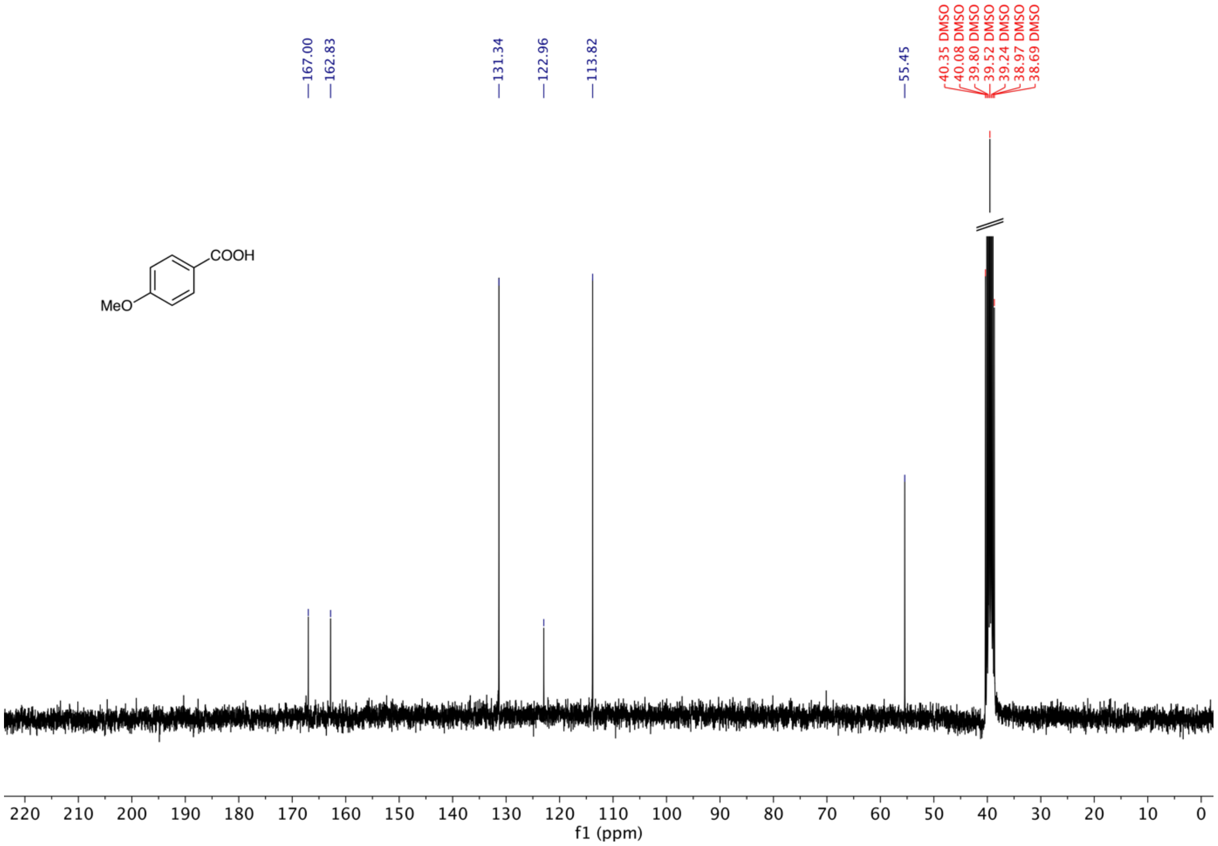

^{13}C-NMR (75.5 MHz, DMSO-d$_6$): 4-Methoxybenzoic acid (**15**).

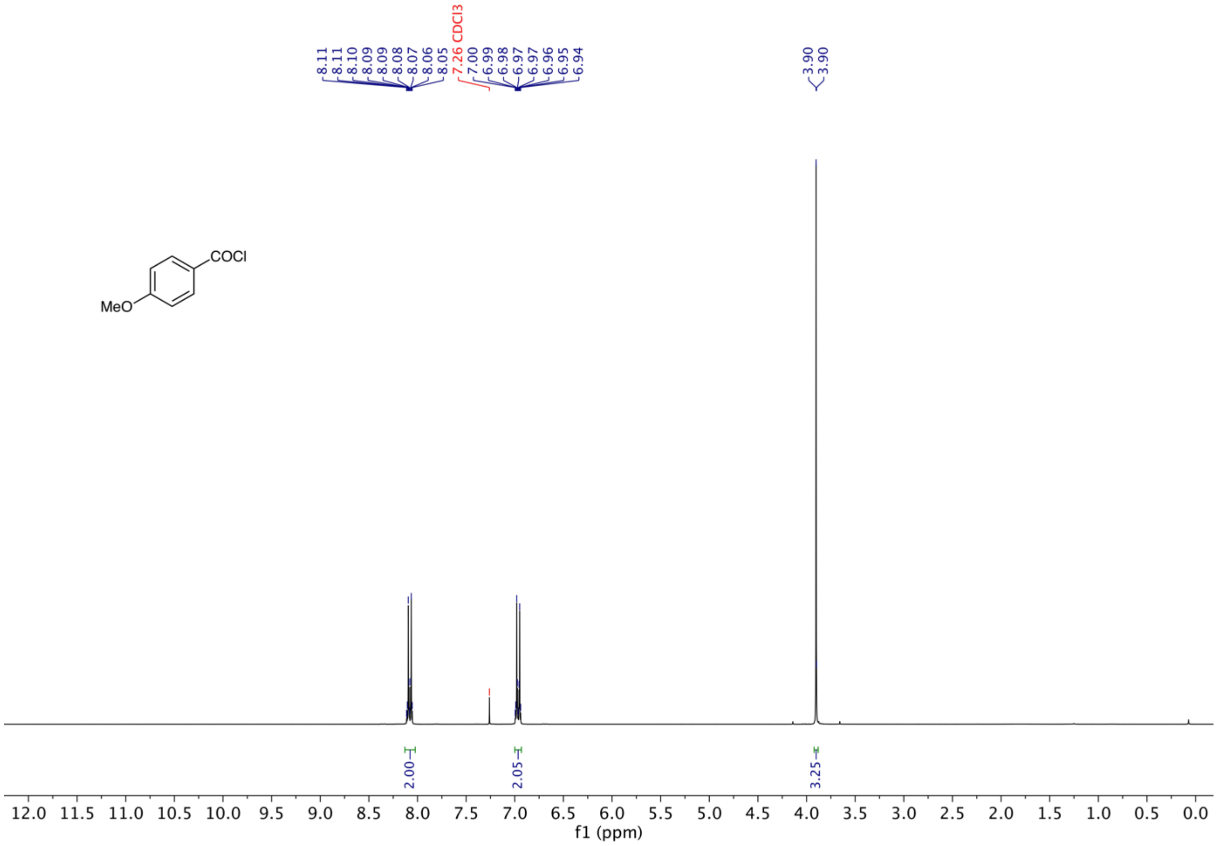

^{1}H-NMR (300.1 MHz, $CDCl_3$): 4-Methoxybenzoyl chloride (**16**).

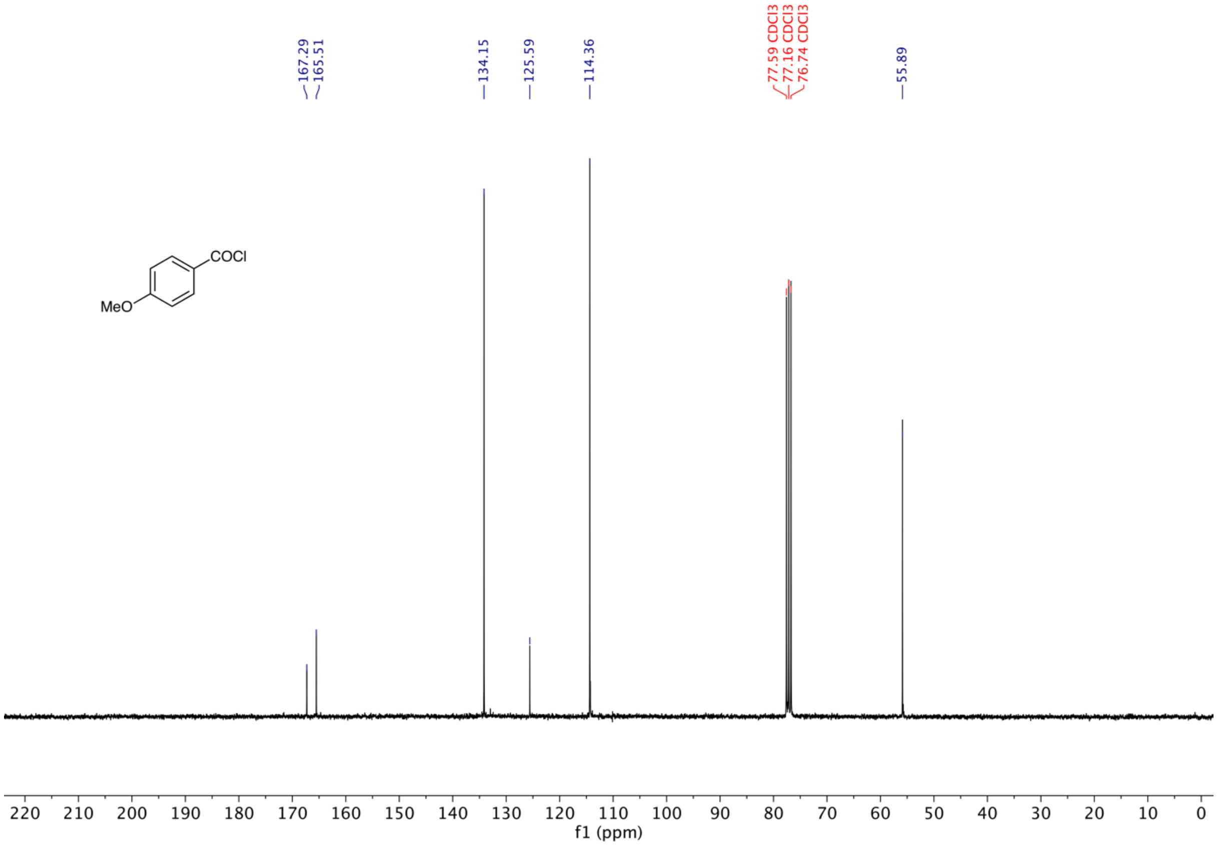

^{13}C-NMR (75.5 MHz, $CDCl_3$): 4-Methoxybenzoyl chloride (**16**).

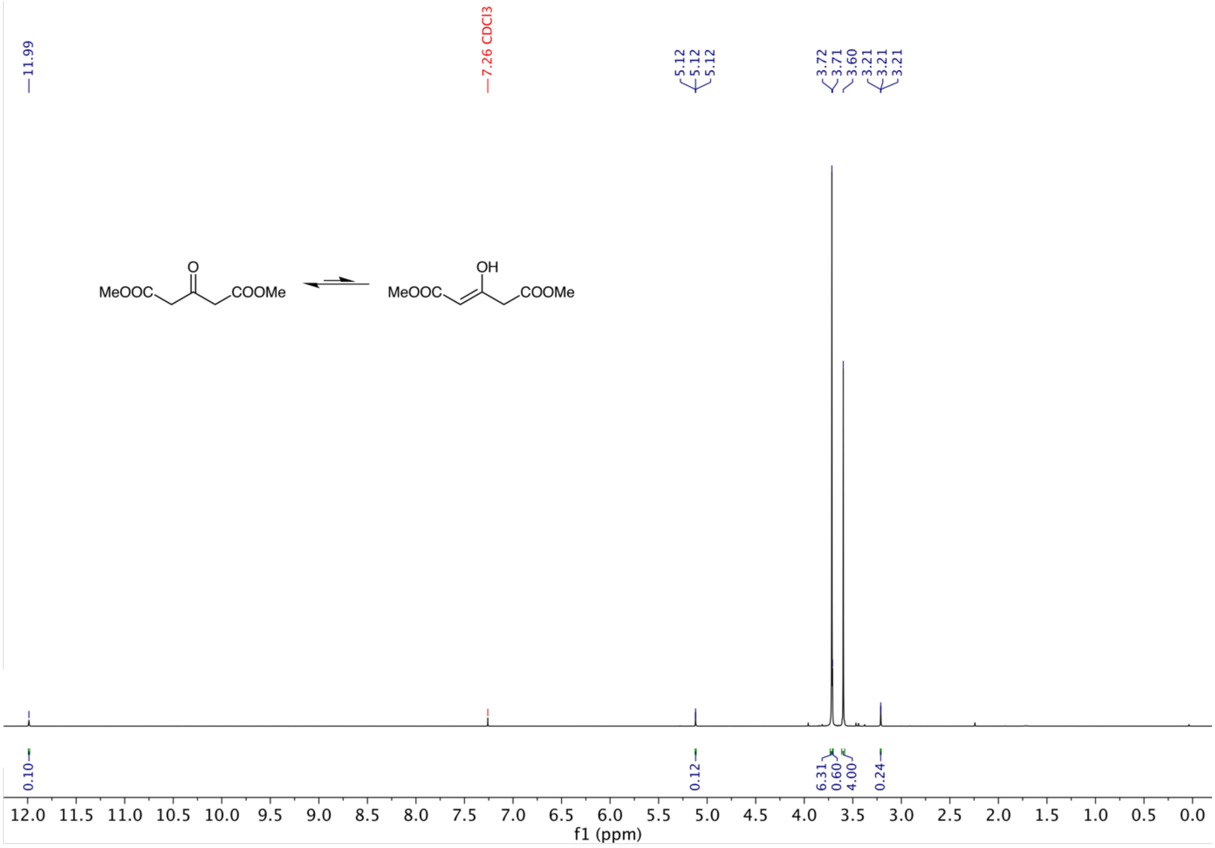

^{1}H-NMR (300.1 MHz, $CDCl_3$): Dimethyl 3-oxopentanedioate (**17**).

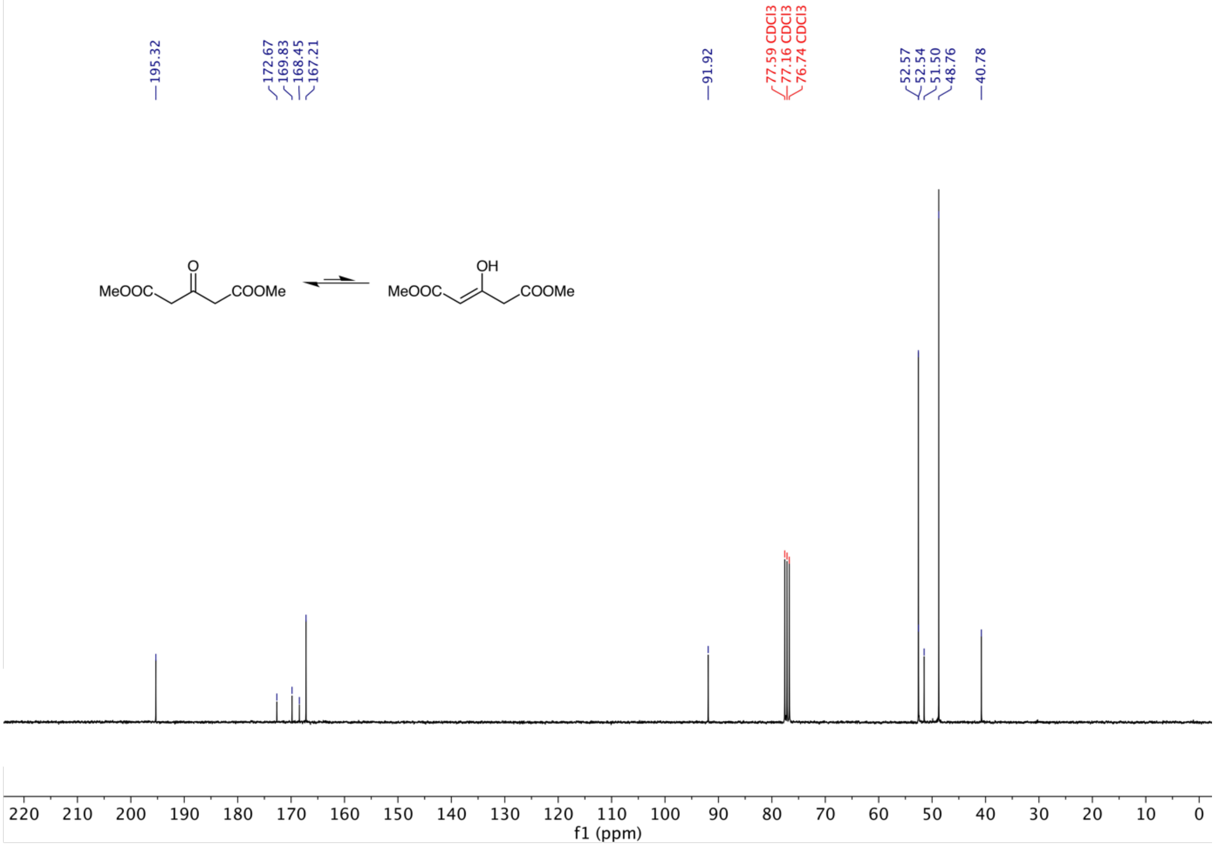

^{13}C-NMR (75.5 MHz, $CDCl_3$): Dimethyl 3-oxopentanedioate (**17**).

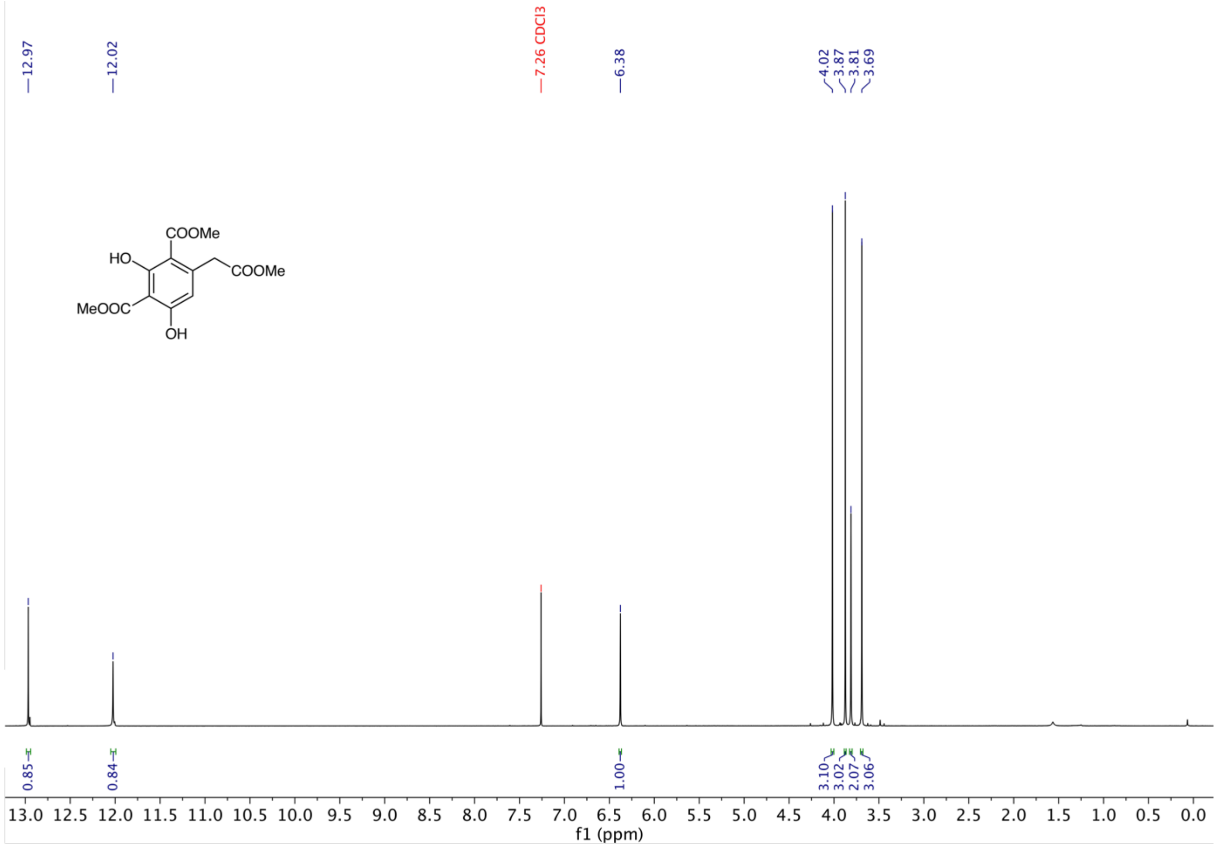

^{1}H-NMR (300.1 MHz, $CDCl_3$): Dimethyl 2,4-dihydroxy-6-(2-methoxy-2-oxoethyl)isophthalate (**18**).

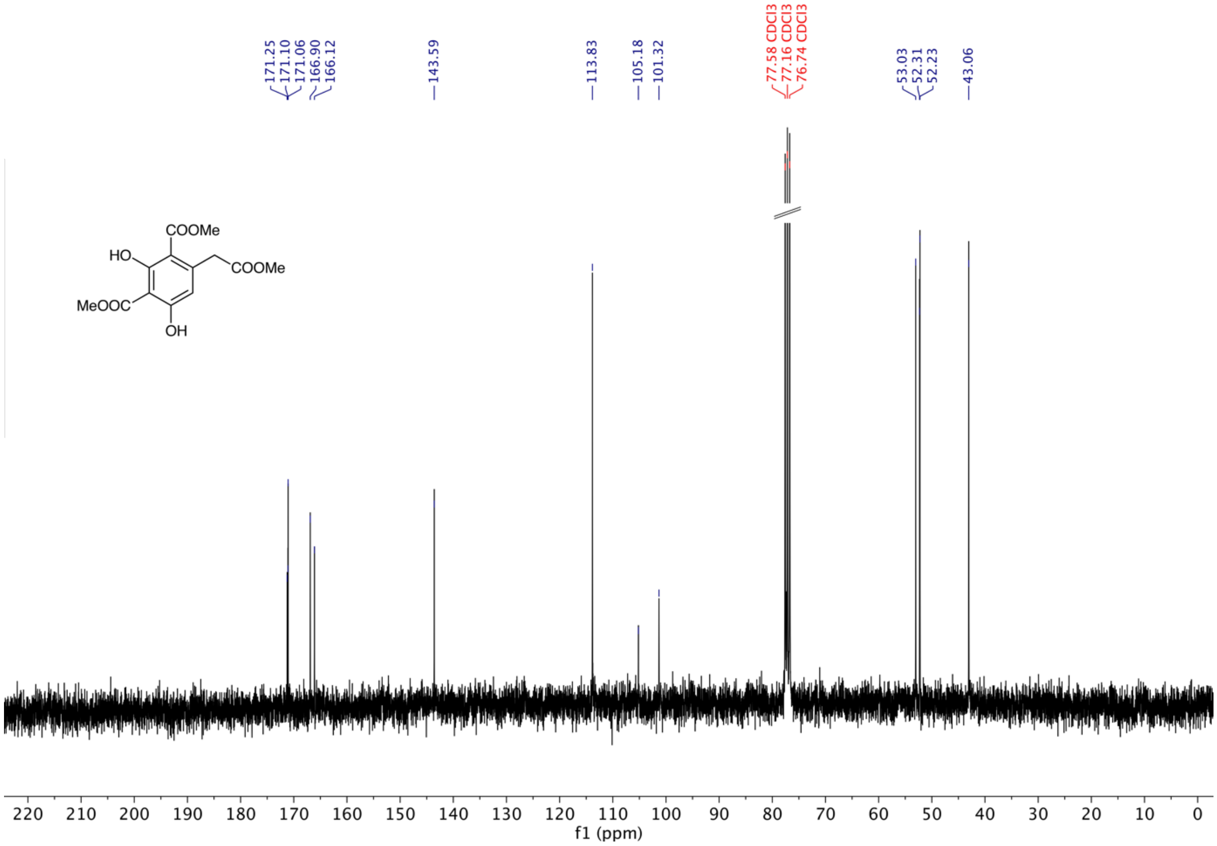

^{13}C-NMR (75.5 MHz, $CDCl_3$): Dimethyl 2,4-dihydroxy-6-(2-methoxy-2-oxoethyl)isophthalate (**18**).

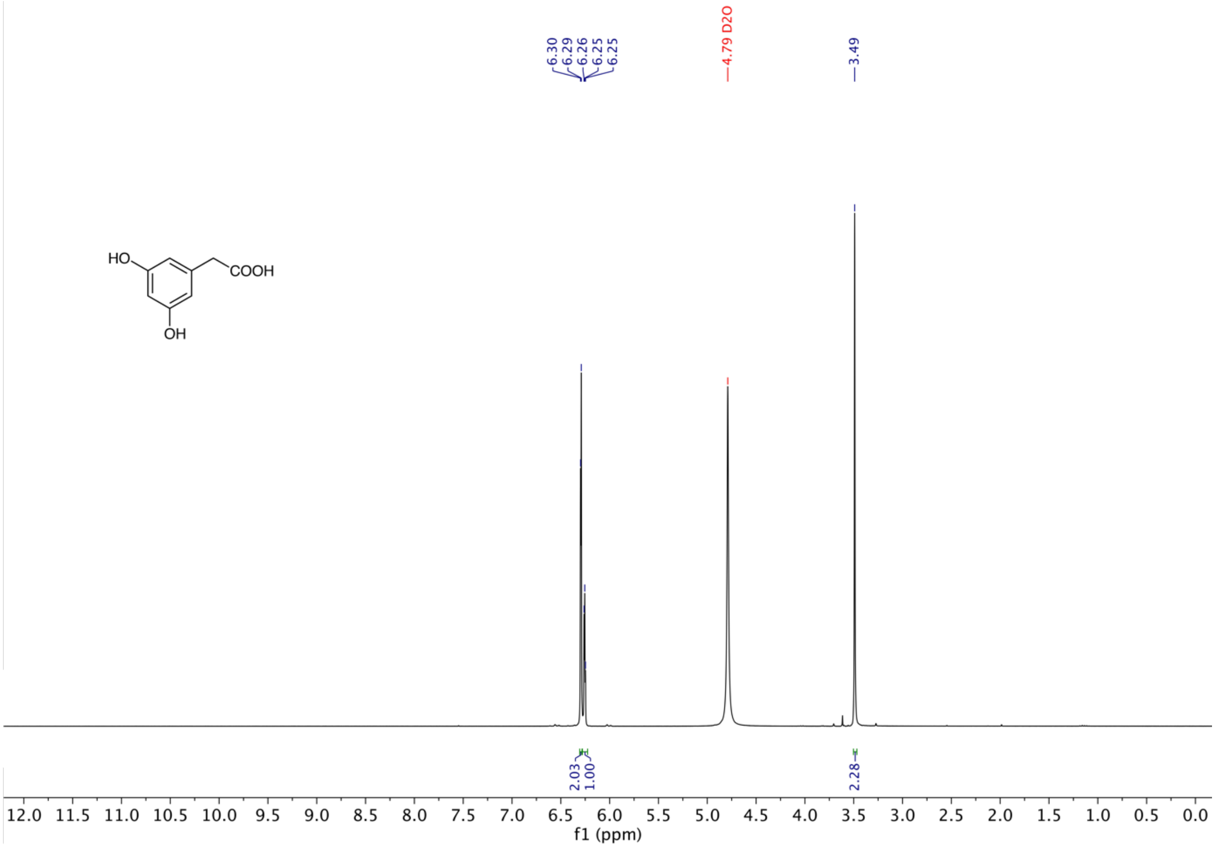

^{1}H-NMR (300.1 MHz, D_2O): 2-(3,5-Dihydroxyphenyl)acetic acid (**19**).

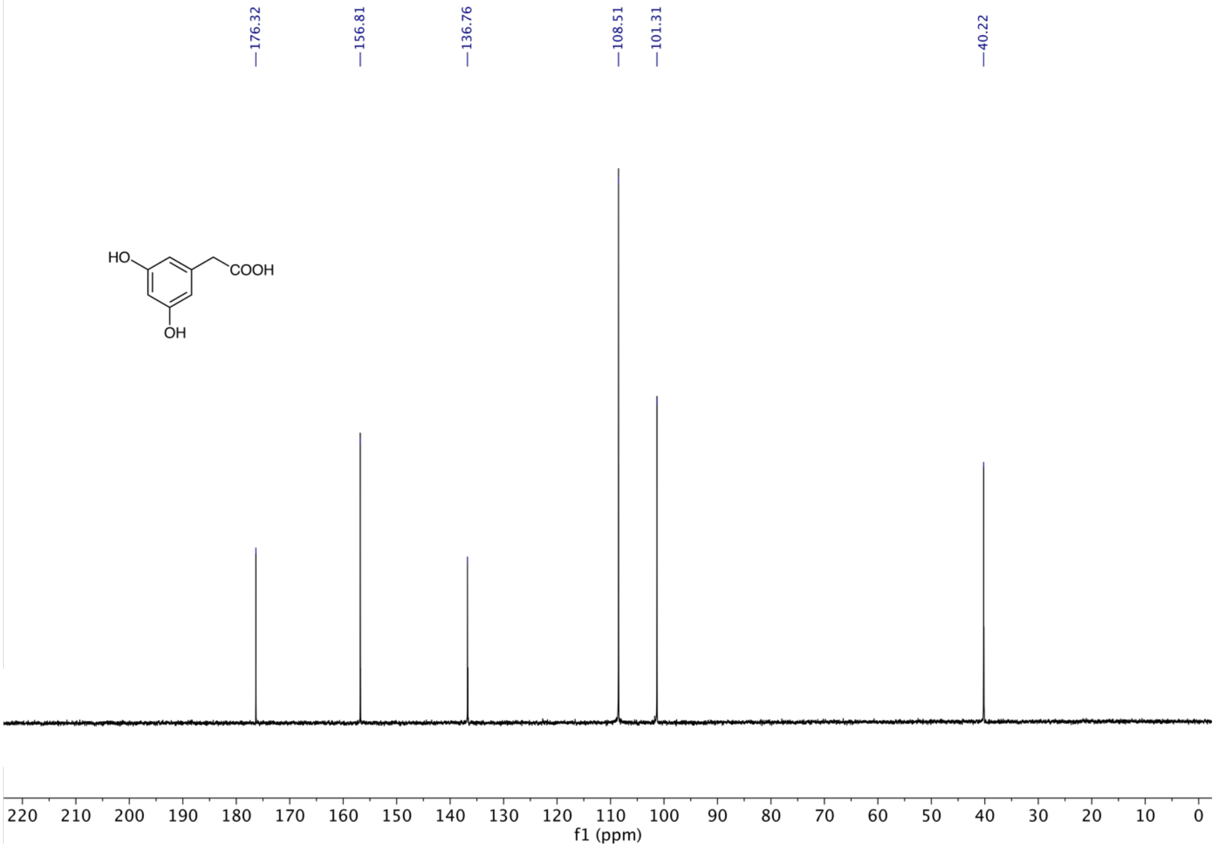

^{13}C-NMR (75.5 MHz, D_2O): 2-(3,5-Dihydroxyphenyl)acetic acid (**19**).

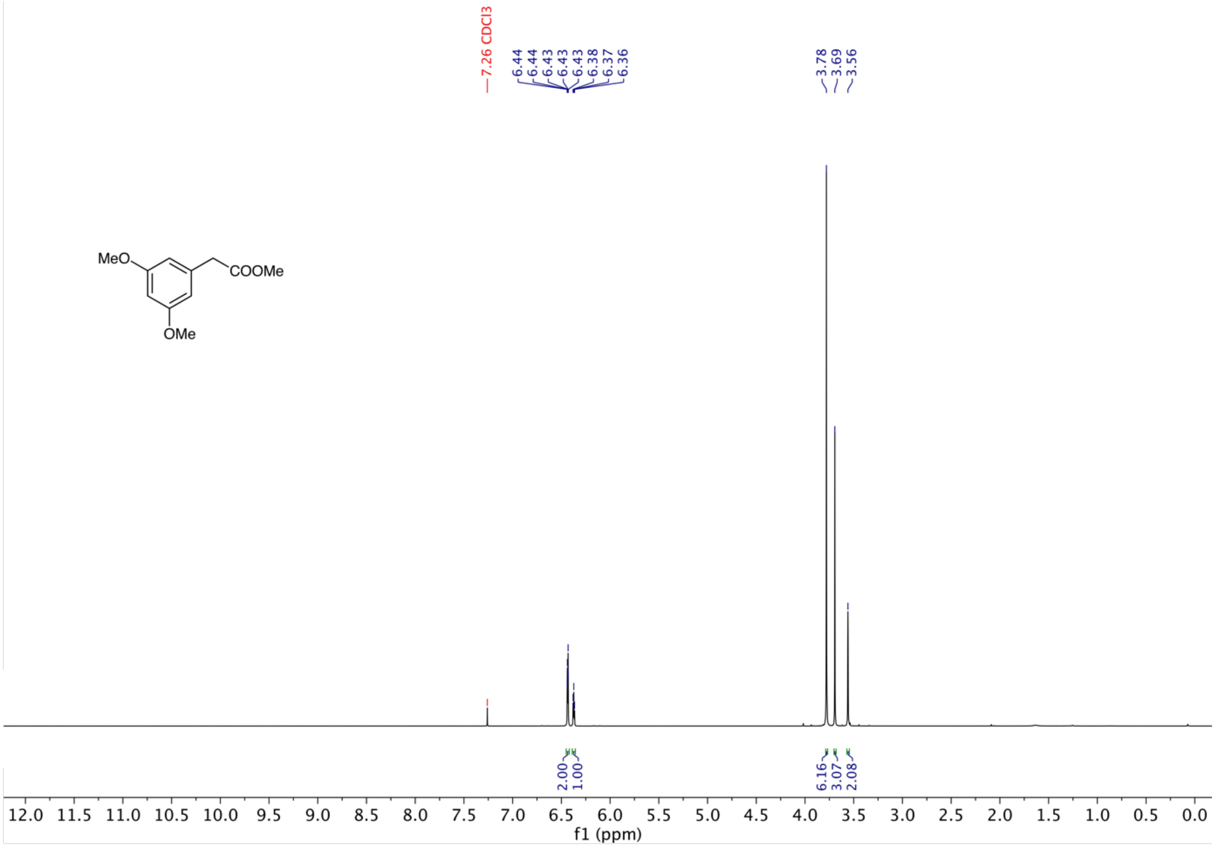

^{1}H-NMR (300.1 MHz, $CDCl_3$): Methyl 2-(3,5-Dimethoxyphenyl)acetate (**20**).

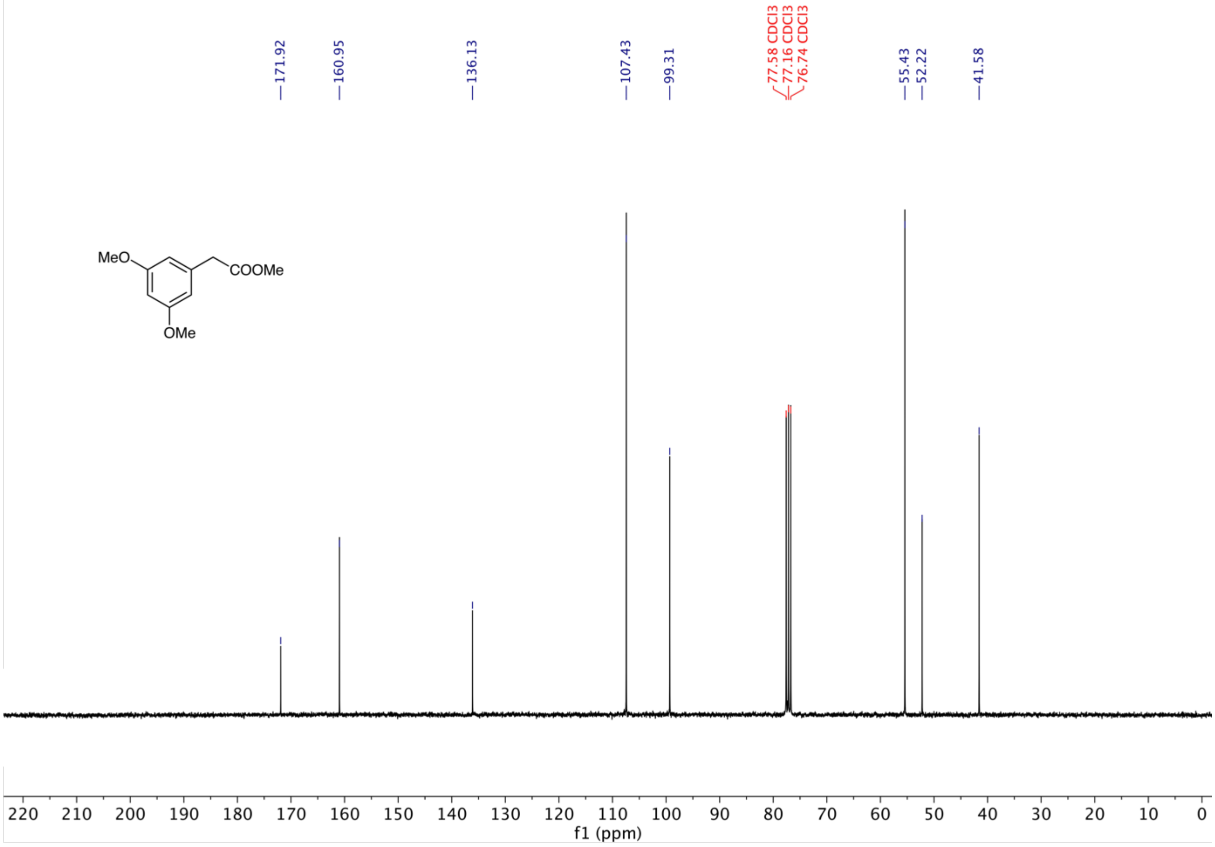

^{13}C-NMR (75.5 MHz, $CDCl_3$): Methyl 2-(3,5-Dimethoxyphenyl)acetate (**20**).

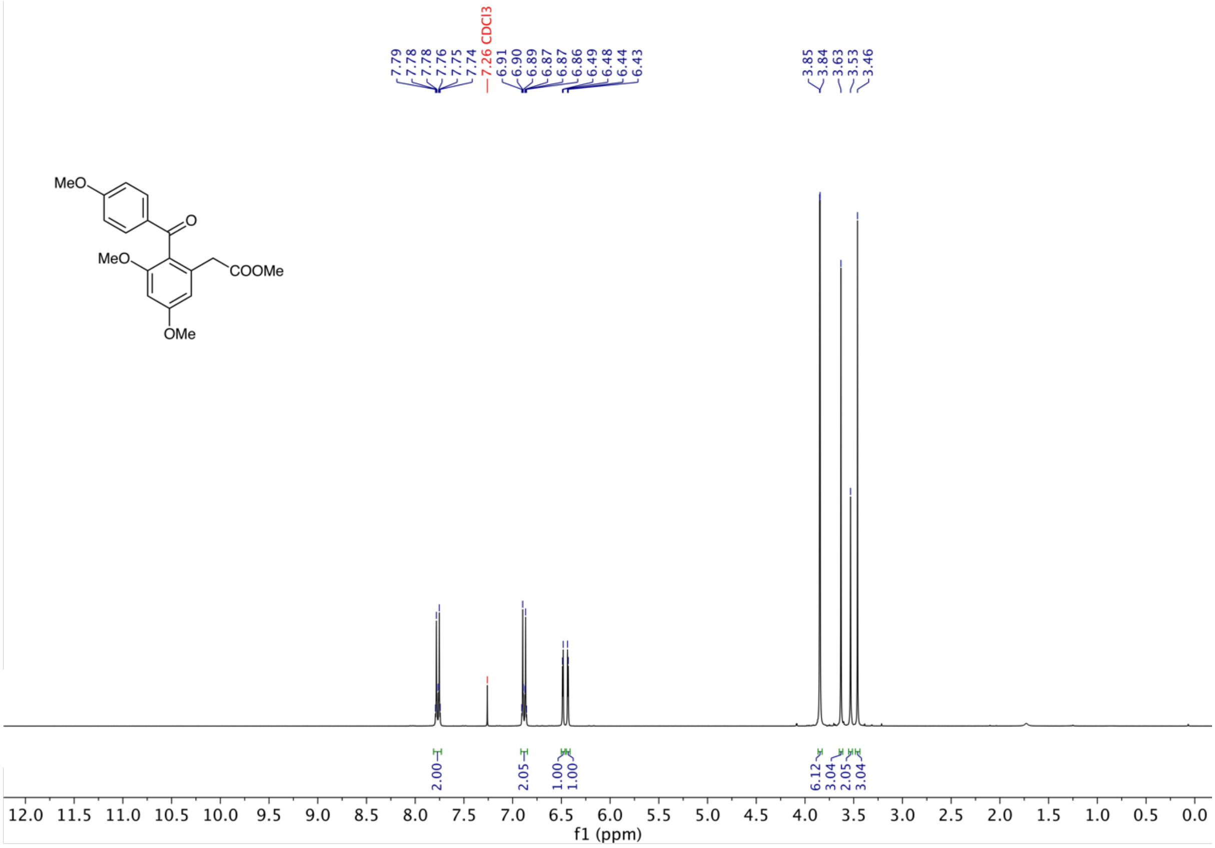

^{1}H-NMR (300.1 MHz, $CDCl_3$): Methyl 2-(3,5-dimethoxy-2-(4-methoxybenzoyl)phenyl)acetate (**21**).

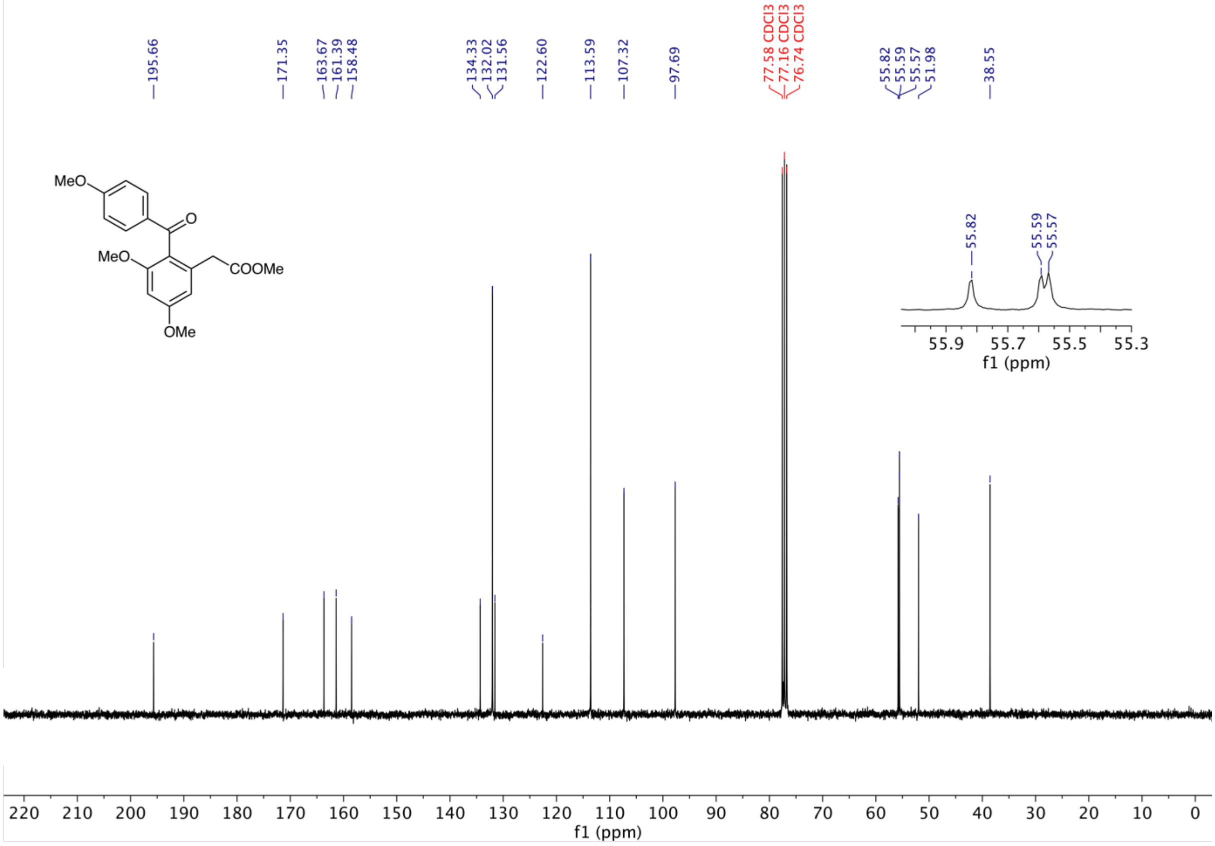

^{13}C-NMR (75.5 MHz, $CDCl_3$): Methyl 2-(3,5-dimethoxy-2-(4-methoxybenzoyl)phenyl)acetate (**21**).

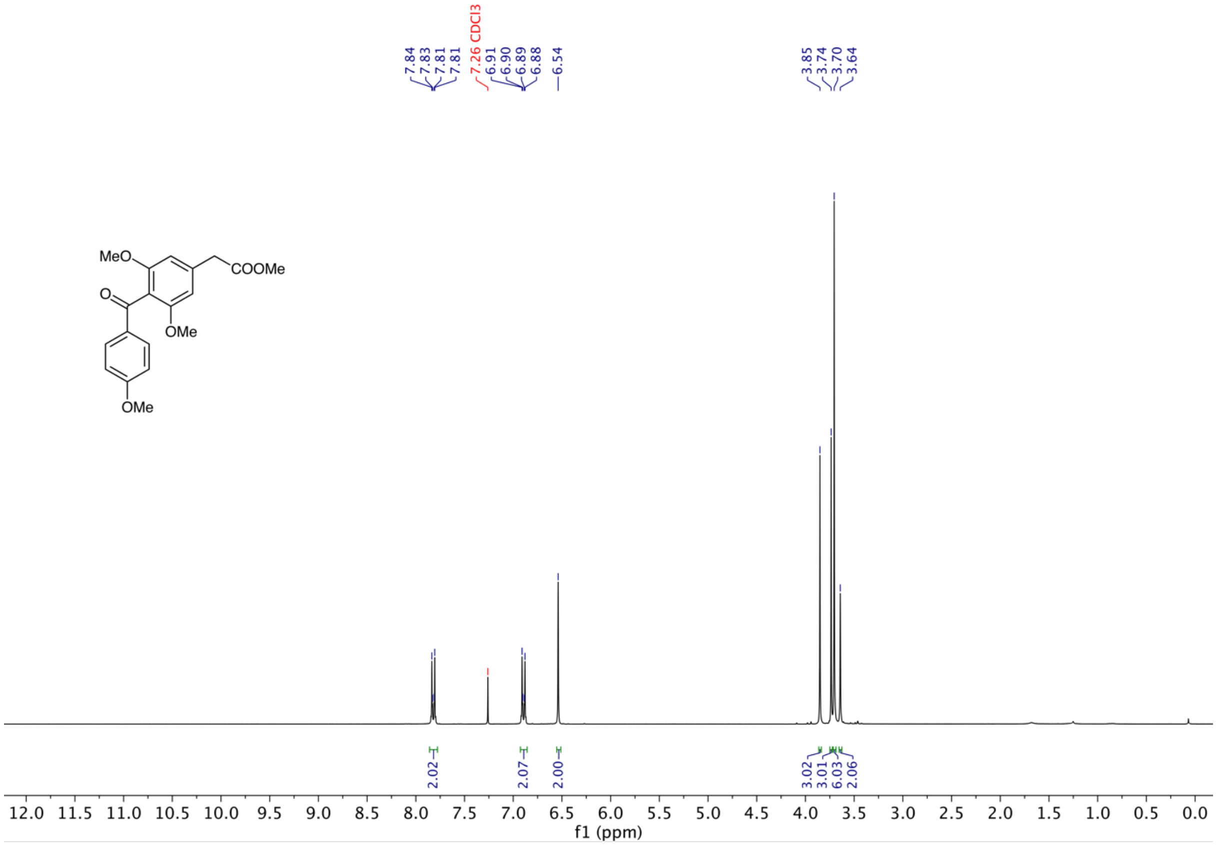

^{1}H-NMR (300.1 MHz, $CDCl_3$): Methyl 2-(3,5-dimethoxy-4-(4-methoxybenzoyl)phenyl)acetate (**21a**).

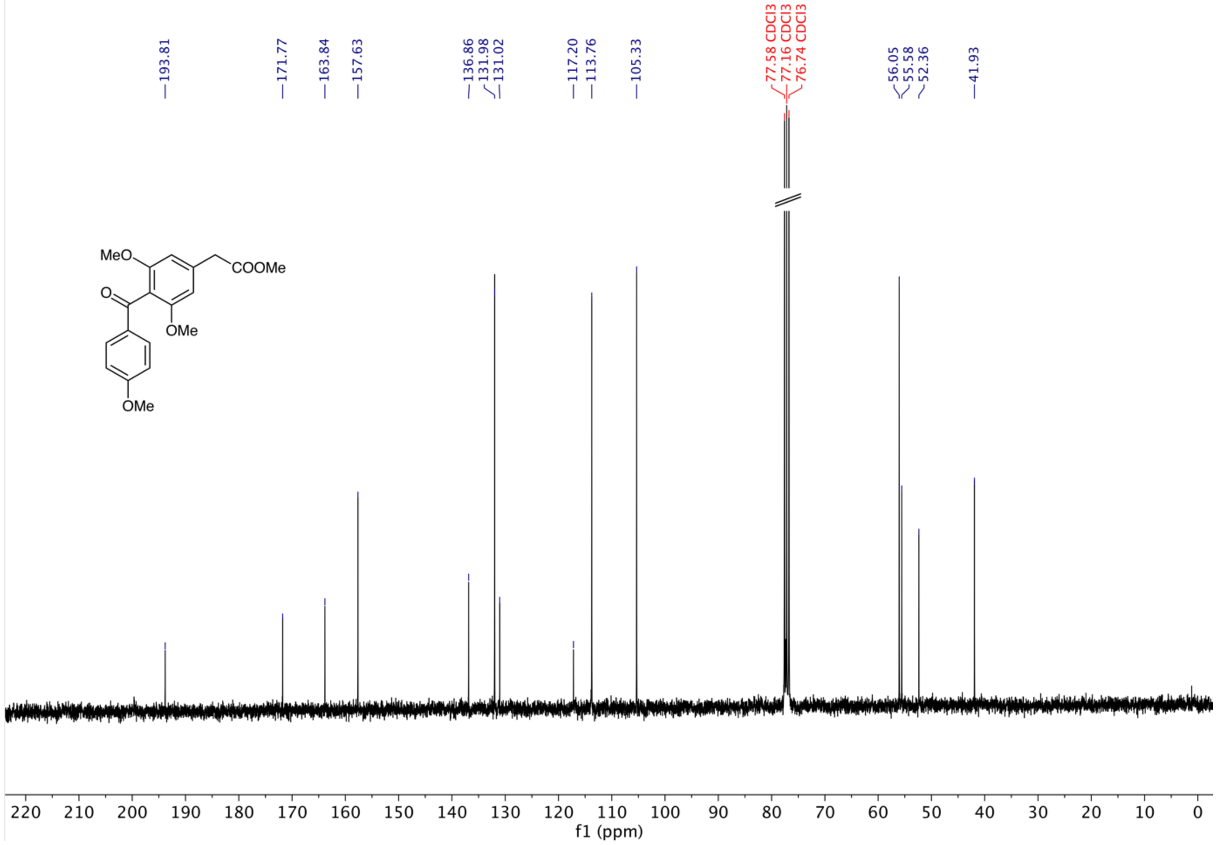

^{13}C-NMR (75.5 MHz, $CDCl_3$): Methyl 2-(3,5-dimethoxy-4-(4-methoxybenzoyl)phenyl)acetate (**21a**).

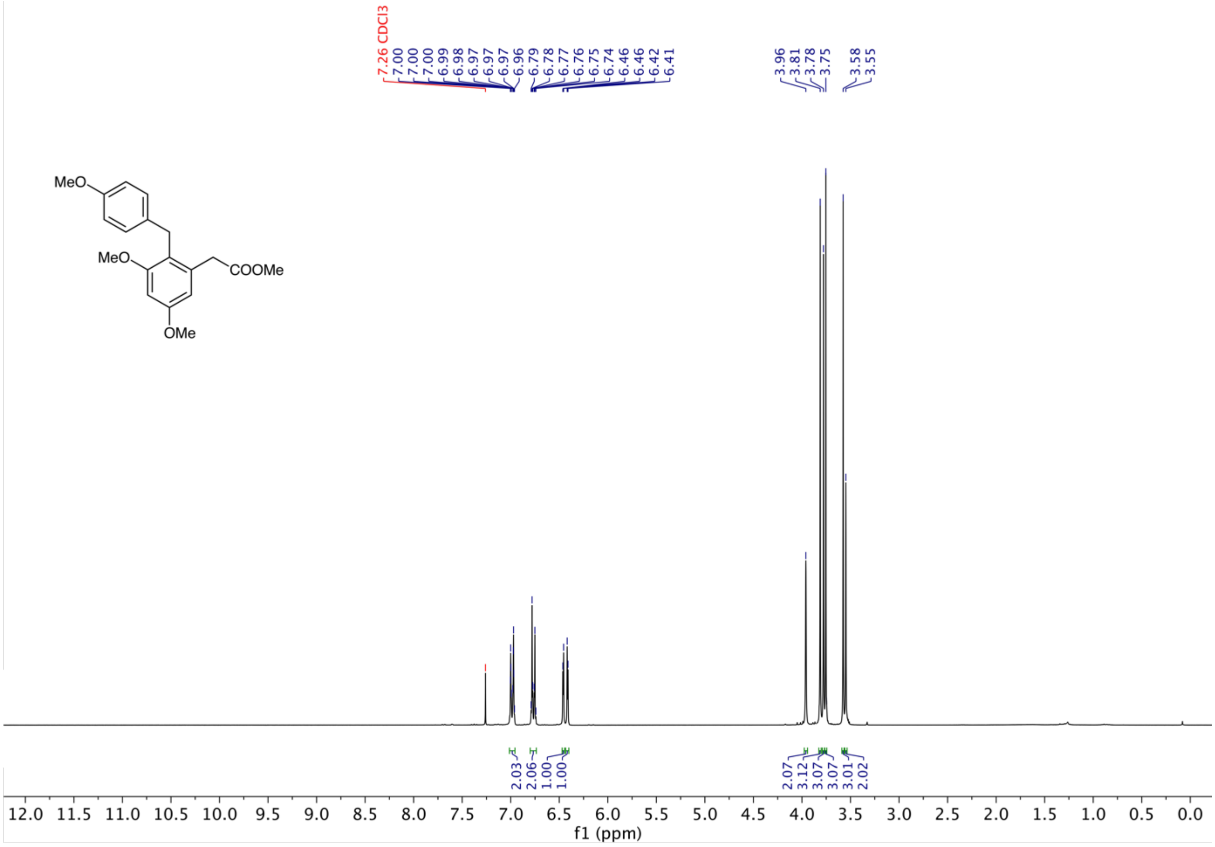

^{1}H-NMR (300.1 MHz, $CDCl_3$): Methyl 2-(3,5-dimethoxy-2-(4-methoxybenzyl)phenyl)acetate (**22**).

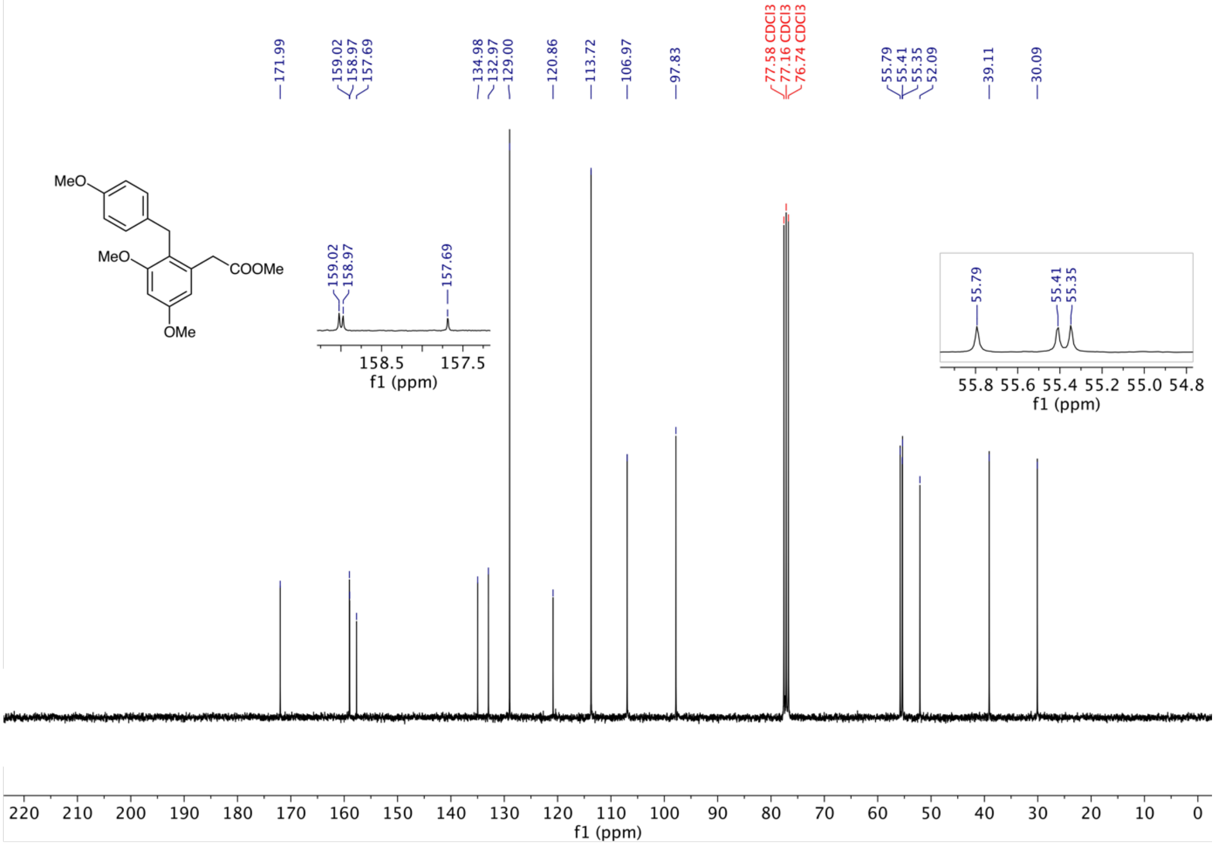

^{13}C-NMR (75.5 MHz, $CDCl_3$): Methyl 2-(3,5-dimethoxy-2-(4-methoxybenzyl)phenyl)acetate (**22**).

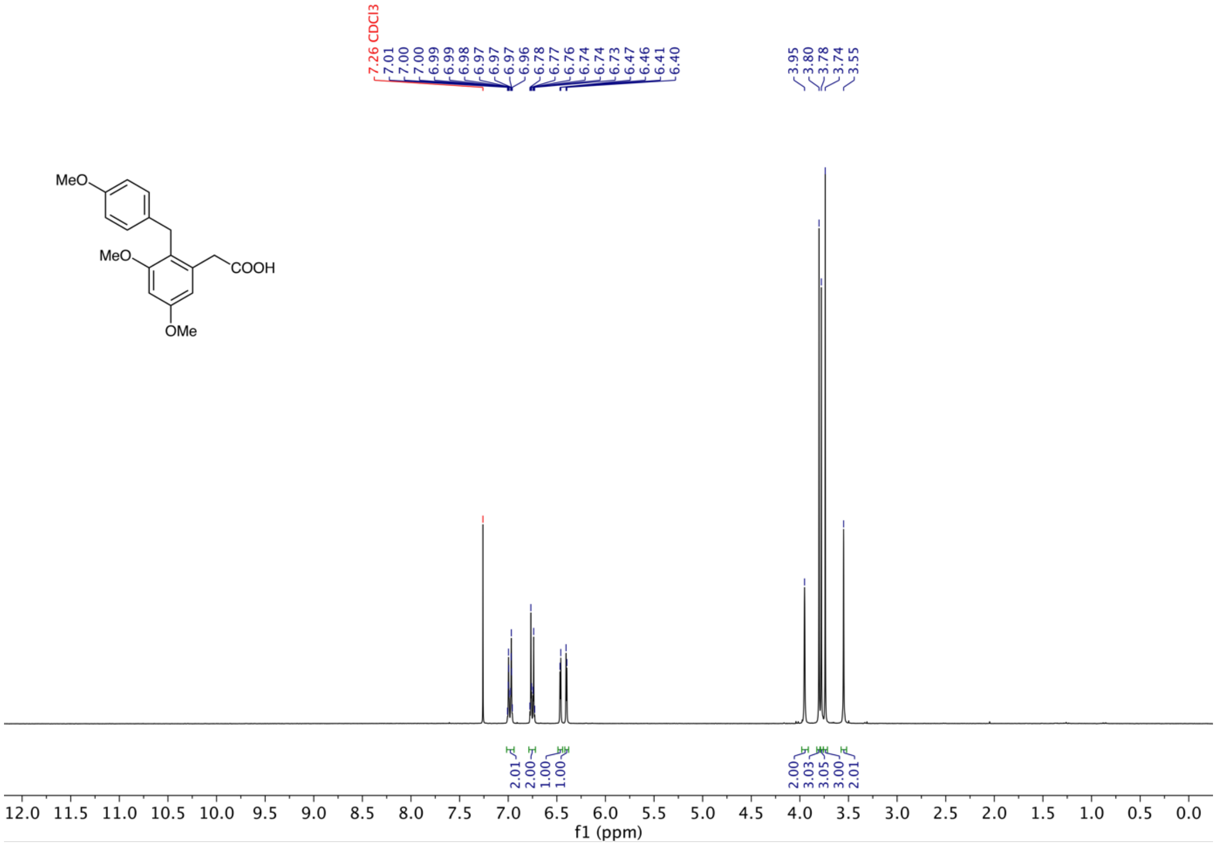

^{1}H-NMR (300.1 MHz, $CDCl_3$): 2-(3,5-Dimethoxy-2-(4-methoxybenzyl)phenyl)acetic acid (**12**).

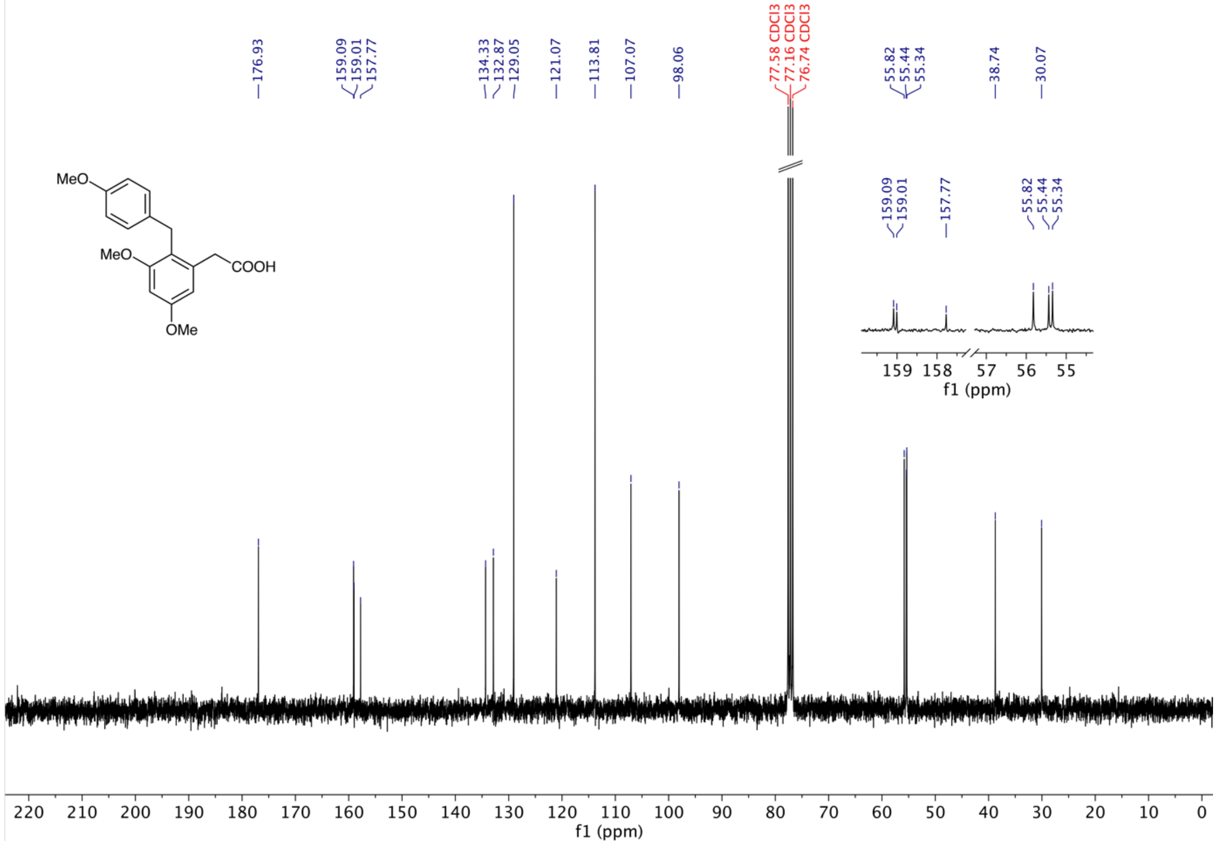

^{13}C-NMR (75.5 MHz, $CDCl_3$): 2-(3,5-Dimethoxy-2-(4-methoxybenzyl)phenyl)acetic acid (**12**).

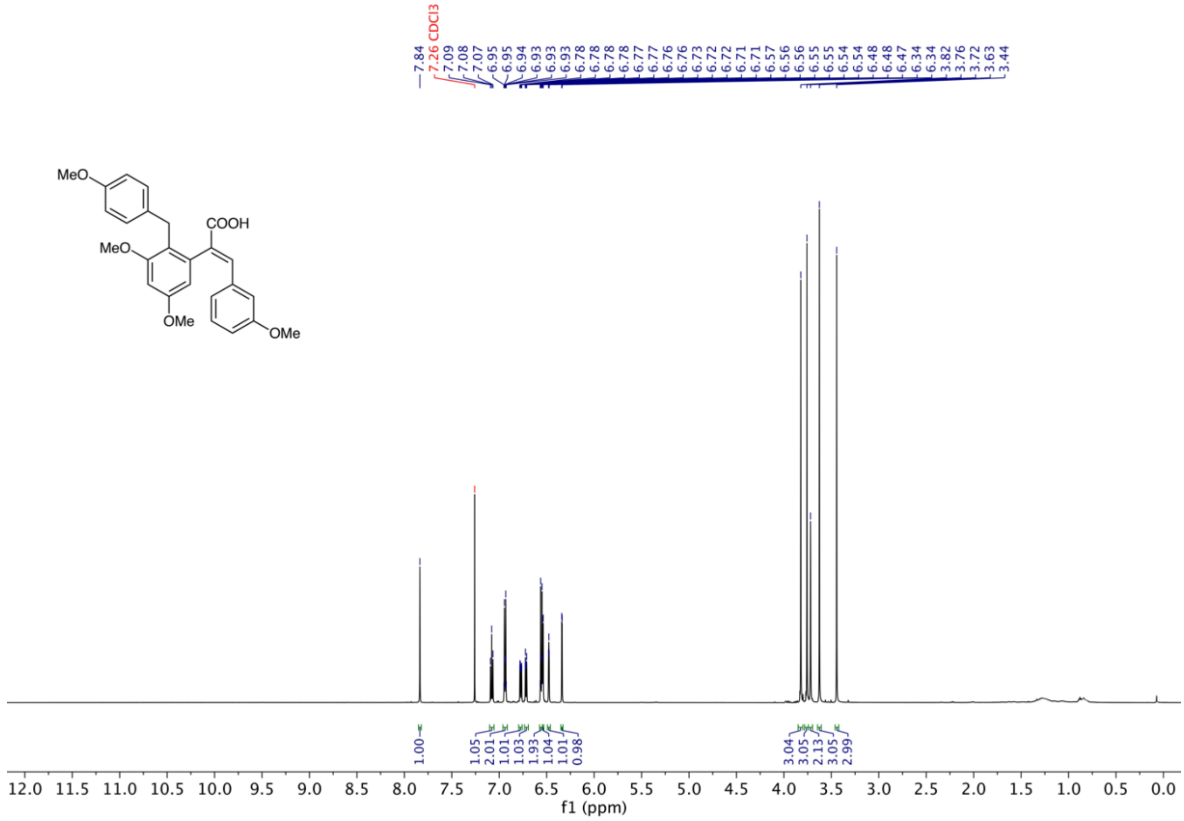

^{1}H-NMR (600.1 MHz, $CDCl_3$): (*E*)-2-(3,5-Dimethoxy-2-(4-methoxybenzyl)phenyl)-3-(3-methoxyphenyl)prop-2-enoic acid (**23**).

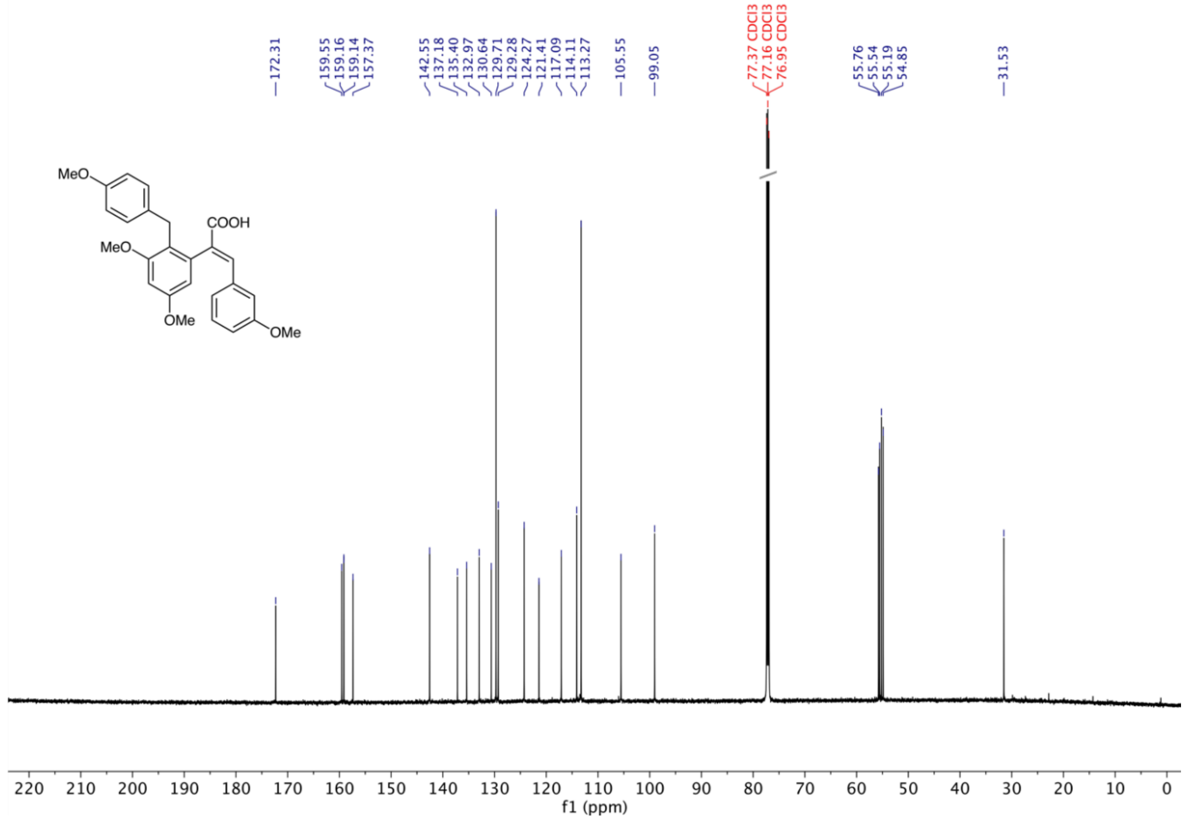

^{13}C-NMR (150.9 MHz, $CDCl_3$): (*E*)-2-(3,5-Dimethoxy-2-(4-methoxybenzyl)phenyl)-3-(3-methoxyphenyl)prop-2-enoic acid (**23**).

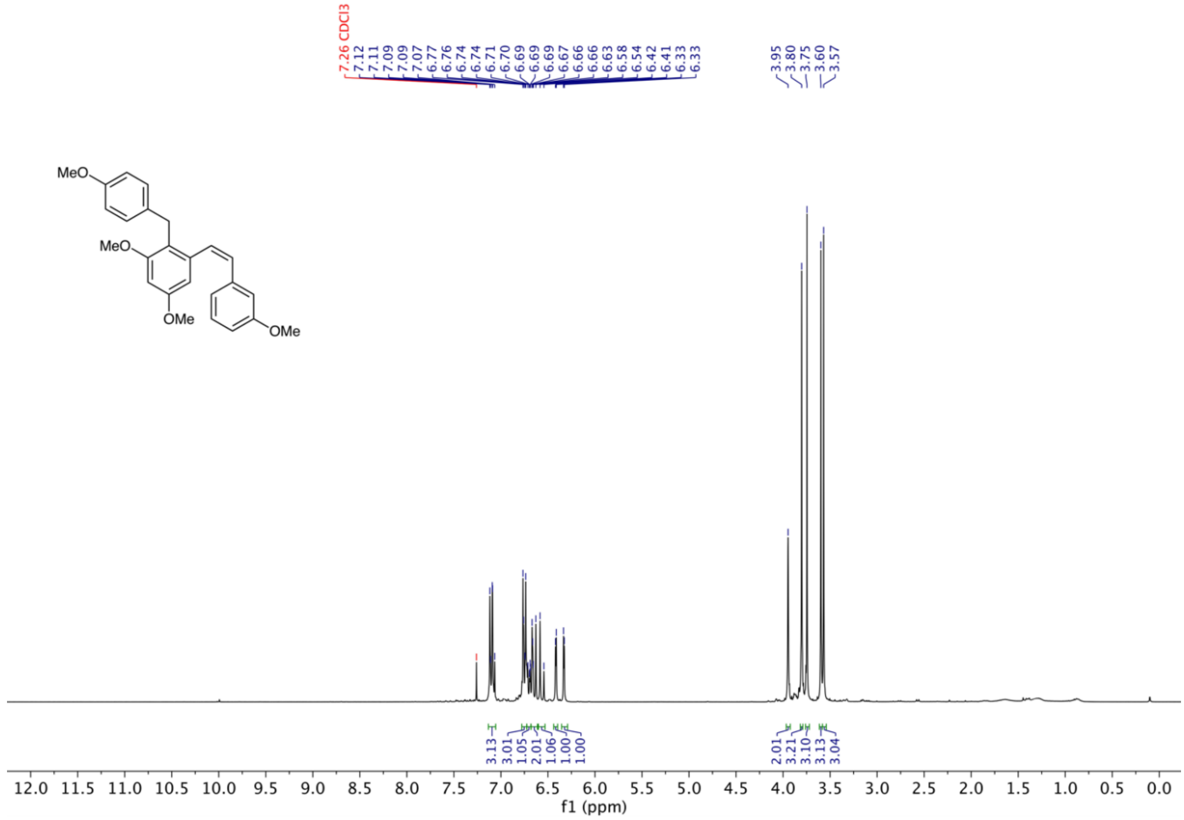

^{1}H-NMR (300.1 MHz, $CDCl_3$): 1,5-Dimethoxy-2-(4-methoxybenzyl)-3-((*Z*)-2-(3-methoxyphenyl)ethenyl)benzene (*cis*-**24**).

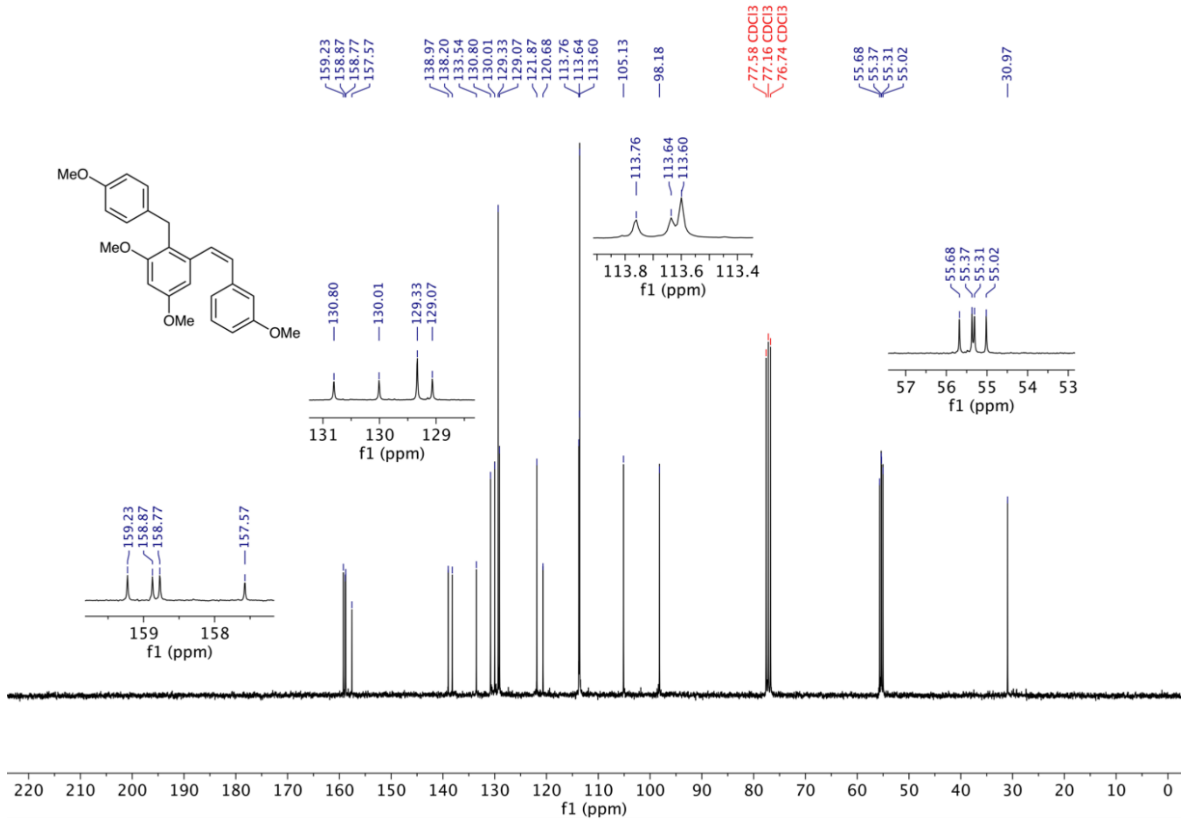

^{13}C-NMR (75.5 MHz, $CDCl_3$): 1,5-Dimethoxy-2-(4-methoxybenzyl)-3-((*Z*)-2-(3-methoxyphenyl)ethenyl)benzene (*cis*-**24**).

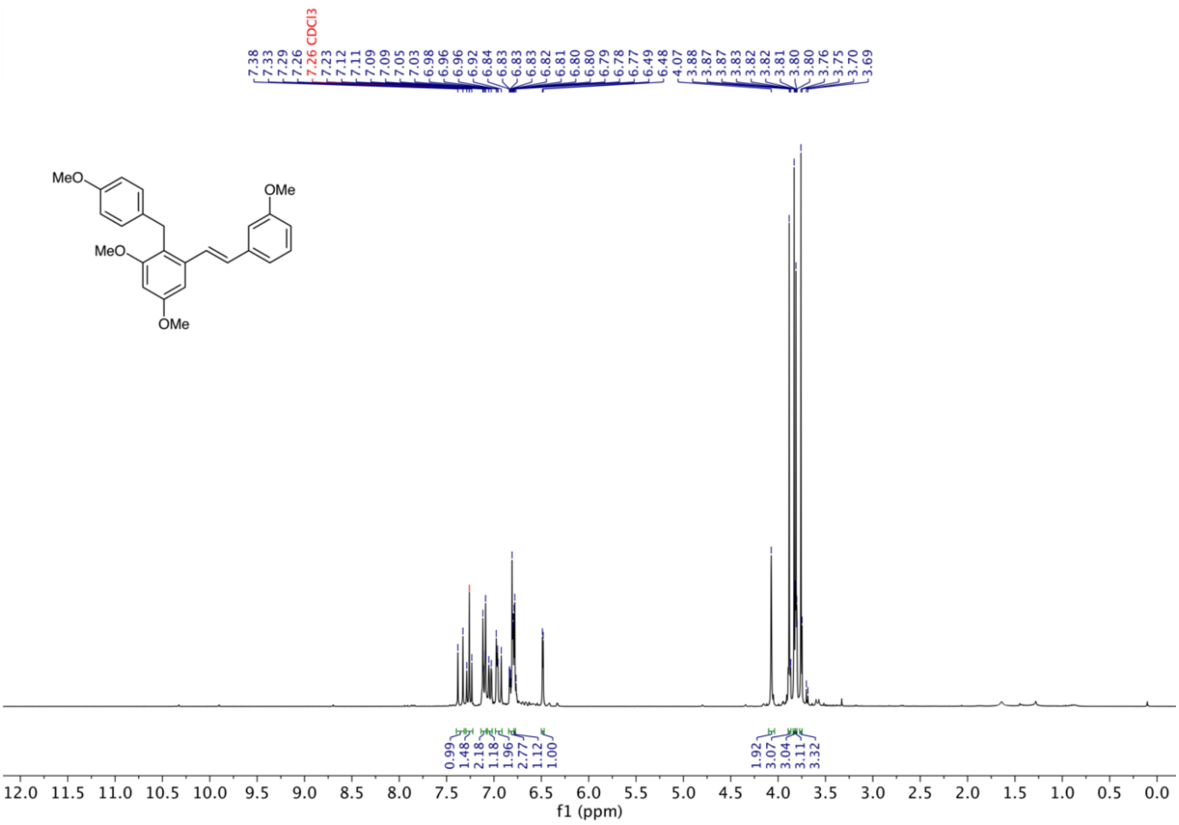

^{1}H-NMR (300.1 MHz, $CDCl_3$): 1,5-Dimethoxy-2-(4-methoxybenzyl)-3-((*E*)-2-(3-methoxyphenyl)ethenyl)benzene (*trans*-**24**).

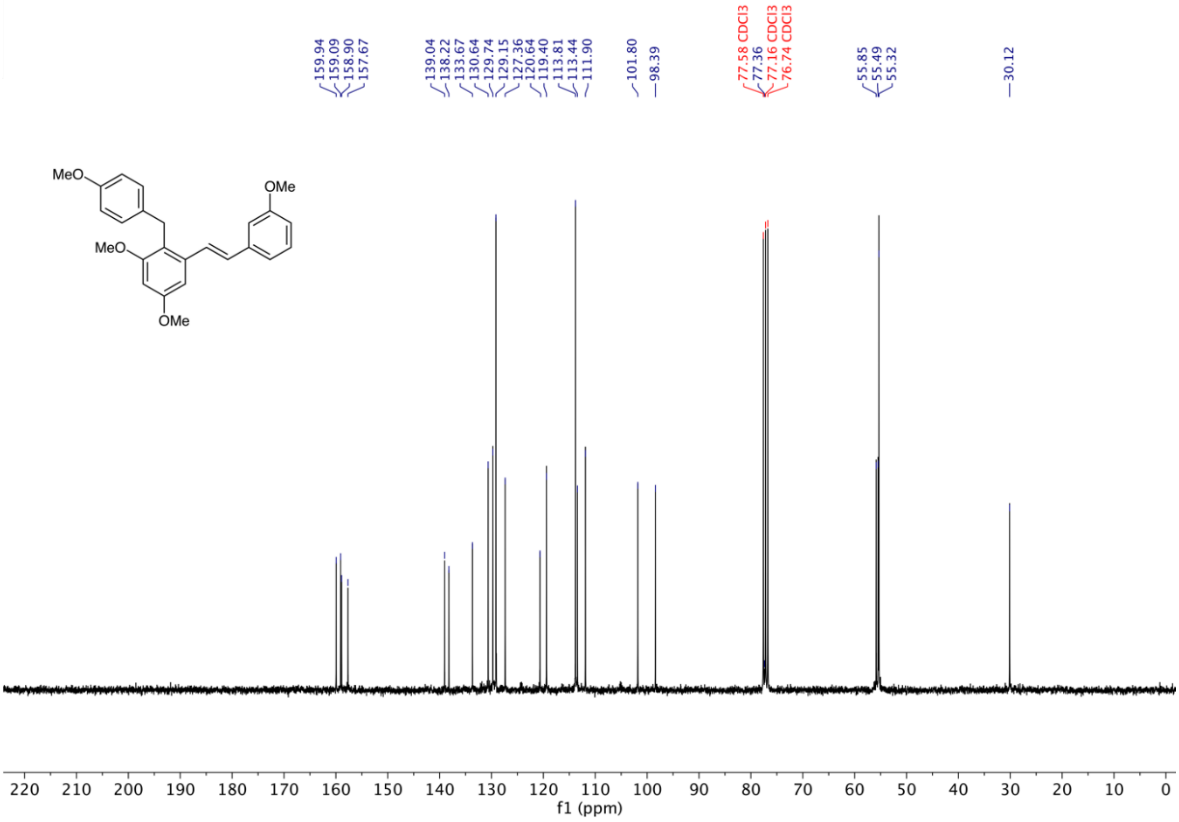

^{13}C-NMR (75.5 MHz, $CDCl_3$): 1,5-Dimethoxy-2-(4-methoxybenzyl)-3-((*E*)-2-(3-methoxyphenyl)ethenyl)benzene (*trans*-**24**).

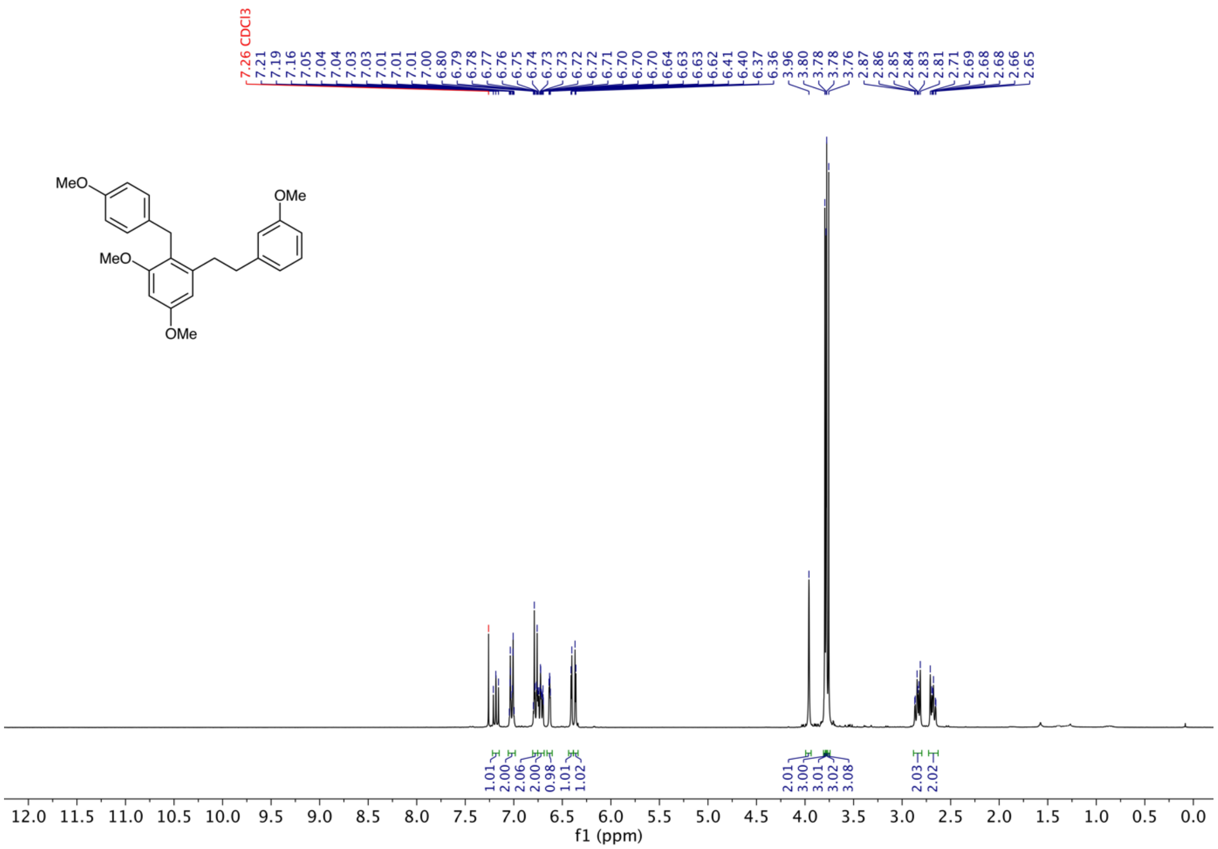

^{1}H-NMR (300.1 MHz, $CDCl_3$): 1,5-Dimethoxy-2-(4-methoxybenzyl)-3-(2-(3-methoxyphenyl)ethyl)benzene (**9**).

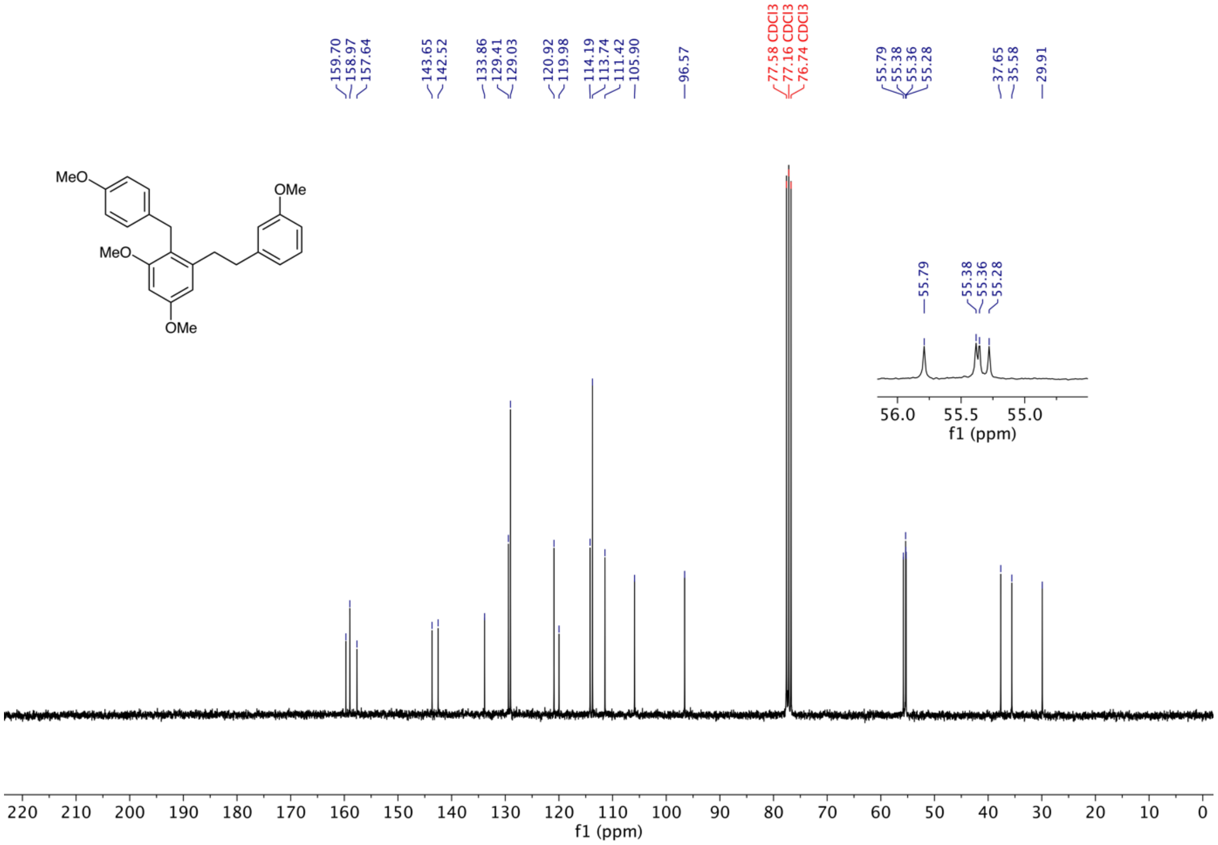

^{13}C-NMR (75.5 MHz, $CDCl_3$): 1,5-Dimethoxy-2-(4-methoxybenzyl)-3-(2-(3-methoxyphenyl)ethyl)benzene (**9**).

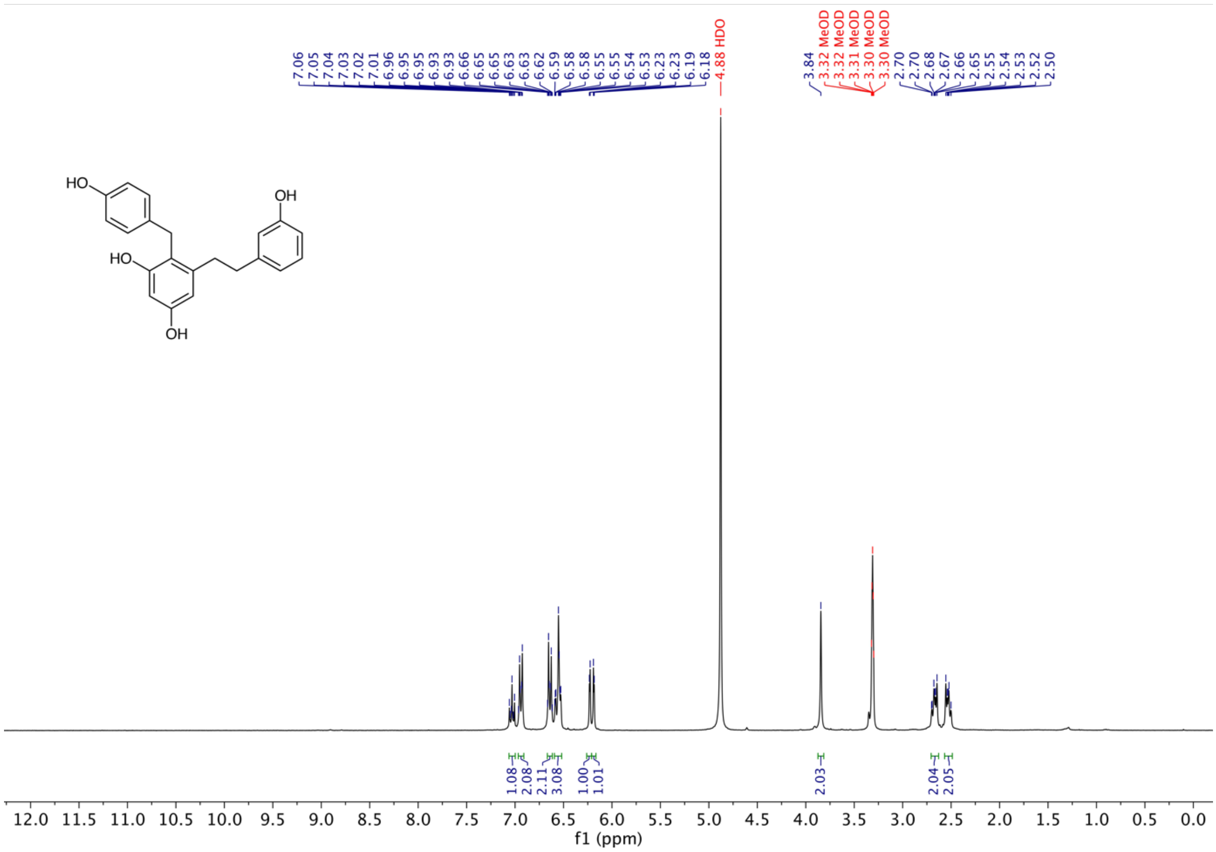

^{1}H-NMR (300.1 MHz, MeOD): 4-(4-Hydroxybenzyl)-5-(2-(3-hydroxyphenyl)ethyl)benzene-1,3-diol (shancigusin C, **1**).

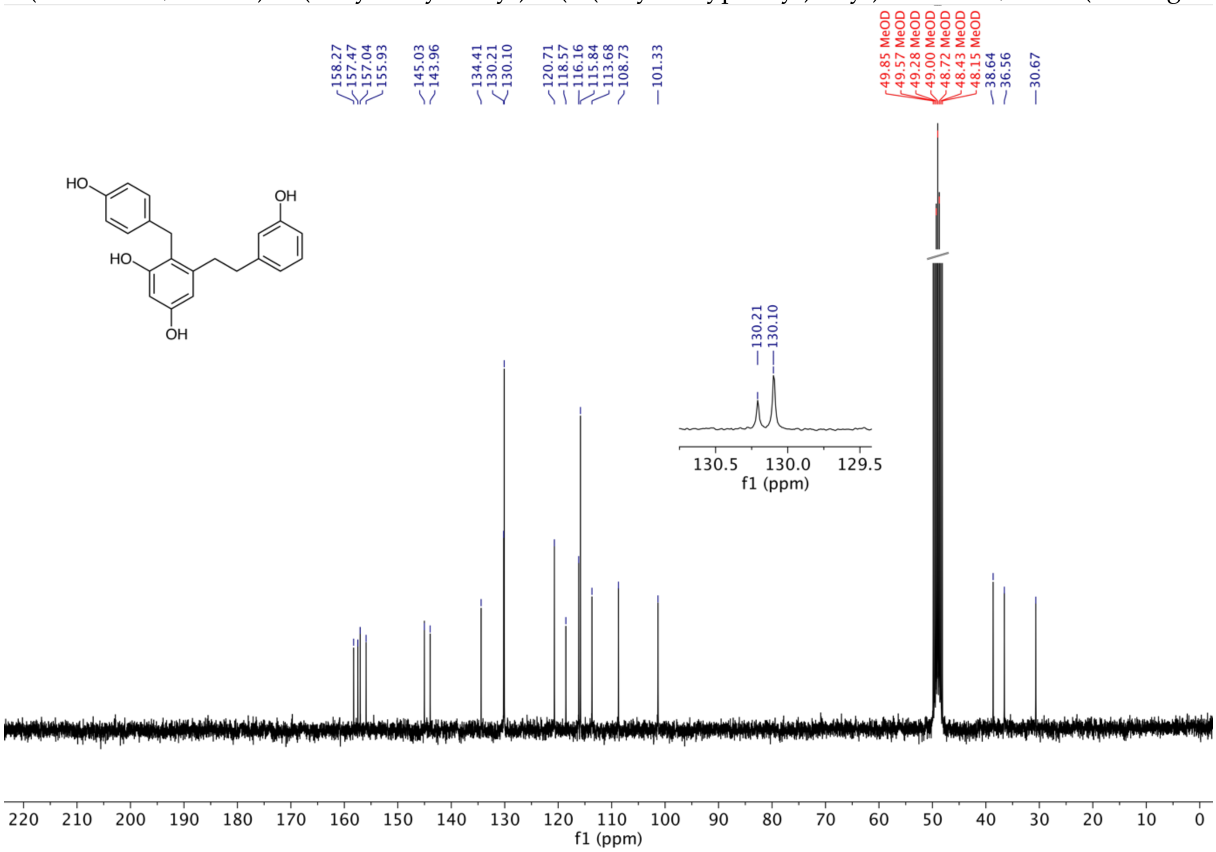

^{13}C-NMR (75.5 MHz, MeOD): 4-(4-Hydroxybenzyl)-5-(2-(3-hydroxyphenyl)ethyl)benzene-1,3-diol (shancigusin C, **1**).

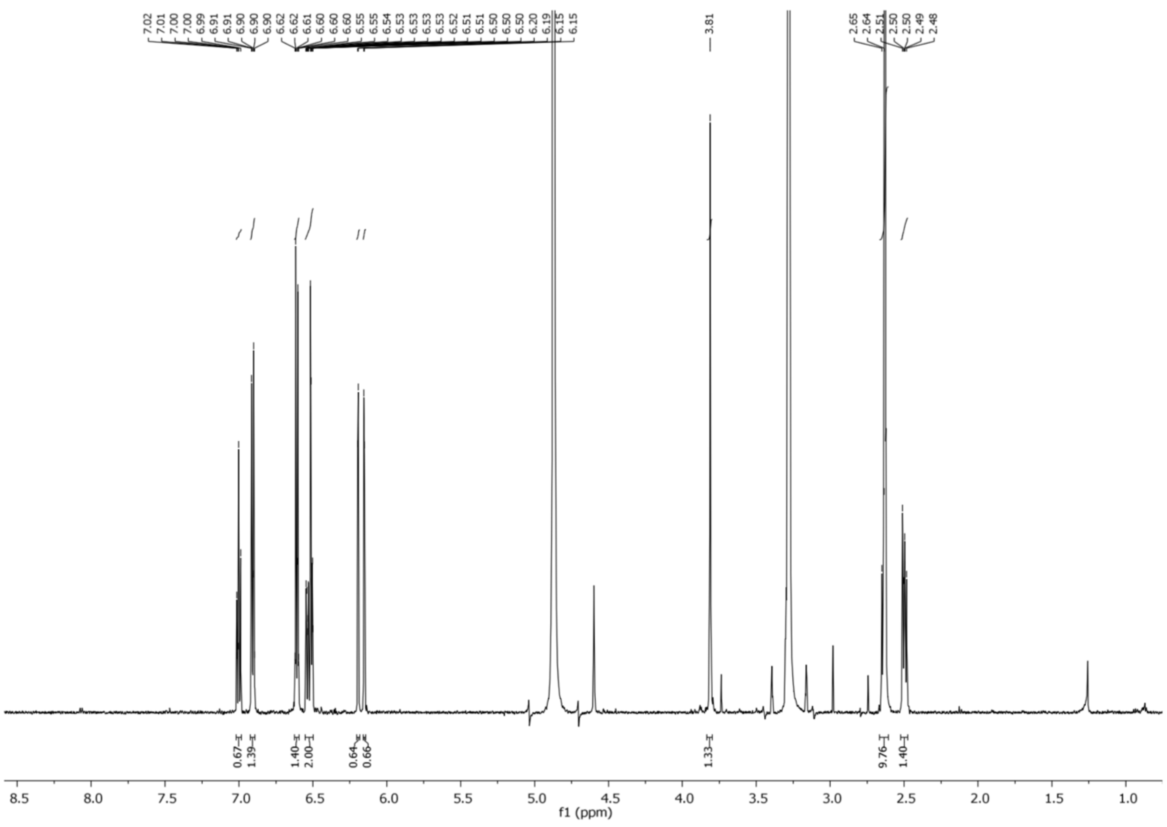

^{1}H-NMR (600.1 MHz, MeOD) of natural shancigusin C, **1**) [20].

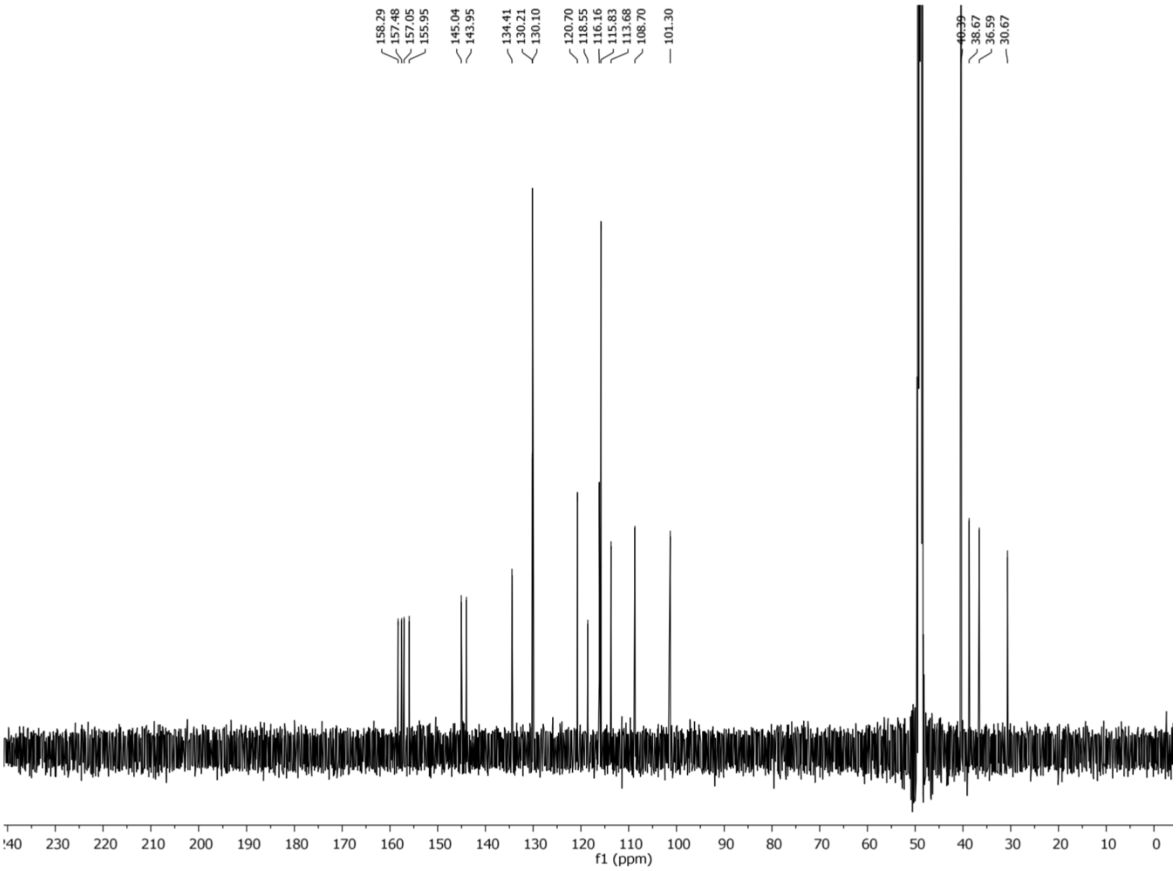

^{13}C-NMR (150.9 MHz, MeOD) of natural shancigusin C, **1**) [20].

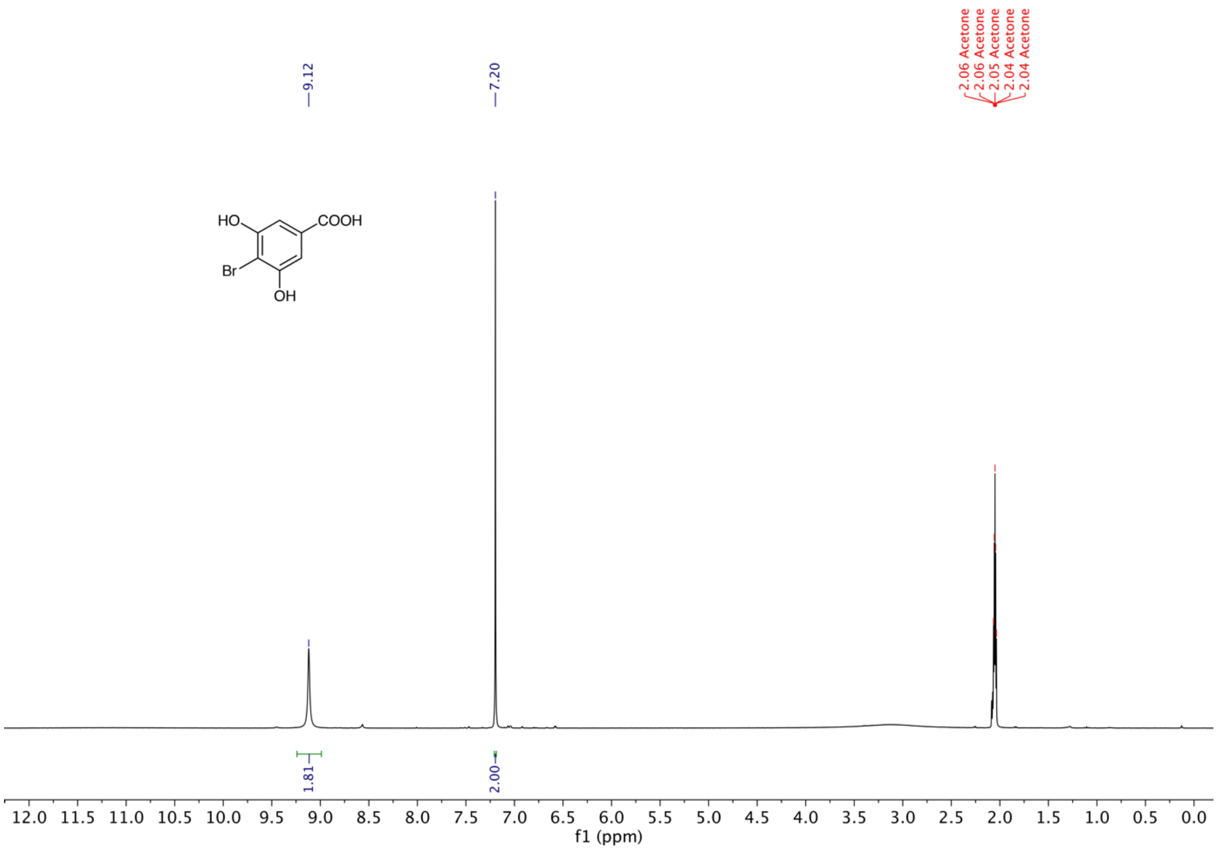

^{1}H-NMR (300.1 MHz, $(CD_3)_2CO$): 4-Bromo-3,5-dihydroxybenzoic acid (**30**).

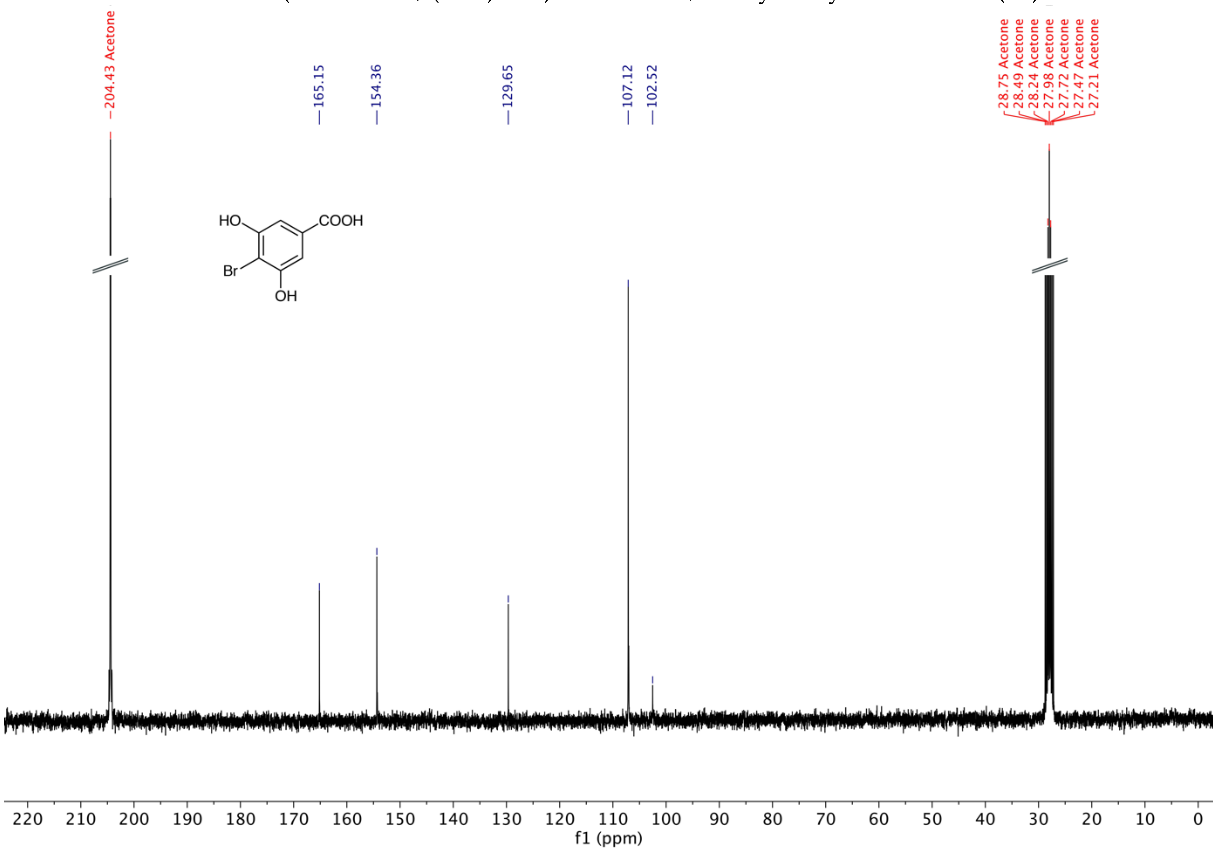

^{13}C-NMR (75.5 MHz, , $(CD_3)_2CO$): 4-Bromo-3,5-dihydroxybenzoic acid (**30**).

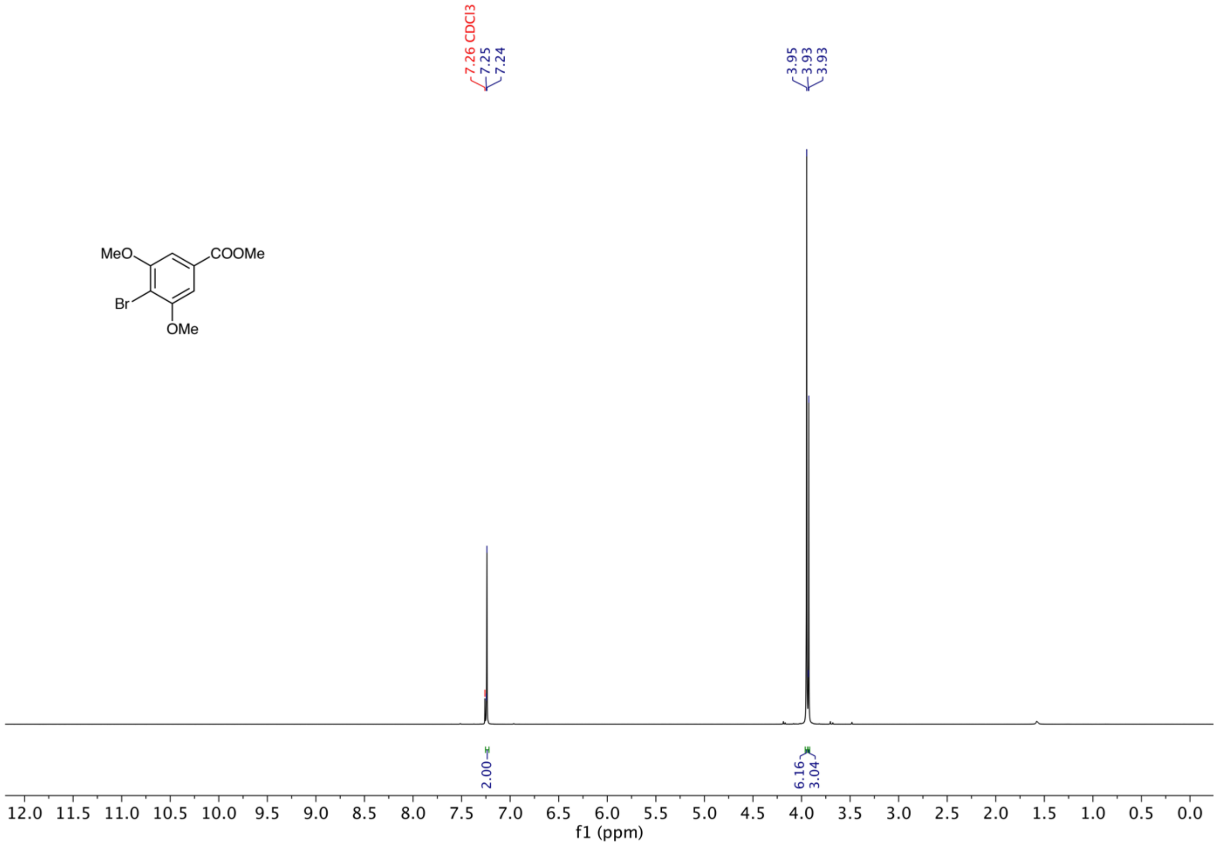

^{1}H-NMR (300.1 MHz, $CDCl_3$): Methyl 4-bromo-3,5-dimethoxybenzoate (**31**).

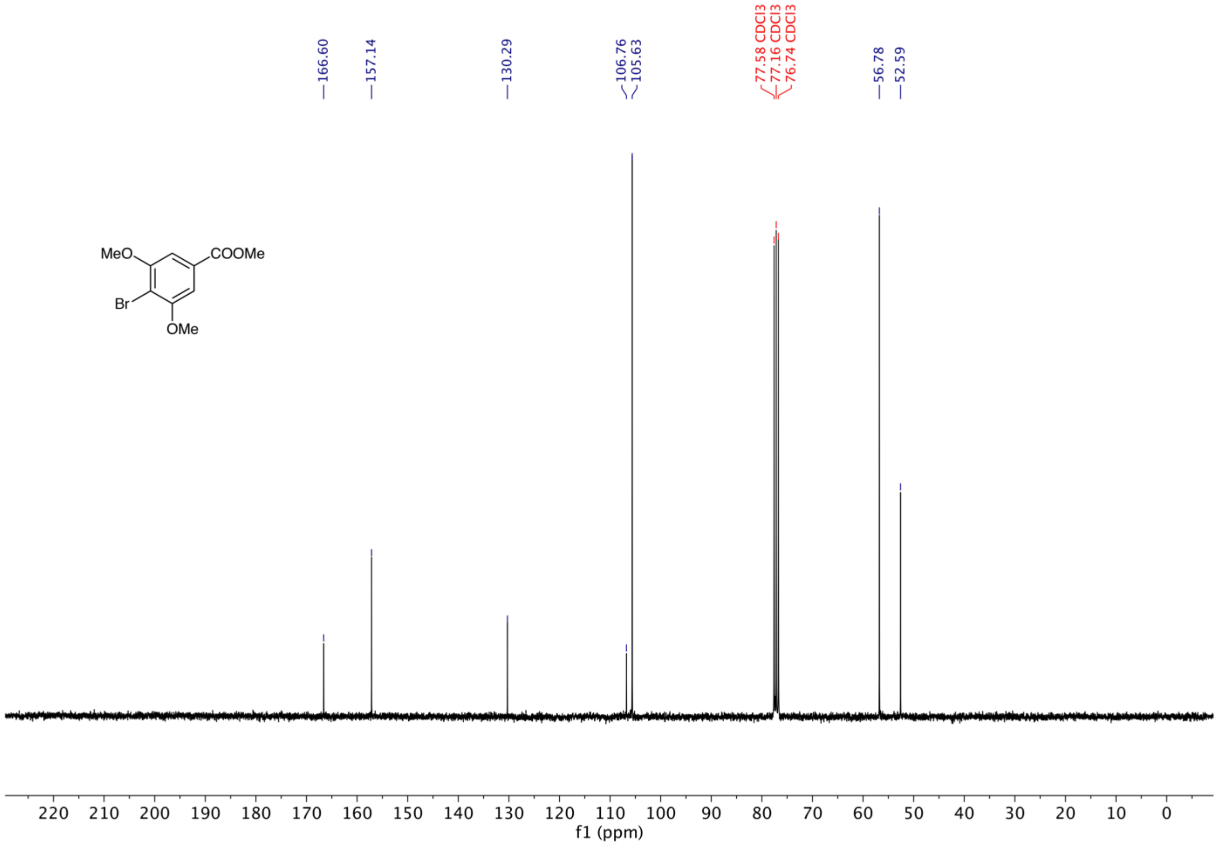

^{13}C-NMR (75.5 MHz, , $CDCl_3$): Methyl 4-bromo-3,5-dimethoxybenzoate (**31**).

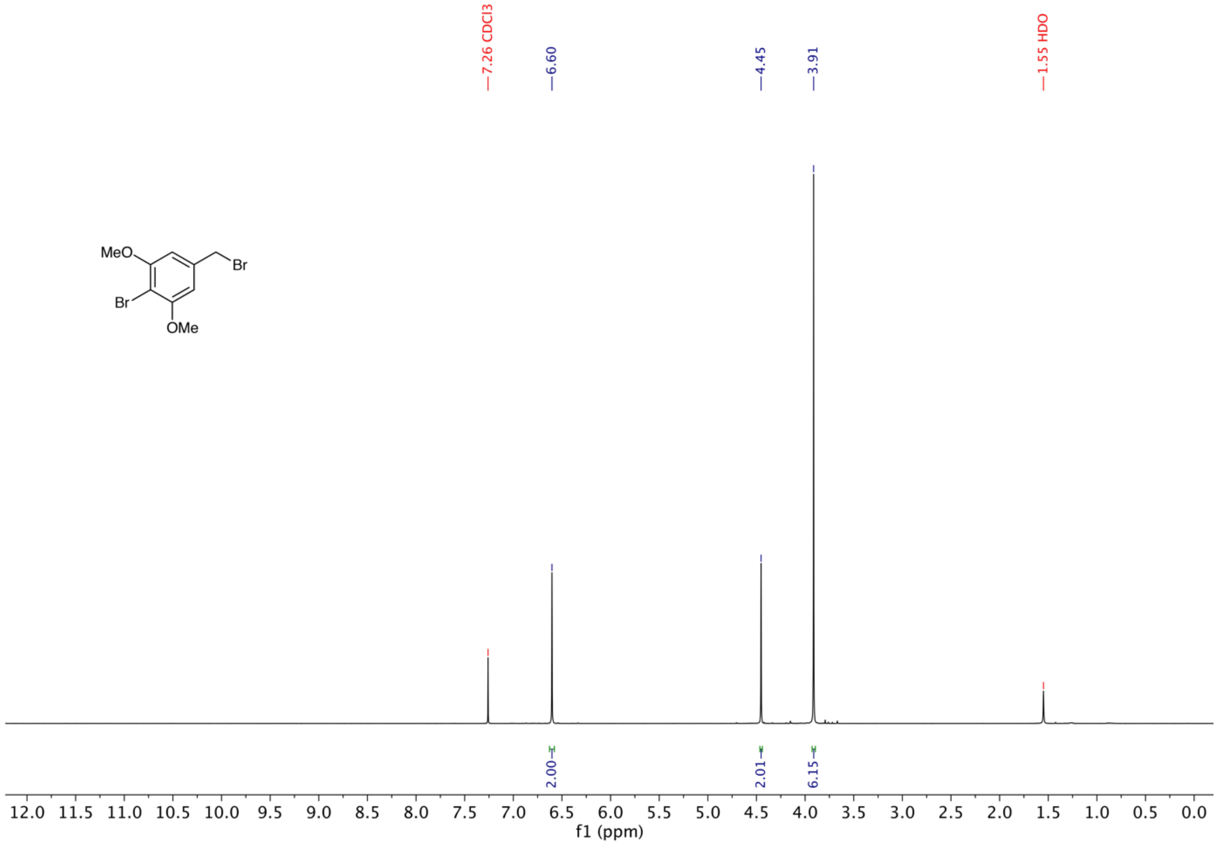

^{1}H-NMR (300.1 MHz, $CDCl_3$): 2-Bromo-5-(bromomethyl)-1,3-dimethoxybenzene (**33**).

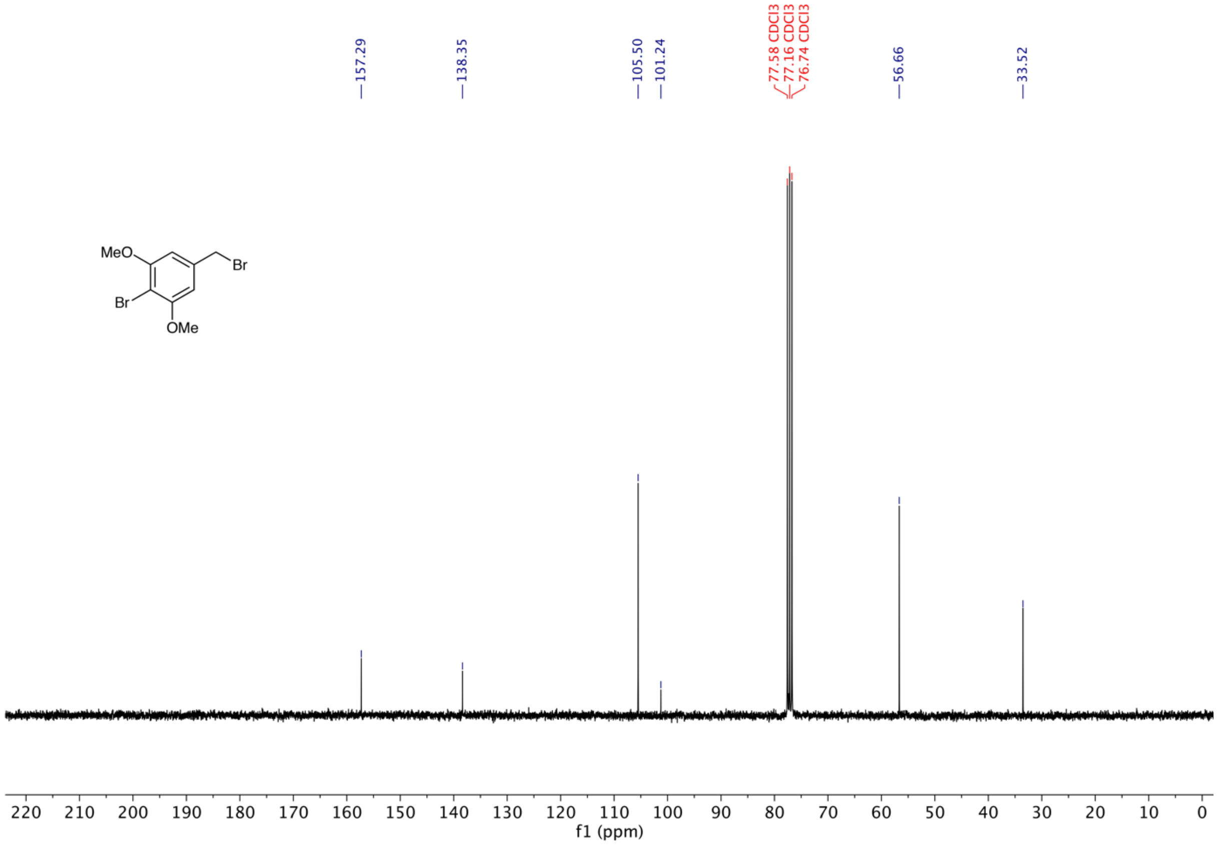

^{13}C-NMR (75.5 MHz, , $CDCl_3$): 2-Bromo-5-(bromomethyl)-1,3-dimethoxybenzene (**33**).

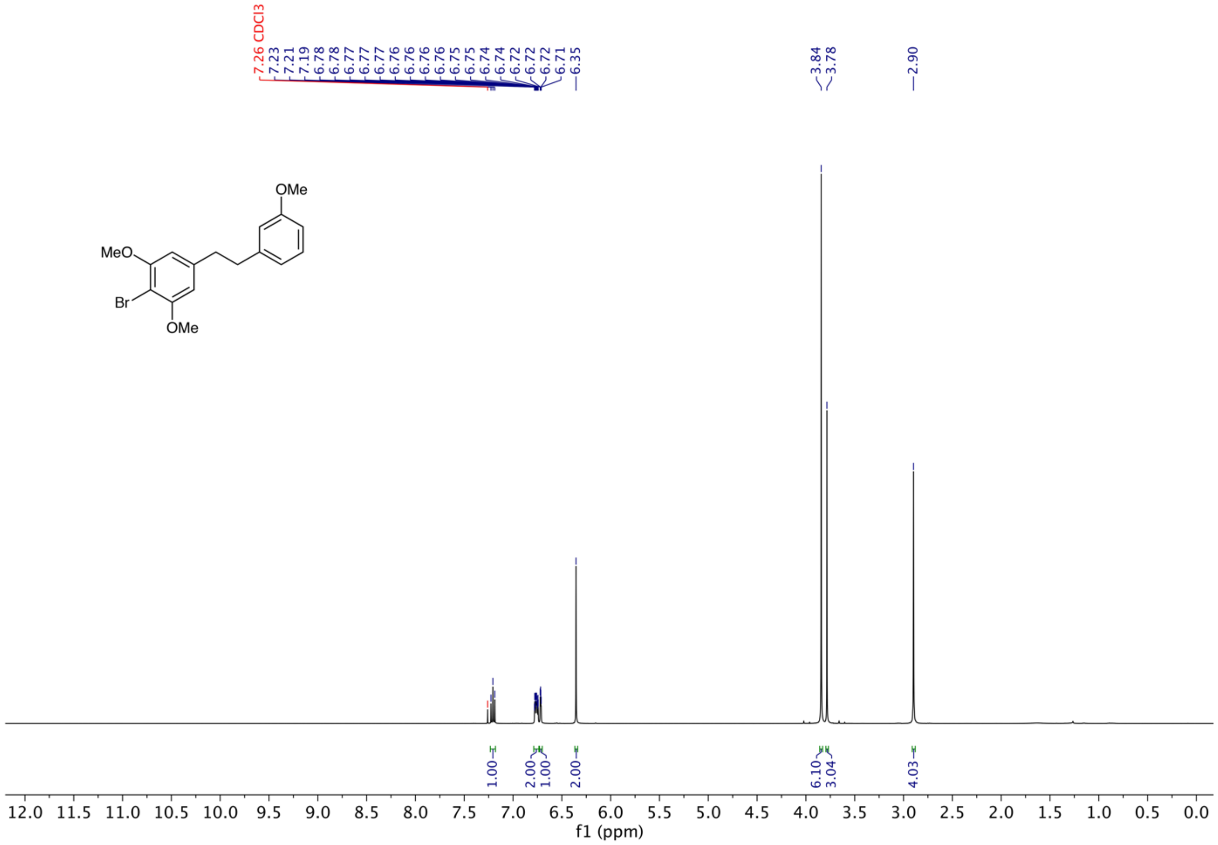

^{1}H-NMR (400.1 MHz, $CDCl_3$): 2-Bromo-1,3-dimethoxy-5-(2-(3-methoxyphenyl)ethyl)benzene (**27**).

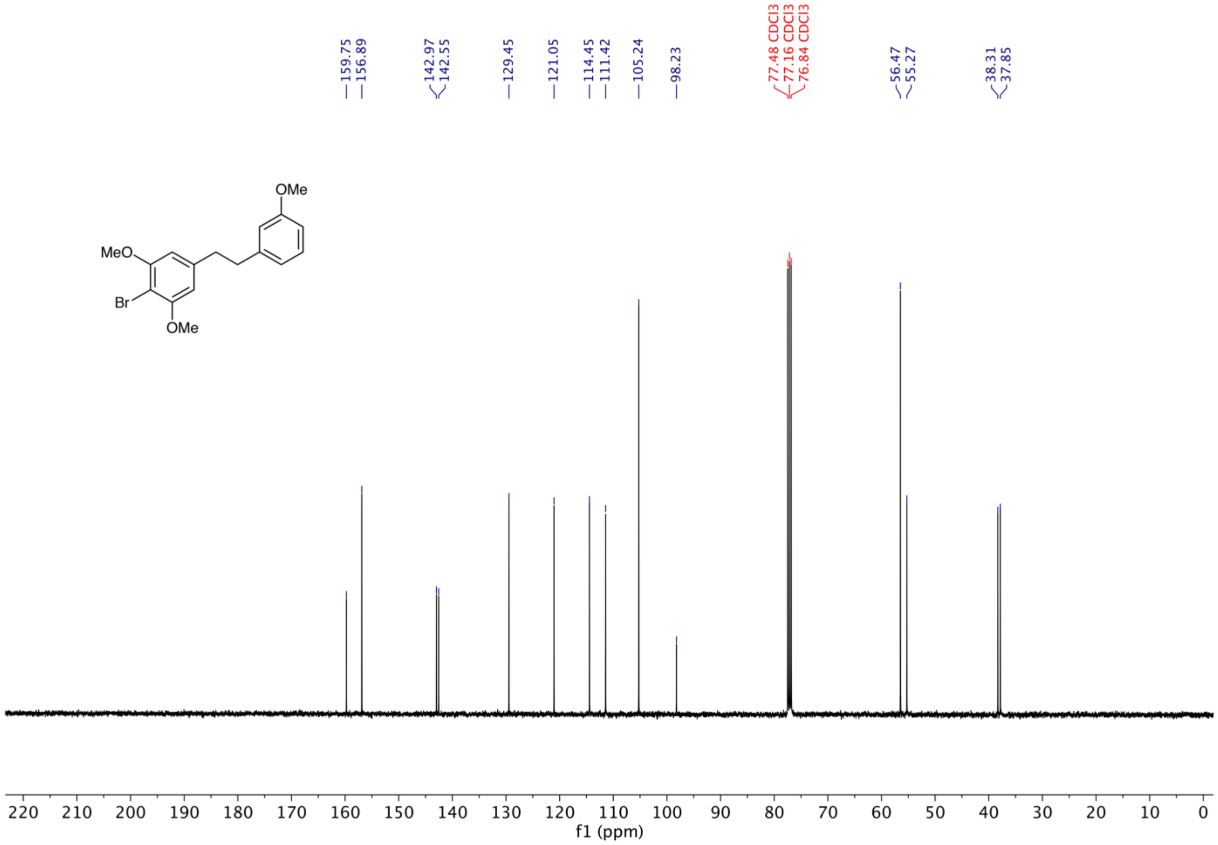

^{13}C-NMR (100.6 MHz, $CDCl_3$): 2-Bromo-1,3-dimethoxy-5-(2-(3-methoxyphenyl)ethyl)benzene (**27**).

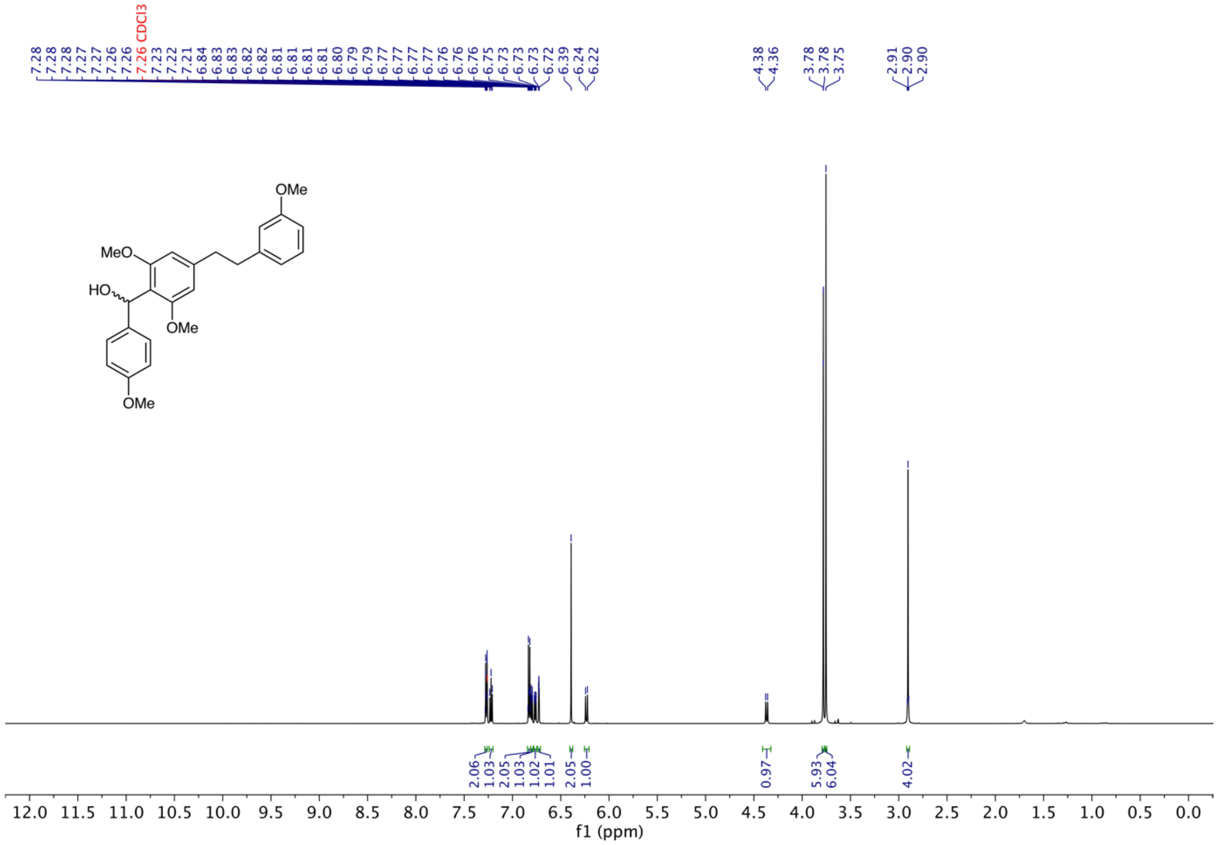

^{1}H-NMR (600.1 MHz, $CDCl_3$): (2,6-Dimethoxy-4-(2-(3-methoxyphenyl)ethyl)phenyl)(4-methoxyphenyl)methanol (**36**).

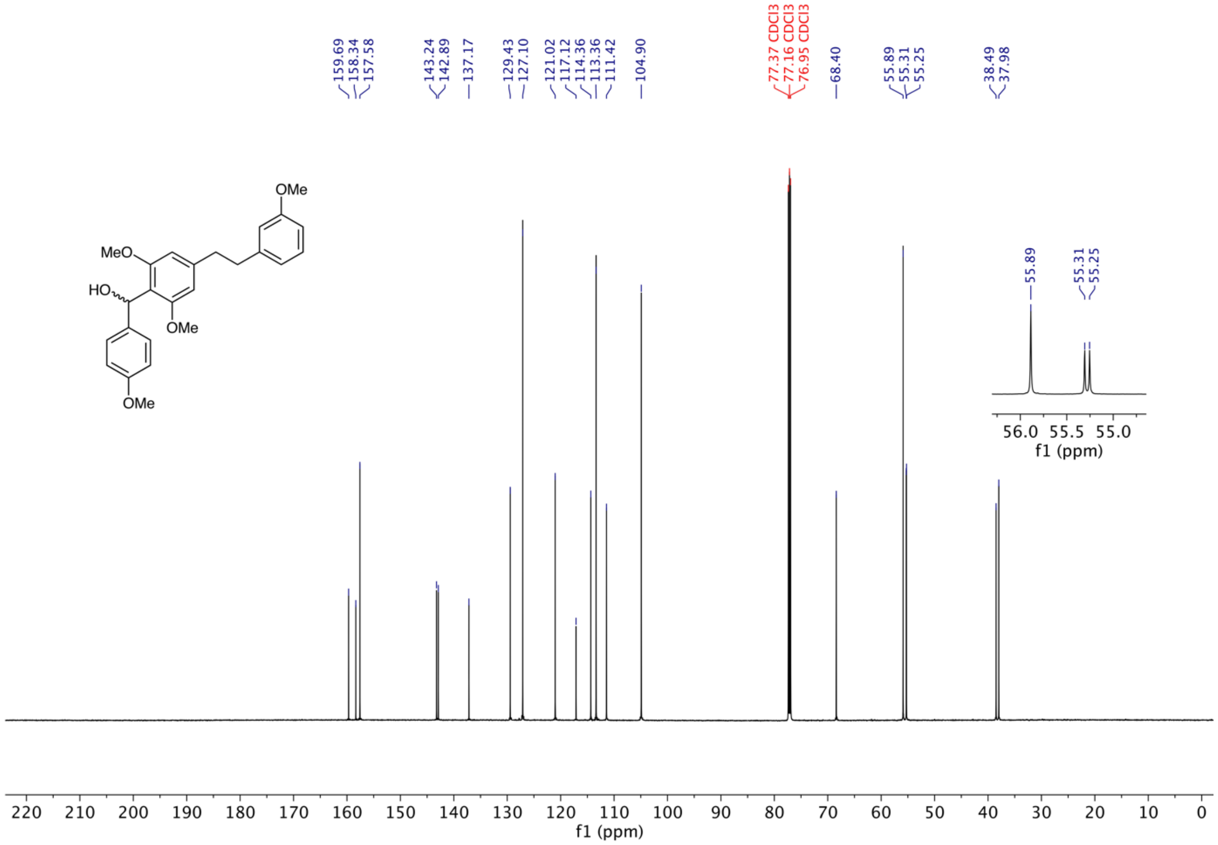

^{13}C-NMR (150.9 MHz, $CDCl_3$): (2,6-Dimethoxy-4-(2-(3-methoxyphenyl)ethyl)phenyl)(4-methoxyphenyl)methanol (**36**).

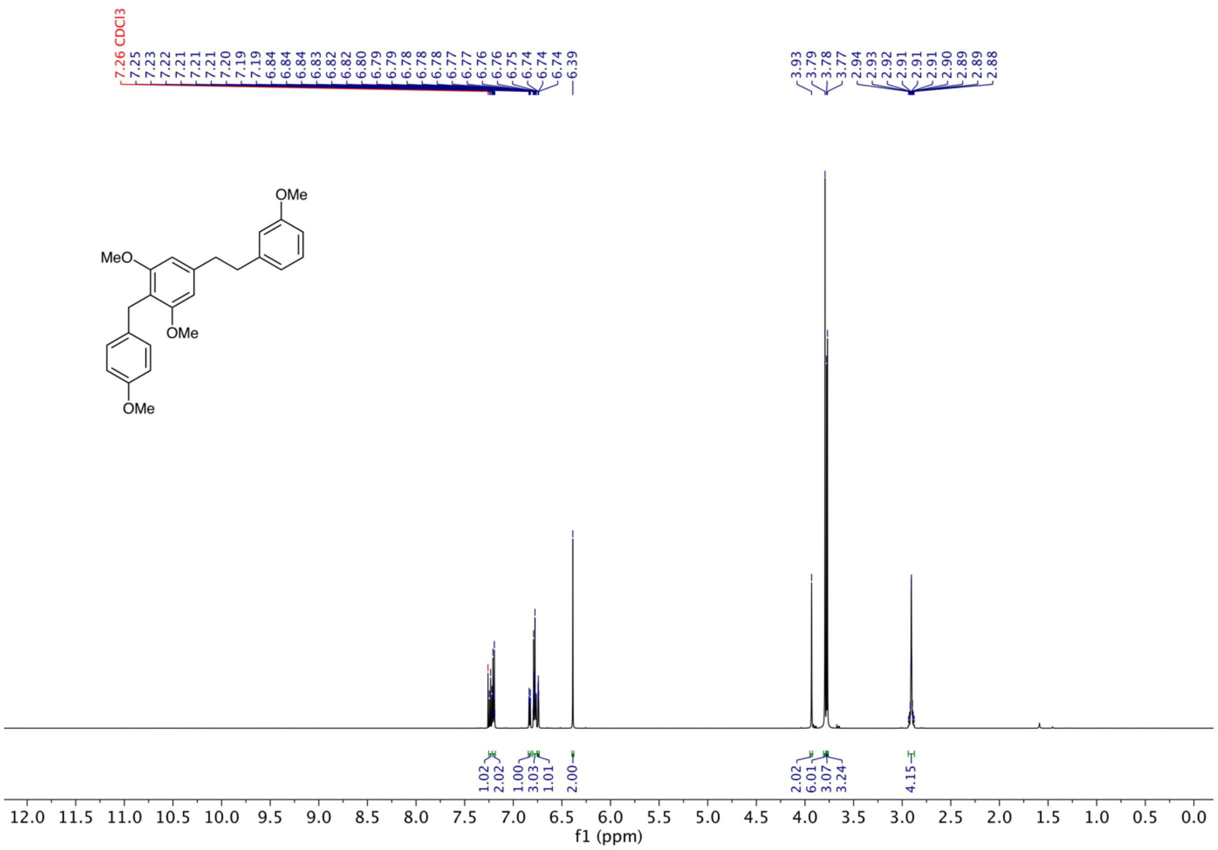

^{1}H-NMR (600.1 MHz, $CDCl_3$): 1,3-Dimethoxy-2-(4-methoxybenzyl)-5-(2-(3-methoxyphenyl)ethyl)benzene (**26**).

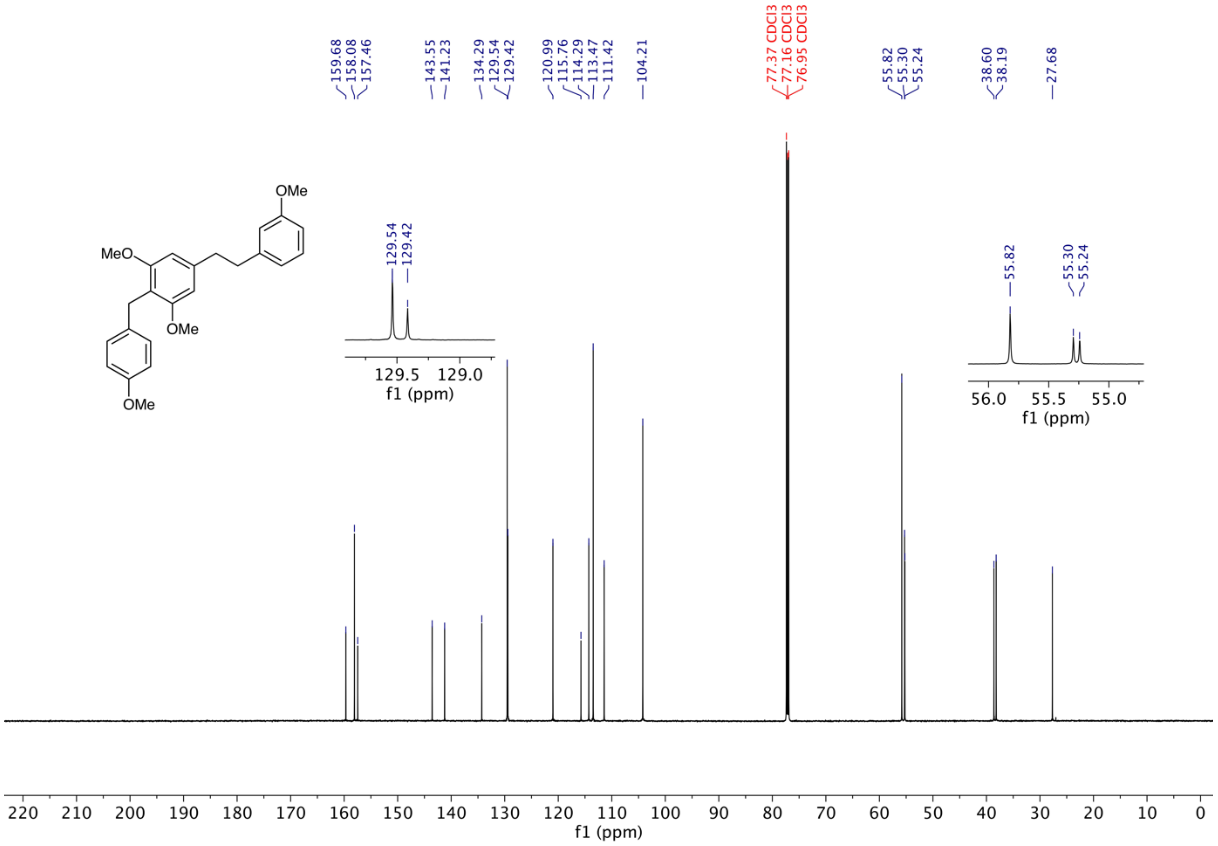

^{13}C-NMR (150.9 MHz, $CDCl_3$): 1,3-Dimethoxy-2-(4-methoxybenzyl)-5-(2-(3-methoxyphenyl)ethyl)benzene (**26**).

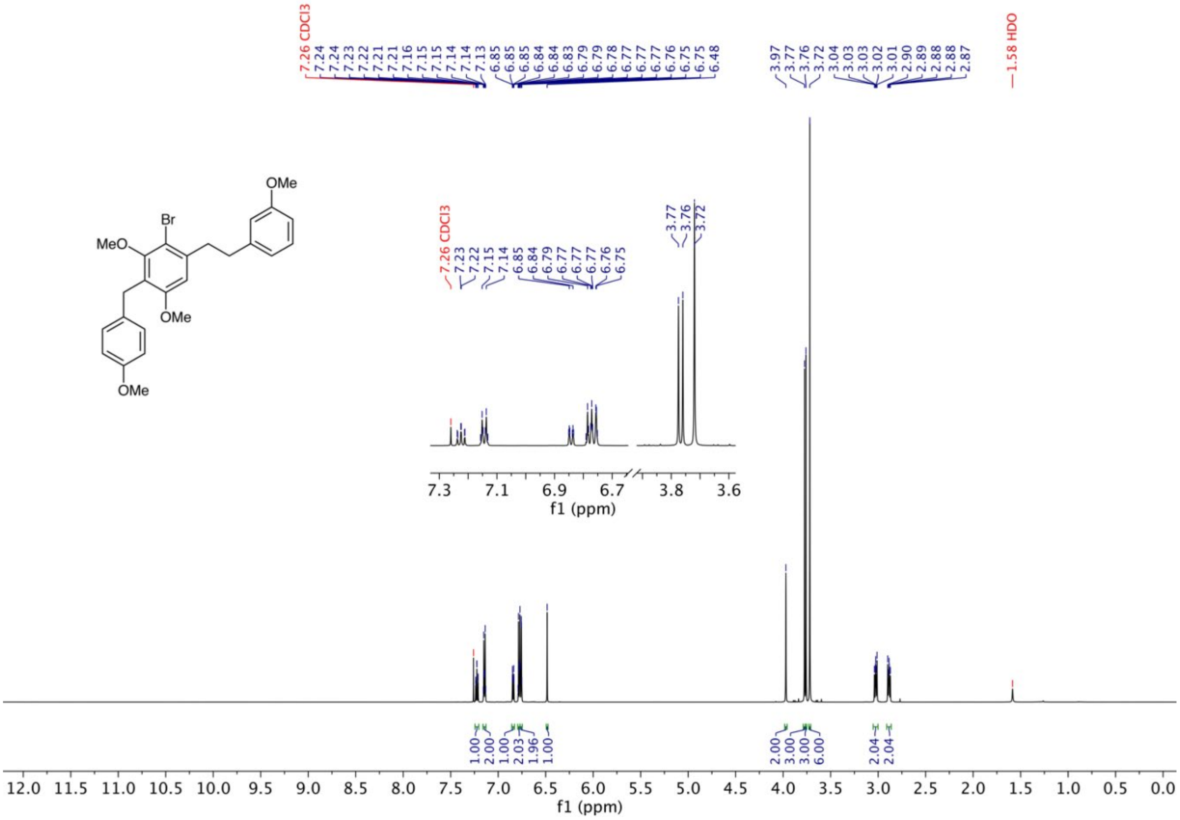

^{1}H-NMR (600.1 MHz, $CDCl_3$): 2-Bromo-3,5-dimethoxy-4-(4-methoxybenzyl)-1-(2-(3-methoxyphenyl)ethyl)benzene (**37**).

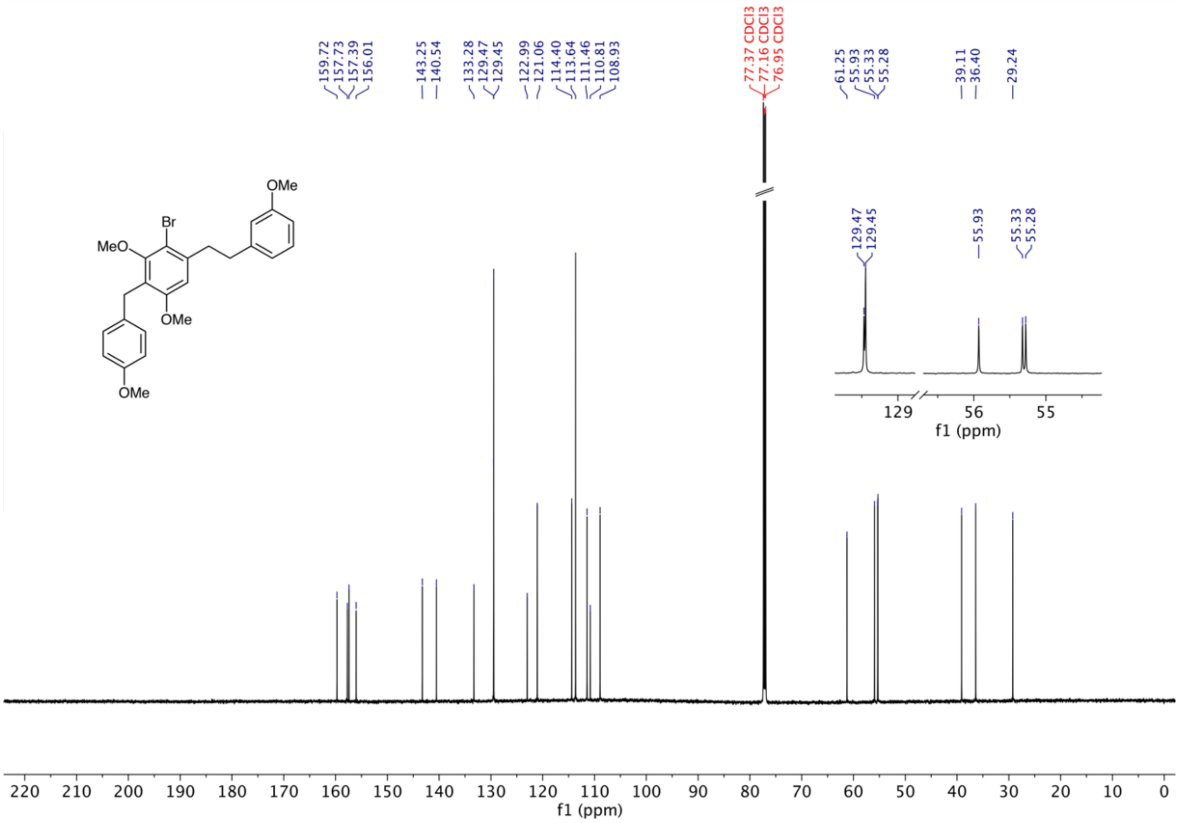

^{13}C-NMR (150.9 MHz, $CDCl_3$): 2-Bromo-3,5-dimethoxy-4-(4-methoxybenzyl)-1-(2-(3-methoxyphenyl)ethyl)benzene (**37**).

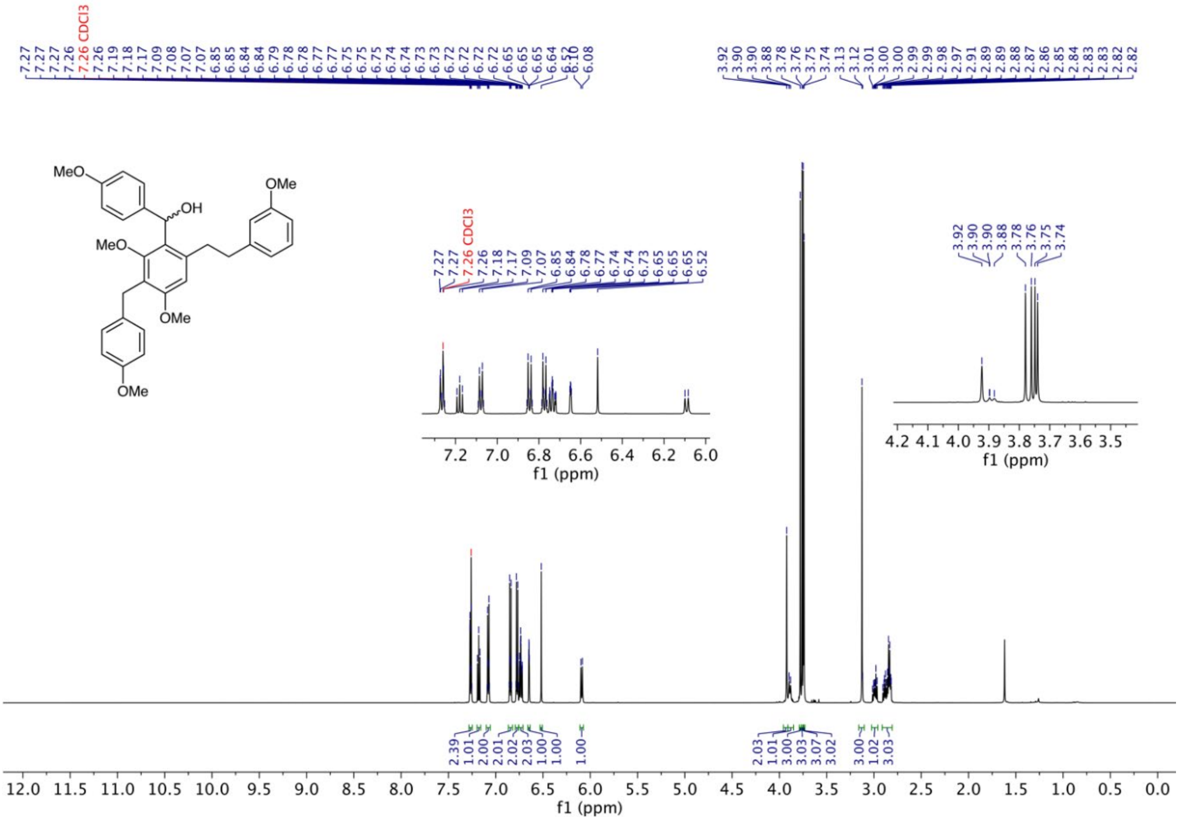

^{1}H-NMR (600.1 MHz, $CDCl_3$): (2,4-Dimethoxy-3-(4-methoxybenzyl)-6-(2-(3-methoxyphenyl)ethyl)phenyl)(4-methoxyphenyl)methanol (**38**).

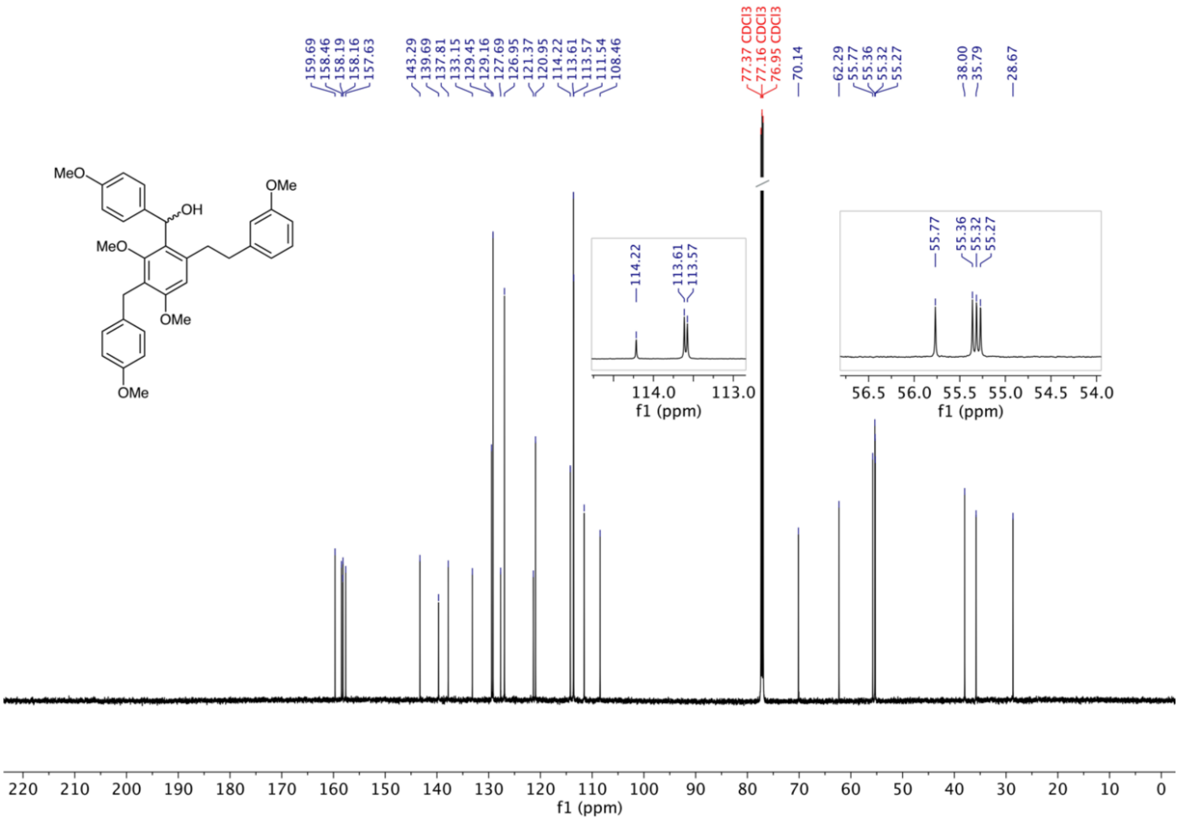

^{13}C-NMR (150.9 MHz, $CDCl_3$): (2,4-Dimethoxy-3-(4-methoxybenzyl)-6-(2-(3-methoxyphenyl)ethyl)phenyl)(4-methoxyphenyl)methanol (**38**).

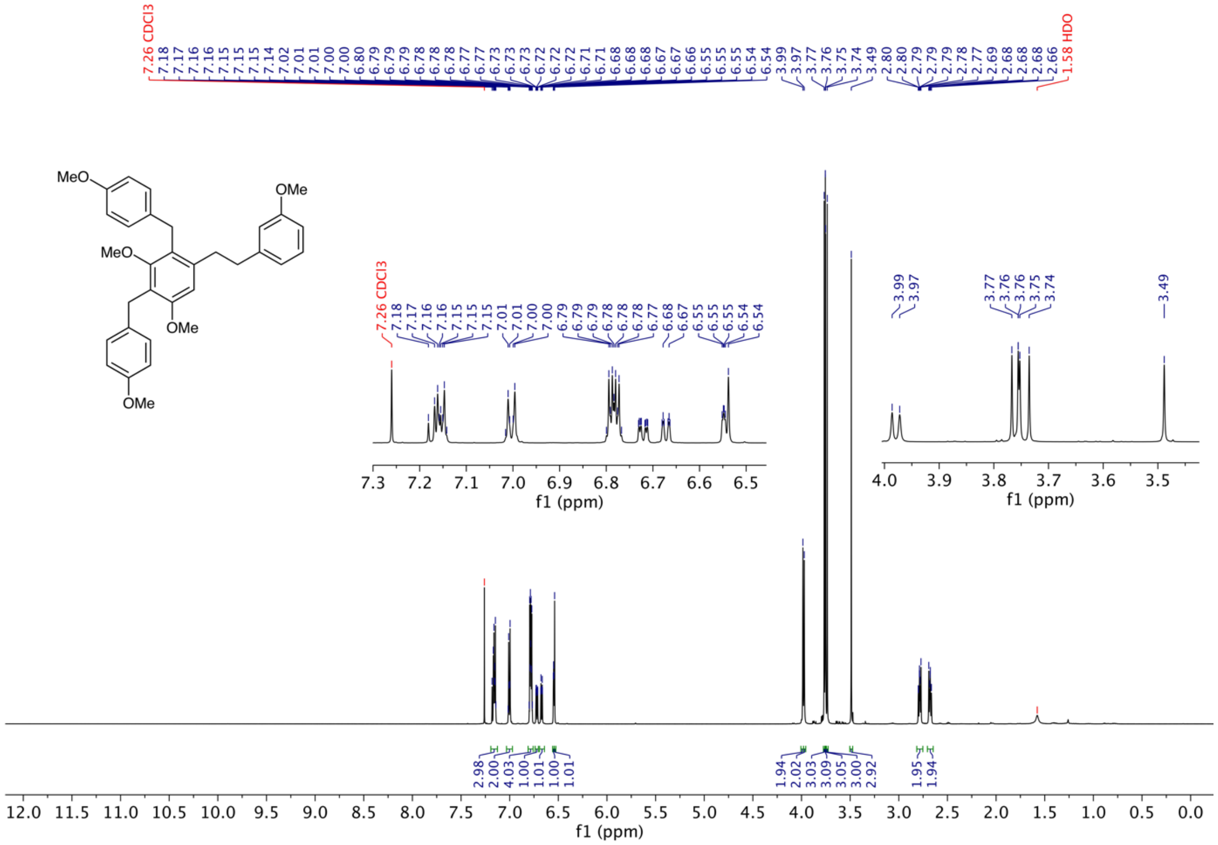

^{1}H-NMR (600.1 MHz, $CDCl_3$): 1,3-Dimethoxy-2,4-bis(4-methoxybenzyl)-5-(2-(3-methoxyphenyl)ethyl)benzene (**41**).

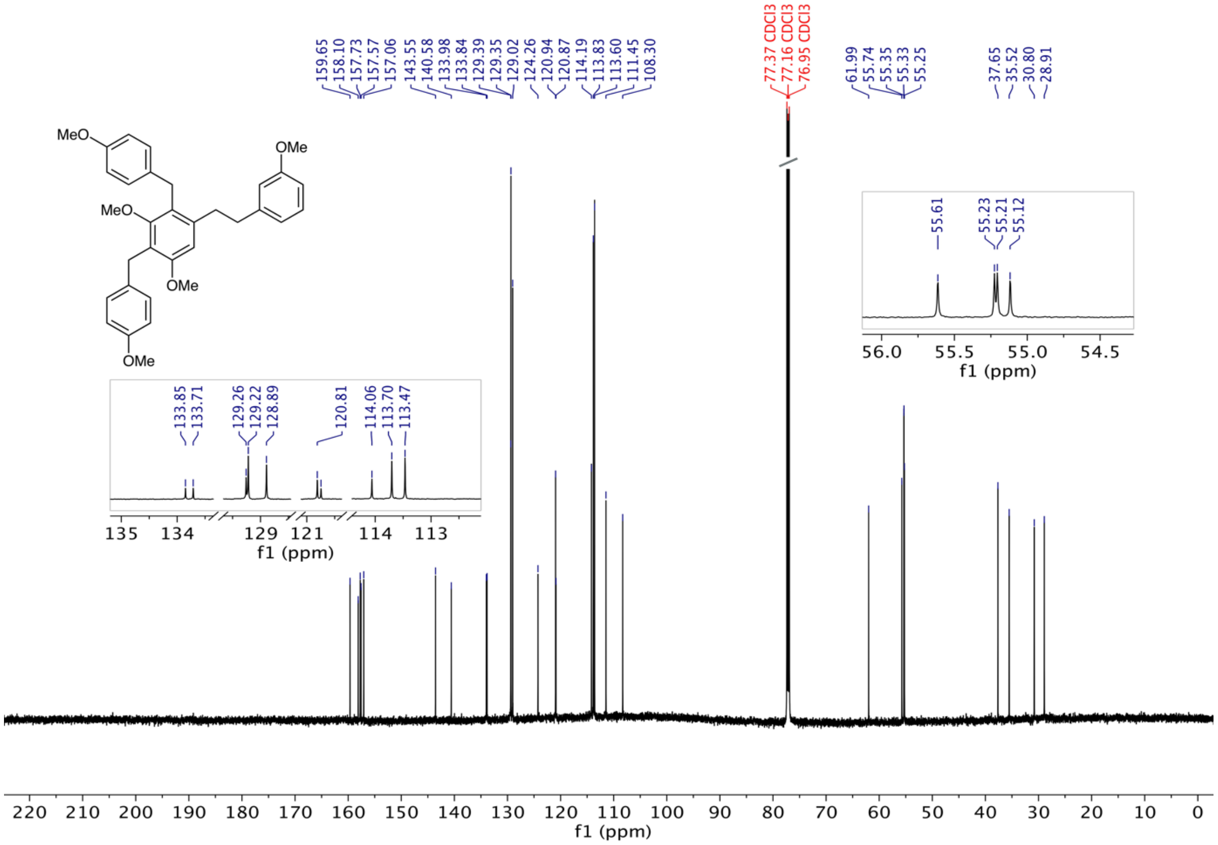

^{13}C-NMR (150.9 MHz, $CDCl_3$): 1,3-Dimethoxy-2,4-bis(4-methoxybenzyl)-5-(2-(3-methoxyphenyl)ethyl)benzene (**41**).

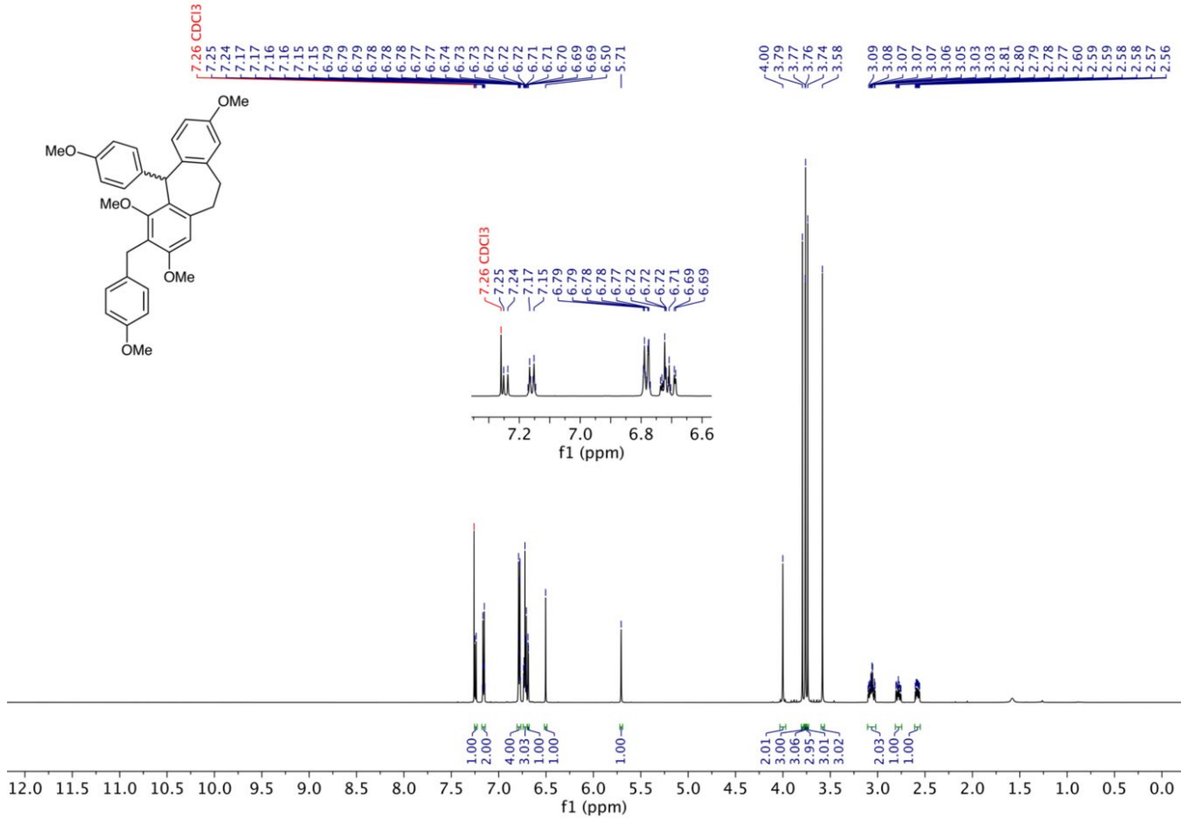

^{1}H-NMR (600.1 MHz, $CDCl_3$): 2,4,8-Trimethoxy-3-(4-methoxybenzyl)-5-(4-methoxyphenyl)-10,11-dihydro-5*H*-dibenzo[*a*,*d*][7]-annulene (**40**).

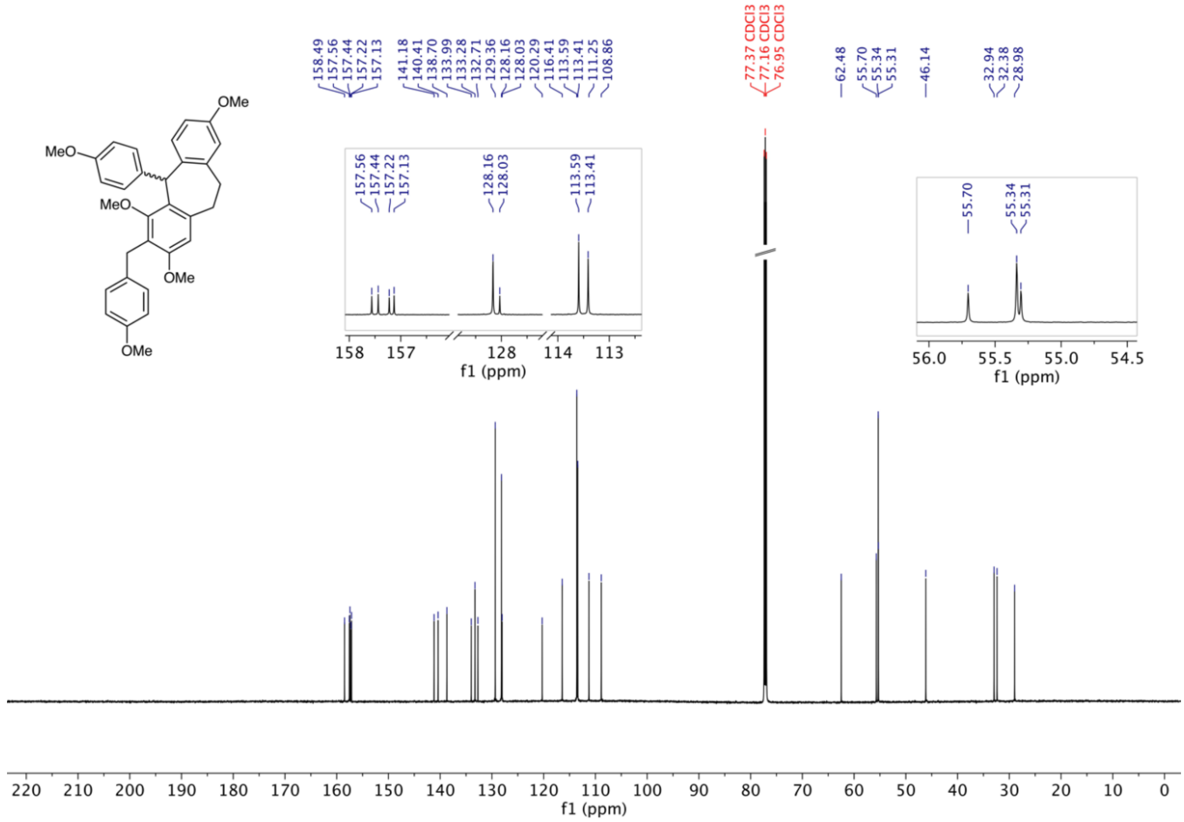

^{13}C-NMR (150.9 MHz, $CDCl_3$): 2,4,8-Trimethoxy-3-(4-methoxybenzyl)-5-(4-methoxyphenyl)-10,11-dihydro-5*H*-dibenzo[*a*,*d*][7]-annulene (**40**).

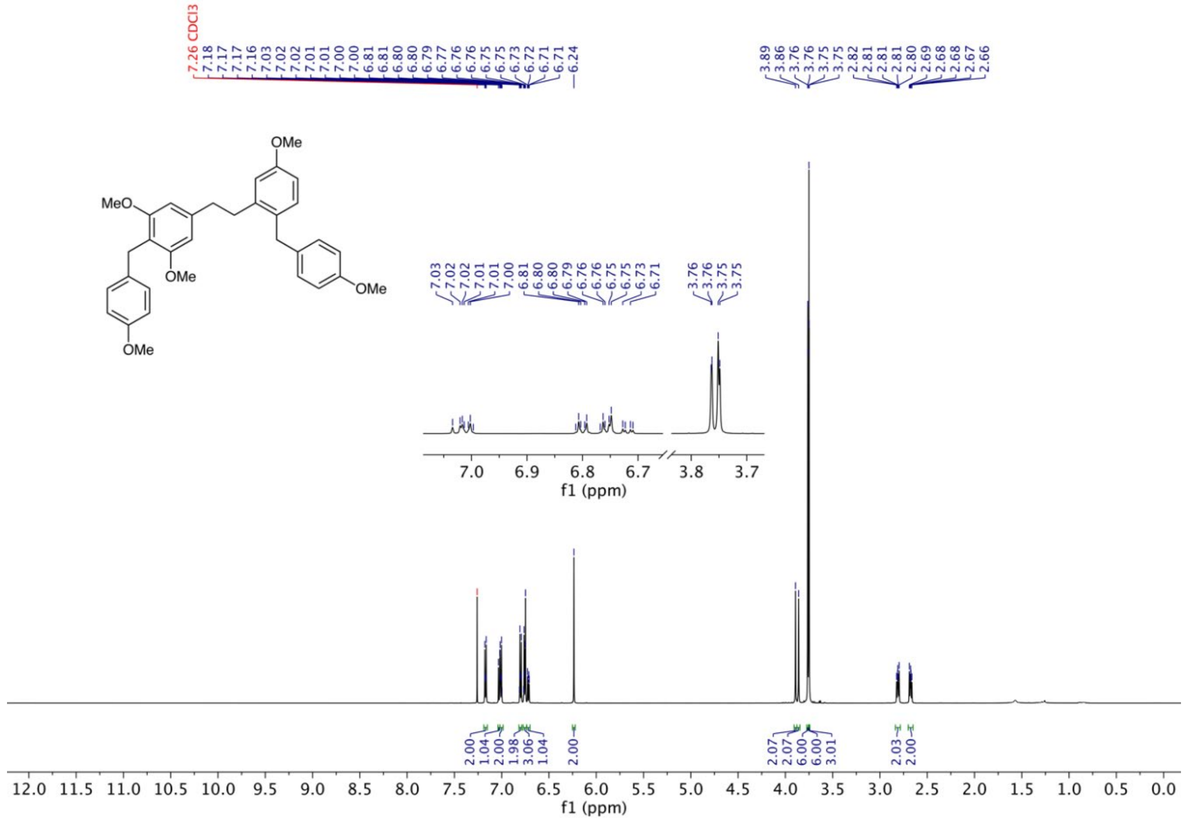

^{1}H-NMR (600.1 MHz, $CDCl_3$): 1,3-Dimethoxy-5-(2-(5-methoxy-2-(4-methoxybenzyl)phenyl)ethyl)-2-(4-methoxybenzyl)benzene (**39**).

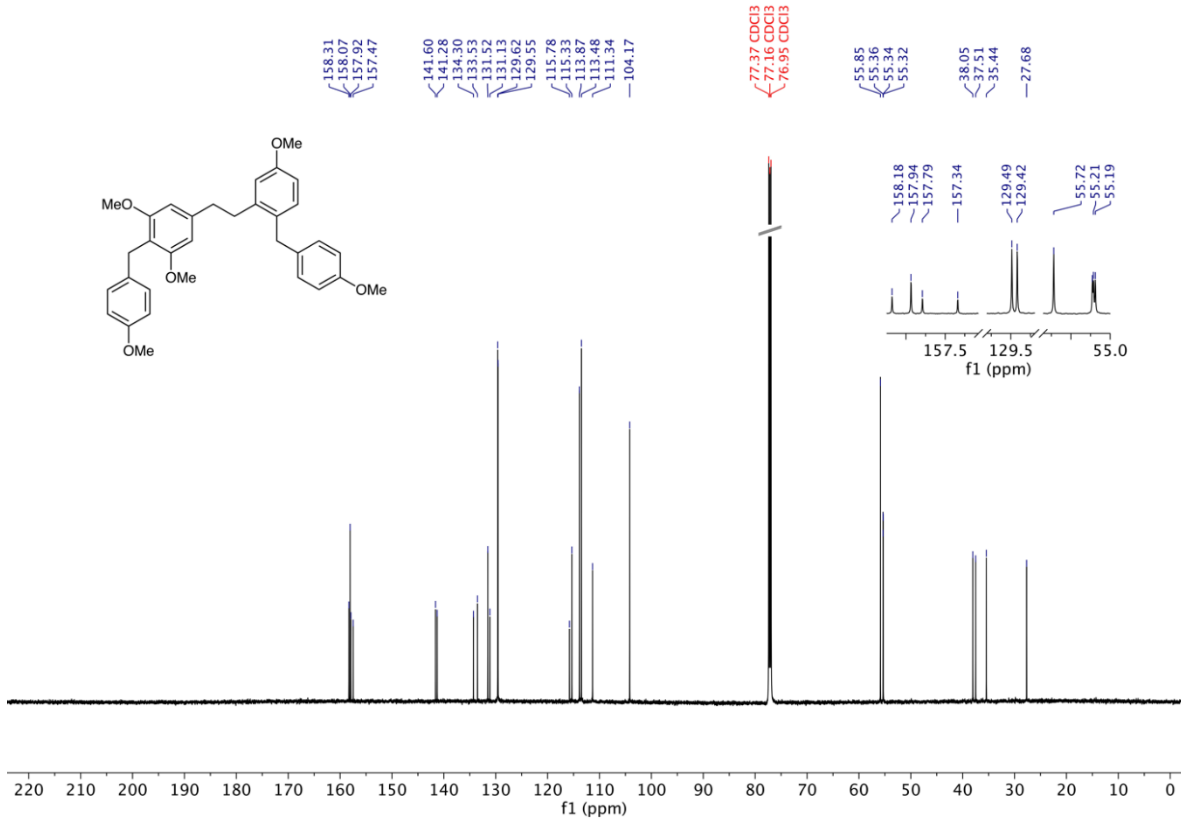

^{13}C-NMR (150.9 MHz, $CDCl_3$): 1,3-Dimethoxy-5-(2-(5-methoxy-2-(4-methoxybenzyl)phenyl)ethyl)-2-(4-methoxybenzyl)benzene (**39**).

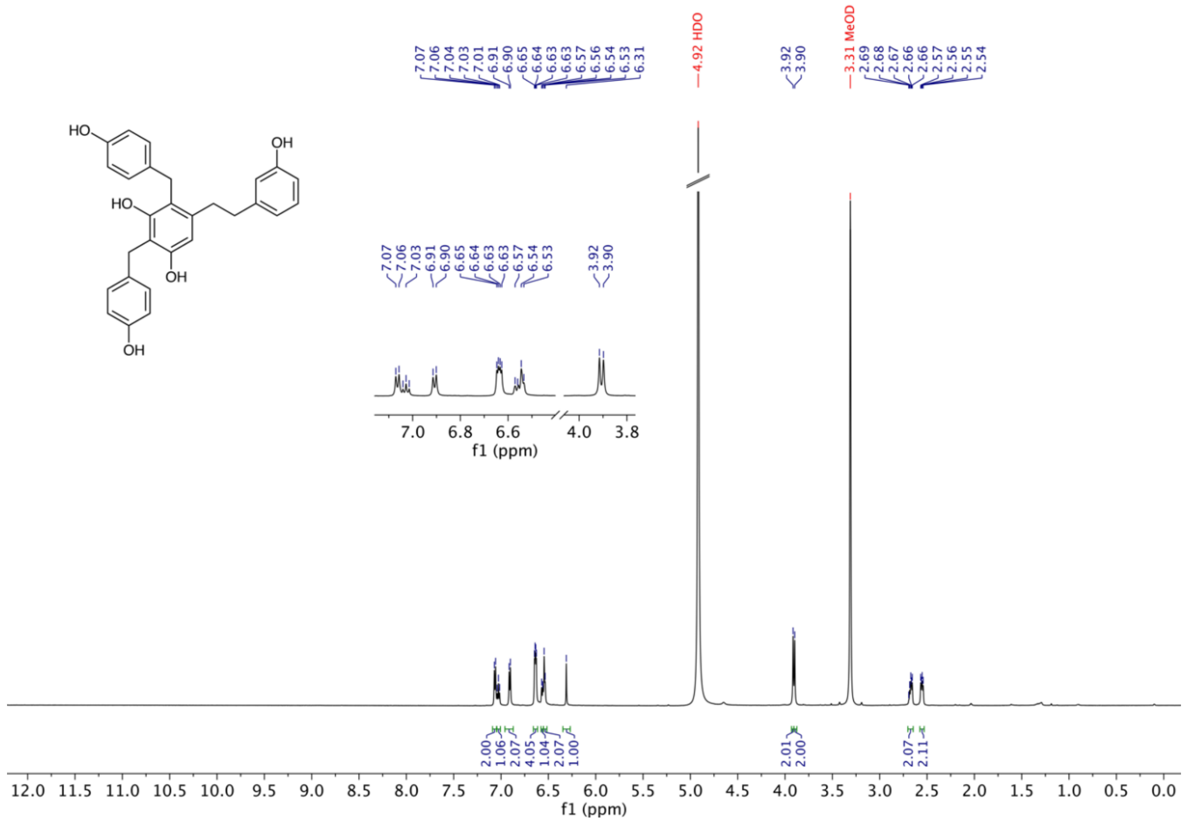

^{1}H-NMR (600.1 MHz, MeOD): 2,4-Bis(4-hydroxybenzyl)-5-(2-(3-hydroxyphenyl)ethyl)benzene-1,3-diol (bletistrin G, **2**).

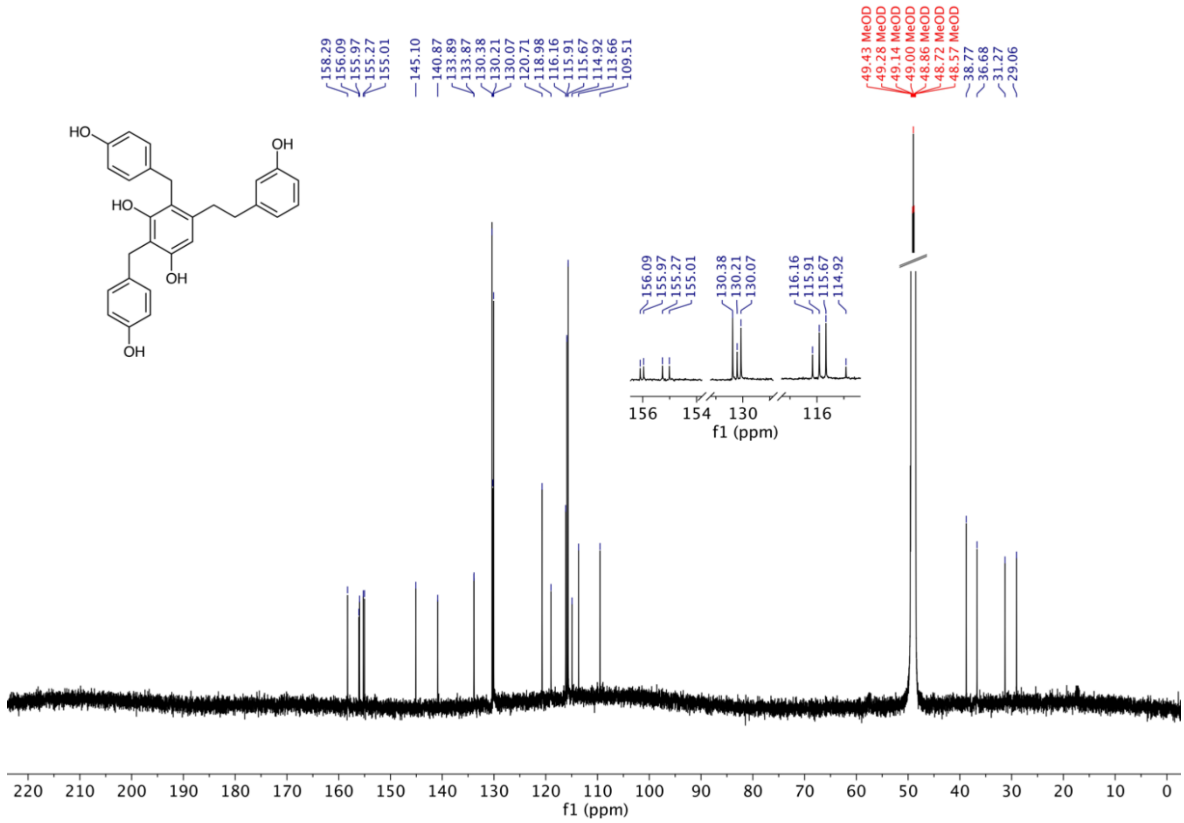

^{13}C-NMR (150.9 MHz, MeOD): 2,4-Bis(4-hydroxybenzyl)-5-(2-(3-hydroxyphenyl)ethyl)benzene-1,3-diol (bletistrin G, **2**).

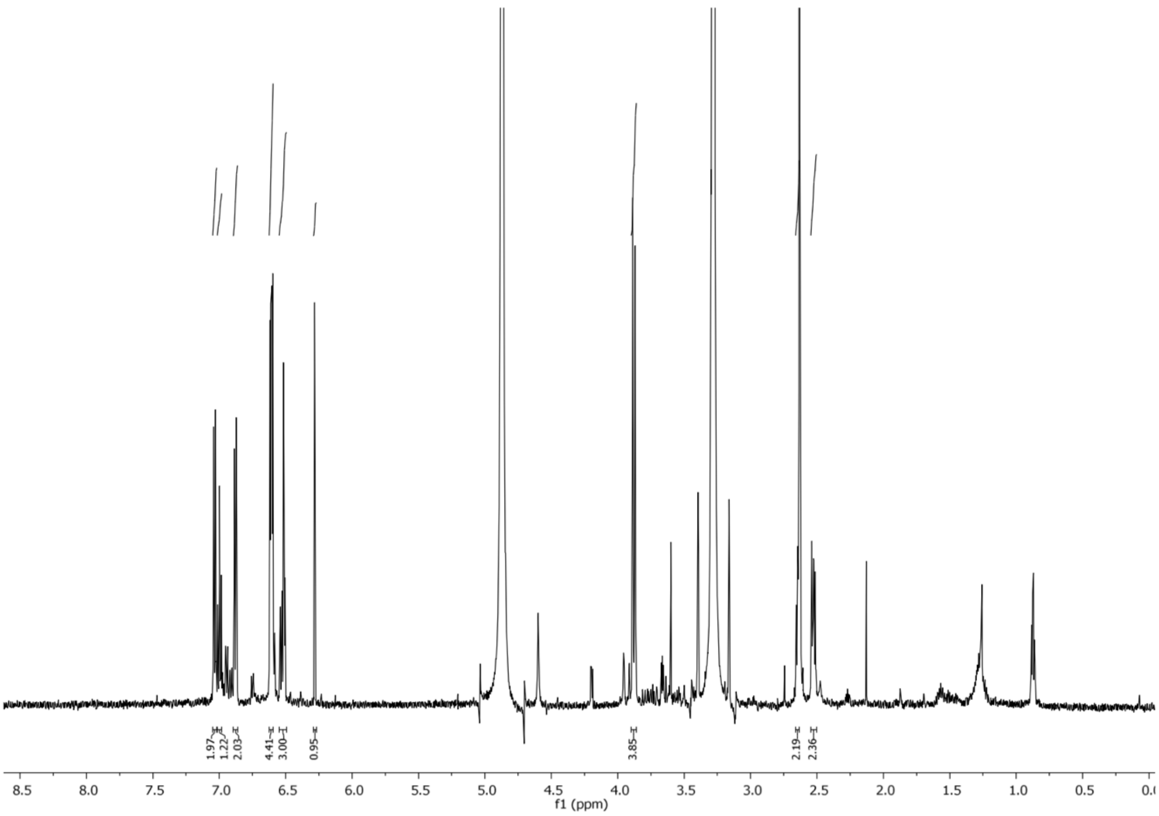

^{1}H-NMR (600.1 MHz, MeOD) of natural bletistrin G, **2**) [20].

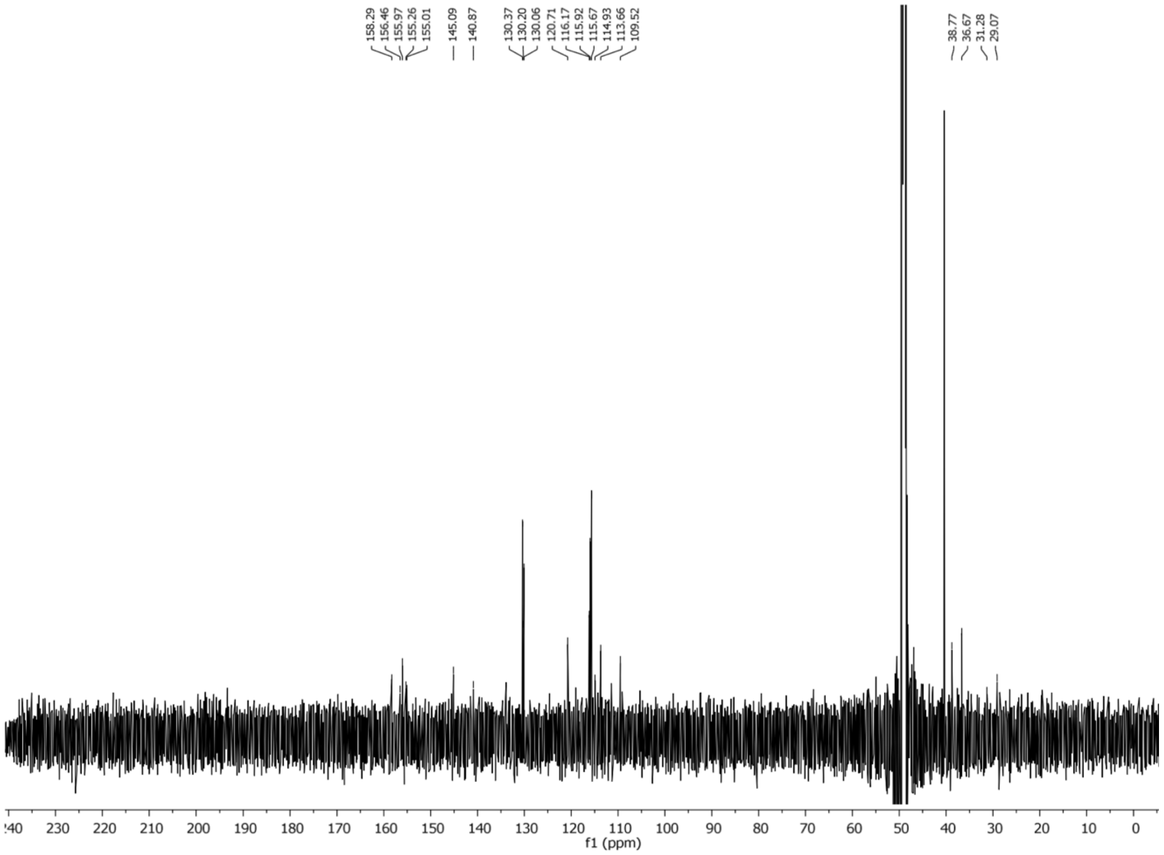

^{13}C-NMR (150.9 MHz, MeOD) of natural bletistrin G, **2**) [20].

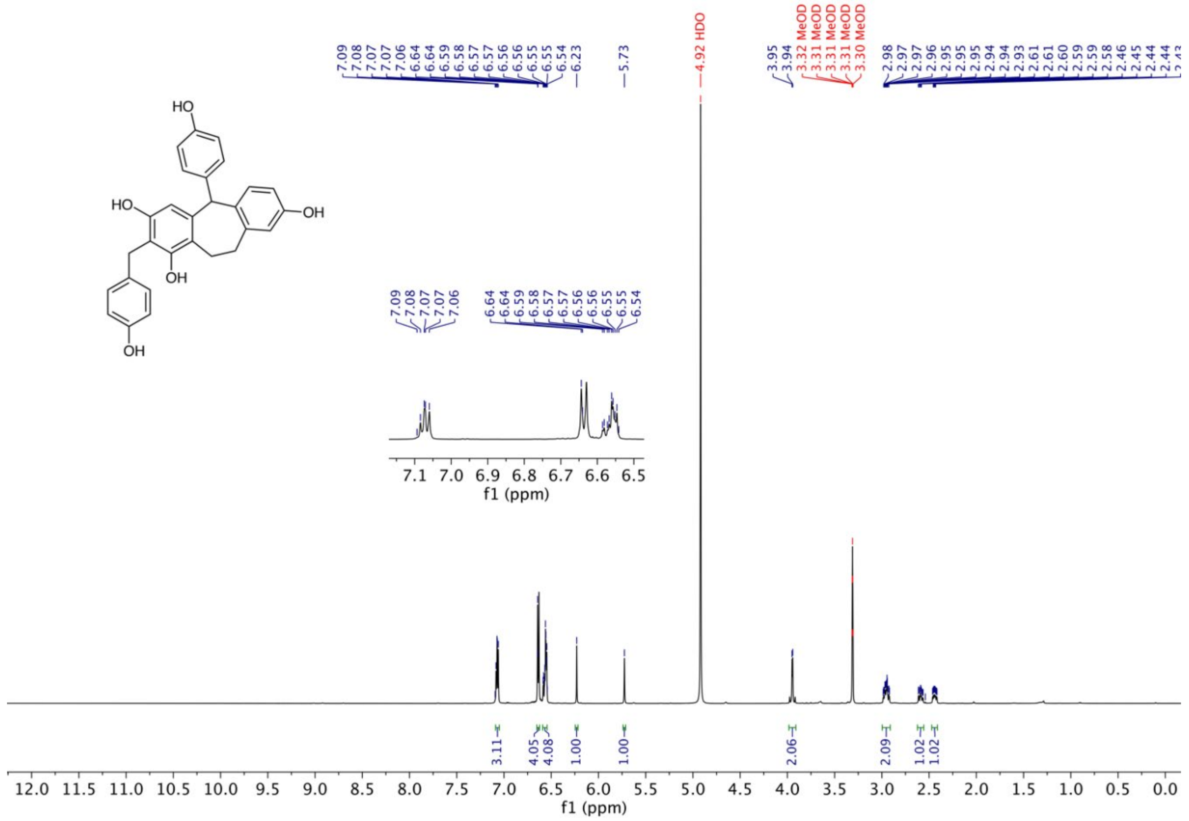

^{1}H-NMR (600.1 MHz, MeOD): 3-(4-Hydroxybenzyl)-5-(4-hydroxyphenyl)-10,11-dihydro-5*H*-dibenzo[*a*,*d*][7]annulene-2,4,8-triol (**42**).

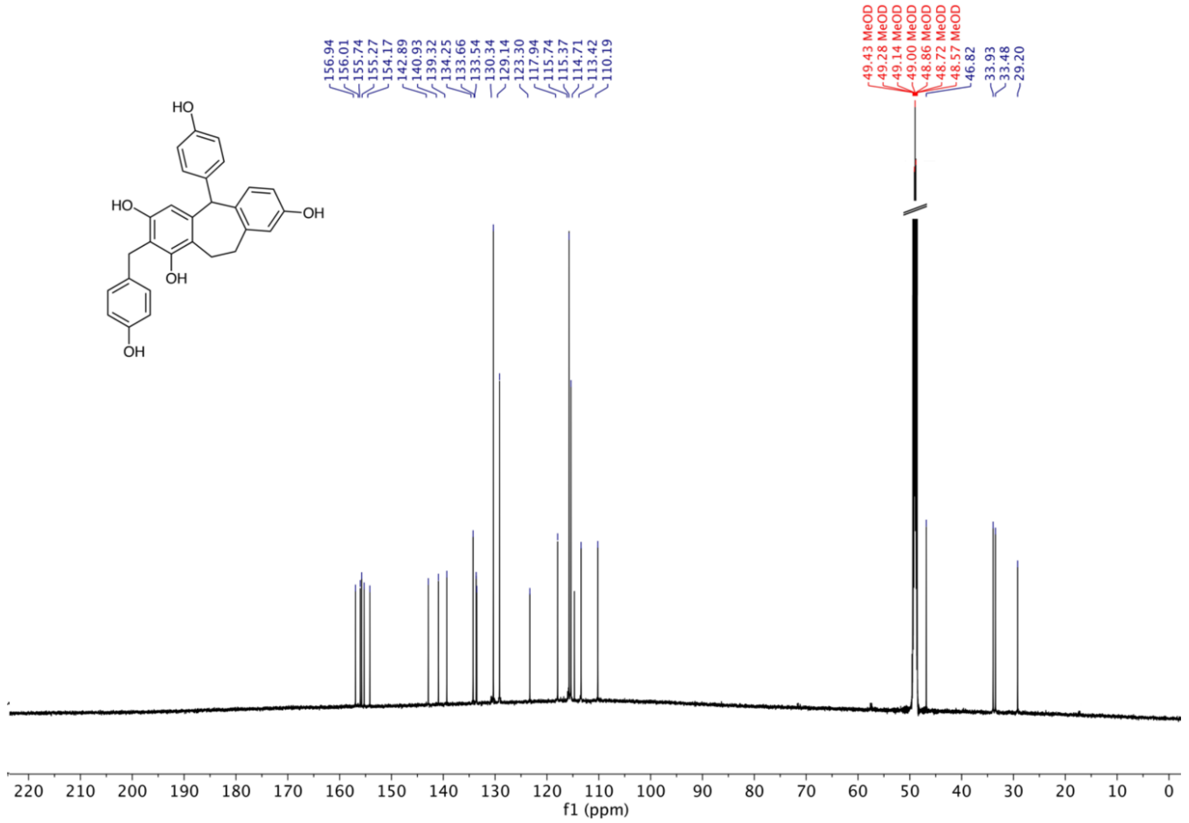

^{13}C-NMR (150.9 MHz, MeOD): 3-(4-Hydroxybenzyl)-5-(4-hydroxyphenyl)-10,11-dihydro-5*H*-dibenzo[*a*,*d*][7]annulene-2,4,8-triol (**42**).

4. References

1. Lim, Z.; Duggan, P.J.; Meyer, A.G.; Tuck, K.L. An Iterative in Silico and Modular Synthetic Approach to Aqueous Soluble Tercyclic α-Helix Mimetics. *Org. Biomol. Chem.* **2014**, *12*, 4432–4444.
2. Node, M.; Kodama, S.; Hamashima, Y.; Katoh, T.; Nishide, K.; Kajimoto, T. Biomimetic Synthesis of (±)-Galanthamine and Asymmetric Synthesis of (±)-Galanthamine Using Remote Asymmetric Induction. *Chem. Pharm. Bull.* **2006**, *54*, 1662–1679.
3. Ogura, T.; Usuki, T. Total Synthesis of Acerogenins E, G and K, and Centrolobol. *Tetrahedron* **2013**, *69*, 2807–2815.
4. Dallinger, D.; Pinho, V.D.; Gutmann, B.; Kappe, C.O. Laboratory-Scale Membrane Reactor for the Generation of Anhydrous Diazomethane. *J. Org. Chem.* **2016**, *81*, 5814–5823.
5. Liang, Q.; Zhang, J.; Quan, W.; Sun, Y.; She, X.; Pan, X. The First Asymmetric Total Syntheses and Determination of Absolute Configurations of Xestodecalactones B and C. *J. Org. Chem.* **2007**, *72*, 2694–2697.
6. Peixoto, D.; Figueiredo, M.; Gawande, M.B.; Corvo, M.C.; Vanhoenacker, G.; Afonso, C.A.M.; Ferreira, L.M.; Branco, P.S. Developments in the Reactivity of 2-Methylimidazolium Salts. *J. Org. Chem.* **2017**, *82*, 6232–6241.
7. Keenan, M.; Abbott, M.J.; Alexander, P.W.; Armstrong, T.; Best, W.M.; Berven, B.; Botero, A.; Chaplin, J.H.; Charman, S.A.; Chatelain, E.; von Geldern, T.W.; Kerfoot, M.; Khong, A.; Nguyen, T.; McManus, J.D.; Morizzi, J.; Ryan, E.; Scandale, I.; Thompson, R.A.; Wang, S.Z.; White, K.L. Analogues of Fenarimol Are Potent Inhibitors of *Trypanosoma cruzi* and Are Efficacious in a Murine Model of Chagas Disease. *J. Med. Chem.* **2012**, *55*, 4189–4204.
8. Quesnel, J.S.; Arndtsen, B.A. A Palladium-Catalyzed Carbonylation Approach to Acid Chloride Synthesis. *J. Am. Chem. Soc.* **2013**, *135*, 16841–16844.
9. Adams, R.; Chiles, H.M.; Rassweiler, C.F. Acetonedicarboxylic Acid, *Org. Synth.* **1925**, *5*, 5.
10. Van Tamelen, E.E.; Placeway, C.; Schiemenz, G.P.; Wright, I.G. Total Syntheses of dl-Ajmalicine and Emetine. *J. Am. Chem. Soc.* **1969**, *91*, 7359–7371.
11. Winkel, C.; Buitenhuis, E.G.; Lugtenburg, J. Synthesis and Spectroscopic Study of 13C-Labelled Citric Acids. *Recl. Trav. Chim. Pays-Bas* **1989**, *108*, 51–56.
12. Theilacker, W.; Schmid, W. Zur Konstitution der Triacylmethane. II. Über das Bicyclo-[2,2,2]-octantrion-(2,6,7). *Liebigs Ann.* **1950**, *570*, 15–33.
13. Elzner, S.; Schmidt, D.; Schollmeyer, D.; Erkel, G.; Anke, T.; Kleinert, H.; Förstermann, U.; Kunz, H. Inhibitors of Inducible NO Synthase Expression: Total Synthesis of (S)-Curvularin and Its Ring Homologues. *ChemMedChem* **2008**, *3*, 924–939.
14. Winkler, D.E.; Whetstone, R.R. Some Observations on the Pechmann Reaction1. *J. Org. Chem.* **1961**, *26*, 784–787.
15. Thompson, C.M.; Orellana, M.D.; Lloyd, S.E.; Wu, W. Stereospecific Synthesis of *cis*-Stilbenes from Benzaldehydes and Phenylacetic Acids via Sequential Perkin Condensation and Decarboxylation. *Tetrahedron Lett.* **2016**, *57*, 4866–4868.
16. Durola, F.; Hanss, D.; Roesel, P.; Sauvage, J.-P.; Wenger, O.S. A New Family of Biisoquinoline Chelates. *Eur. J. Org. Chem.* **2007**, *2007*, 125–135.
17. Hume, P.A.; Furkert, D.P.; Brimble, M.A. Total Synthesis of Virgatolide B. *Org. Lett.* **2013**, *15*, 4588–4591.
18. Lypson, A.B.; Wilcox, C.S. Synthesis and NMR Analysis of a Conformationally Controlled β-Turn Mimetic Torsion Balance. *J. Org. Chem.* **2017**, *82*, 898–909.
19. Holmbo, S.D.; Pronin, S.V. A Concise Approach to Anthraquinone–Xanthone Heterodimers. *J. Am. Chem. Soc.* **2018**, *140*, 5065–5068.
20. Opatz, T.; Kauhl, U. Totalsynthese und Strukturaufklärung des (–)-Hymenosetins und verwandter 3-Decalinoyltetramsäuren: Strukturaufklärung biologisch aktiver Naturstoffe. **2017**, PhD thesis, Johannes Gutenberg University, Department of Chemistry, Mainz (http://doi.org/10.25358/openscience-3844).
